# Self‐Assembled Anion‐Binding Cryptand for the Selective Liquid–Liquid Extraction of Phosphate Anions

**DOI:** 10.1002/anie.202009960

**Published:** 2020-09-02

**Authors:** Rebecca Andrews, Sabera Begum, Christopher J. Clemett, Robert A. Faulkner, Michael L. Ginger, Jane Harmer, Marco Molinari, Gareth M. B. Parkes, Zuhlqurnain M. H. Qureshi, Craig R. Rice, Michael D. Ward, Howard M. Williams, Philippe B. Wilson

**Affiliations:** ^1^ Department of Chemical Sciences University of Huddersfield Huddersfield HD1 3DH UK; ^2^ Department of Chemistry University of Warwick Coventry CV4 7AL UK; ^3^ School of Animal Rural and Environmental Sciences Nottingham Trent University Nottingham NG25 0QF UK

**Keywords:** anions, copper, macrocycles, self-assembly, structure elucidation

## Abstract

The ligands **L^1^** and **L^2^** form trinuclear self‐assembled complexes with Cu^2+^ (i.e. [(**L^1^**)_2_Cu_3_]^6+^ or [(**L^2^**)_2_Cu_3_]^6+^) both of which act as a host to a variety of anions. Inclusion of long aliphatic chains on these ligands allows the assemblies to extract anions from aqueous media into organic solvents. Phosphate can be removed from water efficiently and highly selectively, even in the presence of other anions.

## Graphical abstract

The ligands **L^1^** and **L^2^** form trinuclear self‐assemblies with Cu^2+^ (i.e. [**L**_2_Cu_3_]^6+^) both of which act as hosts to a variety of anions. Inclusion of long aliphatic chains on these ligands allows the self‐assemblies to extract anions from aqueous media into organic solvents. Phosphate anions can be removed from water efficiently and highly selectively in the presence of other anions.

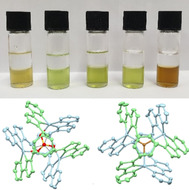

Modern agriculture is totally reliant on phosphate for the mass production of foodstuffs and annual demand for phosphates is growing twice as fast as the growth in human population.^[1]^ The “peak phosphate problem” concerns the dependency on phosphate fertilizer which, unlike nitrogen based fertilizer, is produced via a finite supply located in only a few countries. It is believed that supply will outstrip demand in 20 to 30 years with depletion of reserves in the next 50 to 100 years.^[2]^ Correspondingly, green and sustainable methodologies for phosphate use need to be developed to avoid a decrease in food production.^[3]^ Conversely, inefficient use of phosphate results in 50 Tg yr^−1^ of this fertilizer entering worldwide water sources which leads to pollution of rivers and oceans, causing toxic algal blooms and eutrophication.^[4]^

Despite their obvious medical, environmental and agricultural significance, the ability to detect and sequester anions has significantly lagged behind recognition of their cationic counterparts.^[5]^ As a result much effort has been focused upon anion recognition in supramolecular chemistry, and the ability to synthesize receptors capable of binding anions has become increasingly more understood.^[6]^ Anion receptors are generally organic scaffolds that contain functional groups capable of interacting with anions and often contain amine, amide and alcohol groups along with other hydrogen‐bond donor units. Self‐assembly is an attractive alternative to covalent synthesis for these scaffolds as it allows the construction of structurally complex architectures from relatively simple subunits, and self‐assembled hosts for anion binding have attracted a lot of recent attention.[^7^, ^8^]

Recently we have shown that the tripodal ligand **L^1^** (Figure 1) self‐assembles with Cu^2+^ ions to form the trinuclear species [(**L^1^**)_2_Cu_3_]^6+^.^[9]^ This complex contains a cavity which incorporates six ‐NH donor atoms and three Cu^2+^ metal ions in an arrangement that allows all of them to interact with anions. It was demonstrated that anions are encapsulated both in the solid‐state and aqueous systems and, upon encapsulation, spherical, trigonal planar, tetrahedral and octahedral anions are all precipitated from solution and can be removed by filtration. Furthermore, the cavity is selective for phosphate anions and precipitates these from water, reducing the concentration from 1000 to <0.1 ppm and recovering ≈99 % of the phosphate anion.^[9]^

**Figure 1:**
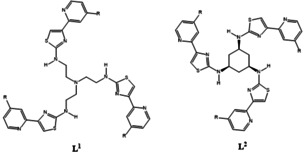
The ligands **L^1^** (R=H), **L^1a^** (R=‐CH_2_CO_2_(CH_2_)_4_CH_3_), **L^2^** (R=H), and **L^2a^** (R=‐CH_2_CO_2_(CH_2_)_4_CH_3_).

Whilst this is a promising method for phosphate recovery, anion extraction by precipitation is a batch‐based process and if removal/recovery of phosphate is to be practical, a method of anion sequestration using liquid‐liquid extraction is essential, as this can be more readily developed into a continuous process. Whilst this would be an attractive industrial process, it is complicated by the hydrophilicity of the phosphate anion which makes it difficult to extract into organic solvents, especially in comparison to the less hydrophilic anions (e.g. halides, nitrate etc).^[10]^ As a result, the ability to selectively extract phosphate into organic solvents that partition with aqueous media is challenging but important, if resource recovery is to be an achievable goal.

In this work we show that the organic backbone of **L^1^** can be changed (*cf*. the cyclohexyl linked ligand **L^2^**) which gives a modified ligand that still forms a trinuclear species [(**L^2^**)_2_Cu_3_]^6+^ which acts as a host to anionic guests in a variety of media (Figure 1). Furthermore, ligands **L^1a^** and **L^2a^** are based on their parent species (**L^1^** and **L^2^**) but contain aliphatic hexyl esters which allow the formation of the tripodal complexes (e.g. [**L**_2_Cu_3_]^6+^) in organic solvents. Both of these trinuclear assemblies extract phosphate anions from water into dichloromethane and are highly selective for Na_2_HPO_4_ in the presence of other common anions.

Ligand **L^2^** was prepared from *cis*, *cis*‐1,3,5‐triaminocyclohexane by reaction with benzoyl isothiocyanate, hydrolysis to the trithiourea and reaction with 2‐(α‐bromoacetyl)pyridine.[^8^, ^9^] Reaction of this tripodal ligand with Cu(ClO_4_)_2_ in MeNO_2_ gave a pale blue solution which turned yellow upon reaction with Bu_4_NHSO_4_ and deposited yellow crystals upon slow diffusion of diethyl ether. In the solid‐state each of the three bidentate pyridyl‐thiazole domains on the ligand strand coordinates to a different copper metal ion, and each metal ion is coordinated by two separate ligands resulting in coordination by a total of four N‐donor atoms, one bidentate pyridyl‐thiazole site from each ligand (Figure 2 a–c). In the center of the trinuclear assembly is a cavity which contains an encapsulated SO_4_^2−^ anion. This single anion is held within the host by a total of nine interactions, comprising three Cu⋅⋅⋅O coordination bonds and six −NH⋅⋅⋅O hydrogen bonding interactions. In the complex each of the three copper ions coordinates one of the oxygen atoms and this interaction is supplemented by a −NH⋅⋅⋅O hydrogen‐bond from the amine units on the ligand chain (Figure 2 d). The uncoordinated oxygen atom points upward from the trimetallic core and interacts with three remaining‐NH units. The coordination of the metal ions and the encapsulation of the sulfate is very similar to that of [(**L^1^**)_2_Cu_3_(SO_4_)]^4+^, with the Cu⋅⋅⋅OSO_3_ range fairly similar (Cu⋅⋅⋅OSO_3_ range 2.094–2.192 Å for [(**L^2^**)_2_Cu_3_(SO_4_)]^4+^ vs. 2.119–2.166 Å for [(**L^1^**)_2_Cu_3_(SO_4_)]^4+^). However, the −NH⋅⋅⋅O hydrogen‐bond interactions are substantially shorter for [(**L^2^**)_2_Cu_3_(SO_4_)]^4+^ (−NH⋅⋅⋅OSO_3_ range 1.996–2.145 Å for [(**L^2^**)_2_Cu_3_(SO_4_)]^4+^ vs. 2.255–2.286 Å for [(**L^1^**)_2_Cu_3_(SO_4_)]^4+^). This reduction in distances is a consequence of the spacer unit, as the 1,3,5‐cyclohexyl spacer of **L^2^** is dimensionally smaller than the trimethylamine unit of **L^1^**, with the amine units separated by 4 bonds in **L^2^** as opposed to 6 bonds in **L^1^**. The host‐guest complex is also observed in the gas phase with ions in the ESI‐MS at *m*/*z* 1952 and 902 corresponding to {[(**L^2^**)_2_Cu_3_(SO_4_)](OTf)_3_}^+^ and {[(**L^2^**)_2_Cu_3_(SO_4_)](OTf)_2_}^2+^ respectively.

**Figure 2:**
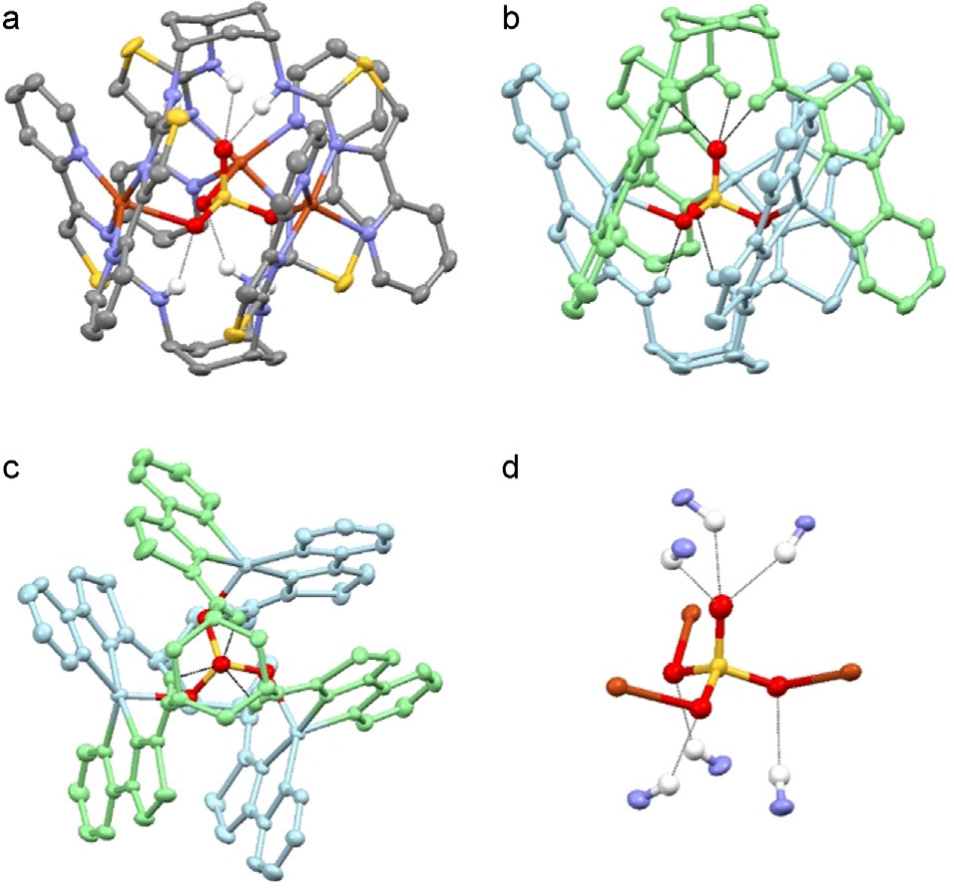
a–d) Single‐crystal X‐ray structure of [(**L^2^**)_2_Cu_3_(SO_4_)]^4+^ showing the encapsulation and hydrogen bonding of the anion. Thermal ellipsoids are shown at the 50 % probability level. Selected hydrogen atoms and anions are omitted for clarity. Color code: orange, Cu^2+^; red, O; blue, N; yellow, S; grey, C (apart from **2 b** and **2 c** where the ligands have been colored for clarity).

Reaction of [(**L^2^**)_2_Cu_3_]^6+^ with Bu_4_NBr in water and acetone (1:3) gave a blue solution from which blue block shaped crystals were formed upon slow evaporation. In the solid‐state the trinuclear species persists and encapsulated within this core is a bromide ion, that is, the complex is [(**L^2^**)_2_Cu_3_Br]^5+^. As in the previous example of the sulfate complex, the halide anion interacts with all three of the Cu^2+^ ions and is supplemented by six −NH⋅⋅⋅Br hydrogen‐bonding interactions (see ESI).

As can been seen in the two solid‐state structures the cyclohexyl‐based ligand **L^2^** behaves in a similar fashion to **L^1^**: *viz*. it forms the trimetallic self‐assembly and incorporates anions within the cryptand. Whilst we don't have solid‐state evidence for encapsulation of anions, other than sulfate and bromide, it seems highly likely that other tetrahedral oxoanions and halides would be bound in the cavities of complexes with **L^2^** an analogous fashion, as has been extensively observed in **L^1^**.

In an effort to produce compounds that could abstract anions from aqueous media into an organic solvent, ligands **L^1a^** and **L^2a^** were prepared, each containing three ‐CH_2_O_2_C(CH_2_)_4_CH_3_ ester units attached to the pyridyl units at the C^4^ position. The inclusion of these units should increase the solubility of the complexes in organic solvents, but are sufficiently remote from the metal binding sites not to interfere with either the self‐assembly of the complex or its ability to act as a host for anions. Reaction of either **L^1a^** and **L^2a^** with Cu(OTf)_2_ in DCM (containing 3 % MeOH) gave dark‐yellow or pale‐yellow solutions, respectively (Figure 3 a). Partitioning of either of these solutions with water containing one equivalent of NaH_2_PO_4_ resulted in a color change from yellow to lime green within minutes: a color change indicative of phosphate encapsulation (Figure 3 b). UV/Vis studies show that binding of anions occurs in organic solvent as addition of one equivalent of a range of anions (as their tetraalkylammonium salts) to either [(**L^1a^**)_2_Cu_3_](OTf)_6_ or [(**L^2a^**)_2_Cu_3_](OTf)_6_ in DCM does result in a change in the UV/Vis spectrum with chloride, bromide, sulfate and phosphate but not with nitrate. Furthermore, examination of the organic layer from the biphasic system by ESI‐MS showed ions at *m*/*z* 2606 and 1228 (for **L^1a^**) and *m*/*z* 2572 and 1211 (for **L^2a^**) corresponding to {[(**L**)_2_Cu_3_(PO_4_)](OTf)_2_}^+^ and {[(**L**)_2_Cu_3_(PO_4_)](OTf)}^2+^ for each ligand, respectively. This indicates that the trinuclear assembly persists in DCM and that the phosphate can be transferred, via incorporation into the host assembly, to the organic phase.^[11]^

**Figure 3:**
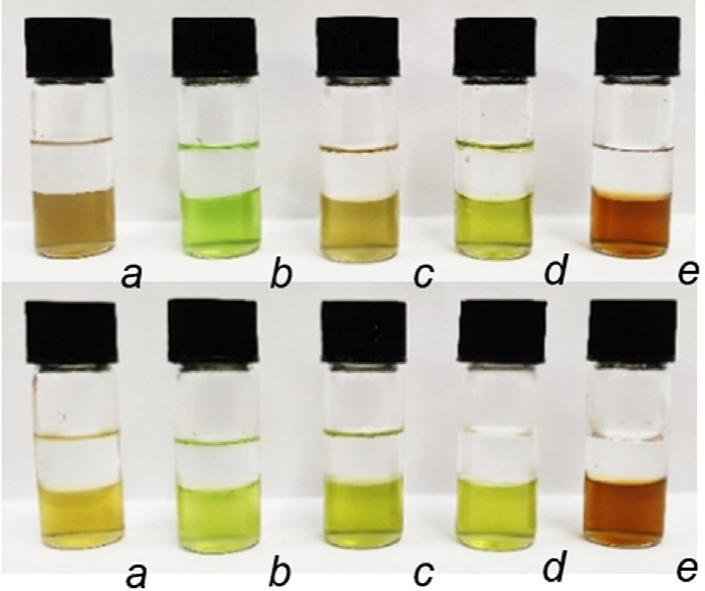
Extraction experiments of a DCM solution of [(**L^1a^**)_2_Cu_3_]^6+^ (top) and [(**L^2a^**)_2_Cu_3_]^6+^ (bottom) with a) ultrapure water and aqueous solutions containing b) one equivalent of Na_2_HPO_4_, c) one equivalent of each of NaCl, NaNO_3_, NaHSO_4_ and NaH_2_PO_4_, d) one equivalent of each of NaCl, NaNO_3_, Na_2_SO_4_ and Na_2_HPO_4_, and e) one equivalent of each of NaF, NaCl, NaBr and NaI.

In an effort to ascertain how much of the phosphate anion was transferred to the organic phase a series of extraction experiments were carried out and the amount of anion remaining in the aqueous solution examined by ion chromatography (Table 1). In this experiment 3 mL of 3 % MeOH in DCM containing 4.95 μmols of either [(**L^1a^**)_2_Cu_3_]^6+^ or [(**L^2a^**)_2_Cu_3_]^6+^ (1.6 mm) was exposed to water containing 4.95 μmols of Na_2_HPO_4_, and the biphasic system was stirred for 18 hrs (with a noticeable colour change after 1 h). After this time 2 mL of the aqueous layer was removed and the volume accurately adjusted to 5 mL; giving a theoretical concentration of 0.66 mm if none of the Na_2_HPO_4_ had been consumed.

**Table 1:** Percentage remaining Na_2_HPO_4_ from aqueous solutions exposed to an organic solution of either [(**L^1a^**)_2_Cu_3_]^6+^ or [(**L^2a^**)_2_Cu_3_]^6+^.

Equivalents of Host	[(**L^1a^**)_2_Cu_3_](OTf)_6_ [%]	[(**L^2a^**)_2_Cu_3_](OTf)_6_ [%]
1	18	24
1.1	11	20
1.2	6	14

Concentration of the phosphate anion measured by ion chromatography.

When a stoichiometric amount of either [(**L^1a^**)_2_Cu_3_]^6+^ or [(**L^2a^**)_2_Cu_3_]^6+^ is used the majority of hydrogen phosphate anion is extracted from the aqueous phase (82 % and 76 % respectively); using a modest 1.2‐fold excess of the host removed 94 % and 86 % of Na_2_HPO_4_ for [(**L^1a^**)_2_Cu_3_]^6+^ or [(**L^2a^**)_2_Cu_3_]^6+^ respectively (Table 1). As a result both complexes are efficient at removal of phosphate from water with the **L^1a^** complex extracting ca. 10 % more of the anion under these conditions than the complex of **L^2a^**. Correspondingly, the phosphate anion is replaced by three of the mono‐anions present on the host complex (e.g. [(**L^1a^**)_2_Cu_3_](OTf)_6_ forms [(**L^1a^**)_2_Cu_3_(PO_4_)](OTf)_3_ plus three equivalents of triflate). However, these assemblies can be made using a variety of anions and a relatively environmentally benign anion (e.g. acetate) could be used in the exchange process.

A series of competitive experiments was carried out to investigate the selectivity of the assemblies to common anions. 3 mL of 3 % MeOH in DCM containing 4.95 μmol of either [(**L^1a^**)_2_Cu_3_]^6+^ or [(**L^2a^**)_2_Cu_3_]^6+^ (1.6 mm) was exposed to water containing a mixture of NaCl, NaNO_3_, NaHSO_4_ and NaH_2_PO_4_ (4.95 μmols of each) and the biphasic system was stirred for 18 hrs. As described previously the aqueous solution was analyzed for residual anion content by ion chromatography (Table 2).

**Table 2:** Percentage remaining of a solution of different anions from an aqueous solution exposed to an organic solution of either [(**L^1a^**)_2_Cu_3_]^6+^ or [(**L^2a^**)_2_Cu_3_]^6+^.

Complex	NaCl [%]	NaNO_3_ [%]	NaHSO_4_ [%]	NaH_2_PO_4_ [%]
[(**L^1a^**)_2_Cu_3_](OTf)_6_	110^[a]^	103	47	73
[(**L^2a^**)_2_Cu_3_](OTf)_6_	68^[b]^	99	61	62

[a] Excess chloride anions are probably due to impurities from the other salts and solvents. [b] The removal is possibly slightly higher than reported due to excess chloride from contamination. Concentration of anions measured by ion chromatography.

For [(**L^1a^**)_2_Cu_3_]^6+^ no chloride or nitrate anions are removed from the aqueous layer, but removal of both NaHSO_4_ and NaH_2_PO_4_ occurs with the former being removed more effectively. For the **L^2a^** assembly there is no preference between NaHSO_4_ and NaH_2_PO_4,_ but this cage also removes chloride from the system and it shows similar affinity for both phosphate and sulfate.

The same experiment was carried out on the extraction systems but the disodium salts Na_2_SO_4_ and Na_2_HPO_4_ were used instead of their monosodium analogues (Table 3).

**Table 3:** Percentage remaining of a solution of different anions from an aqueous solution exposed to an organic solution of either [(**L^1a^**)_2_Cu_3_]^6+^ or [(**L^2a^**)_2_Cu_3_]^6+^.

Complex	NaCl [%]	NaNO_3_ [%]	Na_2_SO_4_ [%]	Na_2_HPO_4_ [%]
[(**L^1a^**)_2_Cu_3_](OTf)_6_	107^[a]^	101	85	41
[(**L^2a^**)_2_Cu_3_](OTf)_6_	97^[b]^	100	95	36

[a] Excess chloride anions are probably due to impurities from the other salts and solvents. [b] The removal is possibly slightly higher than reported due to excess chloride from contamination. Concentration of anions measured by ion chromatography.

This data shows a very different trend from the previous results with host [(**L^1a^**)_2_Cu_3_]^6+^ showing a preference for phosphate, removing 59 % of this anion from solution compared to only 15 % of sulfate. Remarkably [(**L^2a^**)_2_Cu_3_]^6+^ removes no nitrate and only a small amount of either chloride or sulfate anions. but it removed 64 % of Na_2_HPO_4_ demonstrating significant selectivity for this anion. The difference in selectivity between Na_2_HPO_4_/NaH_2_PO_4_ and Na_2_SO_4_/NaHSO_4_ can be rationalized by the acidity of the mono‐anion. In these systems once encapsulation has occurred the anions are fully deprotonated regardless of their original protonated state (c.f. reaction of [(**L^2^**)_2_Cu_3_]^6+^ with Bu_4_NHSO_4_ giving [(**L^2^**)_2_Cu_3_(SO_4_)]^4+^). The monoanionic HSO_4_^−^ (p*K*_a_=1.81) is significantly more acidic than H_2_PO_4_^−^ (p*K*_a_=7.21) and as a result HSO_4_^−^ is more readily deprotonated and will occupy the cavity in preference to dihydrogen phosphate. This issue does not arise with the dianionic SO_4_^2−^ and the cavity is selective for the phosphate (due to the difference in anionic charge) demonstrating that the selectivity of anion encapsulation, and extraction into organic solutions, can be controlled by pH.^[12]^

Molecular modelling of these systems shows that there is a thermodynamic preference for both the self‐assembled species [(**L^1^**)_2_Cu_3_]^6+^ and [(**L^2^**)_2_Cu_3_]^6+^ to act as hosts for anionic guests but the former (based on **L^1^**) gives stronger anion binding than the latter (based on **L^2^**). The formation energies of the complexes are −1197 kJ mol^−1^ [(**L^1^**)_2_Cu_3_(PO_4_)]^3+^, −1030 kJ mol^−1^ [(**L^2^**)_2_Cu_3_(PO_4_)]^3+^, −1750 kJ mol^−1^ [(**L^1^**)_2_Cu_3_(SO_4_)]^4+^, and −478 kJ mol^−1^ [(**L^2^**)_2_Cu_3_(SO_4_)]^4+^. Based on the calculated energetics of the systems, the ratios of phosphate to sulphate distribution are predicted to be 54:46 for [(**L^1^**)_2_Cu_3_(PO_4_)]^3+^ and [(**L^2^**)_2_Cu_3_(PO_4_)]^3+^ and 79:21 for [(**L^1^**)_2_Cu_3_(SO_4_)]^4+^ and [(**L^2^**)_2_Cu_3_(SO_4_)]^4+^. Compared to [(**L^2^**)_2_Cu_3_]^6+^, [(**L^1^**)_2_Cu_3_]^6+^ shows a marginal preference for phosphate and but a much greater preference for sulphate. Comparison between the energies of the phosphate and sulfate host‐guest complexes with each different ligand system shows that the distribution of phosphate and sulphate in the presence of [(**L^1^**)_2_Cu_3_]^6+^ is 41:59, which indicates a thermodynamic preference for this system to act as a host for sulphate compared to phosphate (e.g. [(**L^1^**)_2_Cu_3_(SO_4_)]^4+^ has a more negative free energy of formation than [(**L^1^**)_2_Cu_3_(PO_4_)]^3+^). The distribution of phosphate and sulphate inclusion in the presence of [(**L^2^**)_2_Cu_3_]^6+^ is 68:32, which indicates a thermodynamic for the formation of [(**L^2^**)_2_Cu_3_(PO_4_)]^3+^ over [(**L^2^**)_2_Cu_3_(SO_4_)]^4+^, the opposite selectivity of the **L^1^** system.

The modelling results are in good agreement with the experimental results, which show that the extraction of phosphate from water with [(**L^1a^**)_2_Cu_3_]^6+^ is approximately 10 % greater than with [(**L^2a^**)_2_Cu_3_]^6+^ Extraction of NaCl, NaNO_3_, NaHSO_4_ and NaH_2_PO_4_ shows a clear preference for the extraction of sulfate using [(**L^1a^**)_2_Cu_3_]^6+^, but little difference between NaHSO_4_ and NaH_2_PO_4_ with [(**L^2a^**)_2_Cu_3_]^6+^. For the extraction of Na_2_SO_4_ and Na_2_HPO_4,_ both complexes have a preference for phosphate, but this preference is more pronounced for [(**L^2a^**)_2_Cu_3_]^6+^.

The subtle differences in the extraction ability of the two hosts [(**L^1a^**)_2_Cu_3_]^6+^ and [(**L^2a^**)_2_Cu_3_]^6+^ is a probable consequence of the different sizes of the cavities formed. Even though PO_4_^3−^ is slightly larger than SO_4_^2−^ (1.54 Å vs. 1.49 Å respectively^13^) the rigidity and smaller size of the cyclohexyl spacer unit, which induces shorter Cu⋅⋅⋅anion and −NH⋅⋅⋅anion distances, may have a better size match to the phosphate and account for the greater selectivity of this anion.

In conclusion, we have shown that self‐assembled systems with differing spacer units (*viz*. [(**L^1^**)_2_Cu_3_]^6+^ and [(**L^2^**)_2_Cu_3_]^6+^) can act as anion binding hosts exhibiting differing selectivities for different anions. Inclusion of long chain ester units on these ligands can make the complexes soluble in organic solvents and these are capable of liquid‐liquid extraction of phosphate anions from water, showing excellent selectivity in the presence of other common anions. Indeed, extraction experiments on Basel growth medium (BBM, an algal growth medium)^[14]^ which contains a variety of nutrients (and possible interferences) demonstrates that the phosphate anion concentration can be reduced from 168 ppm to <5 ppm but chloride (27.5 ppm) and nitrate (521 ppm) are hardly affected. The sulfate is also reduced (28.4 ppm to <1 ppm) which is unsurprising as the complex is used in slight excess (1.2 equivalents with respect to phosphate). This clearly shows that phosphate can be selectively removed form model aquatic systems, indicating a possible method for phosphate sequestration from eutrophic systems.

## Conflict of interest

The authors declare no conflict of interest.

## Supporting information

As a service to our authors and readers, this journal provides supporting information supplied by the authors. Such materials are peer reviewed and may be re‐organized for online delivery, but are not copy‐edited or typeset. Technical support issues arising from supporting information (other than missing files) should be addressed to the authors.

Supplementary
