# Supplementary material for: Self‐Assembled Anion‐Binding Cryptand for the Selective Liquid–Liquid Extraction of Phosphate Anions
Source: Angew Chem Int Ed Engl. 2020 Sep 2;59(46):20480–4. doi: 10.1002/anie.202009960 (PMC7693201; doi:10.1002/anie.202009960)
Supplement: Supplementary file 1 — Supplementary [file ANIE-59-20480-s001.pdf]

Angewandte Chemie
Eine Zeitschrift der Gesellschaft Deutscher Chemiker

Supporting Information

Self-Assembled Anion-Binding Cryptand for the Selective Liquid–Liquid Extraction of Phosphate Anions

Rebecca Andrews, Sabera Begum, Christopher J. Clemett, Robert A. Faulkner, Michael L. Ginger, Jane Harmer, Marco Molinari, Gareth M. B. Parkes, Zuhlqurnain M. H. Qureshi, Craig R. Rice, Michael D. Ward, Howard M. Williams, and Philippe B. Wilson*

anie_202009960_sm_miscellaneous_information.pdf

Experimental

Chemicals were purchased and used without further purification. ^1H and ^{13}C NMR spectra were recorded on a 400 MHz Bruker Avance DP X400. Mass spectra were obtained on an Agilent 6210 TOF MS for the organic species with the metal complexes run on a Bruker MicroQTOF LC. Ion chromatography was recorded on a Metrohm 850 IC system. UV-Vis spectra were run on an Agilent Cary 60 UV-Vis. The compounds *cis*,*cis*-1,3,5-triamino-cyclohexane,[S1] (2-(2-methyl-1,3-dioxolan-2-yl)pyridin-4-yl)methanol[S2] and 1,1',1"-(nitrilotris(ethane-2,1-diyl))tris(thiourea)[S3] were all produced by literature methods.

Synthesis of ligand L².

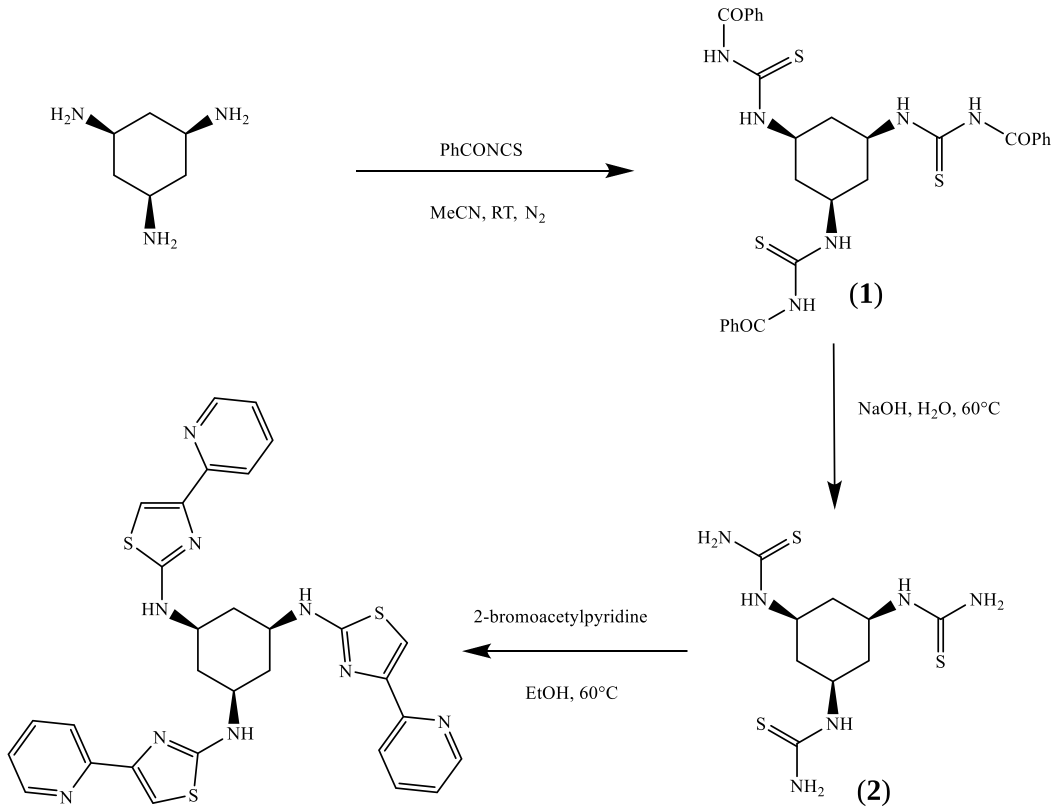

Figure 1. Synthesis of ligand **L²**.

Synthesis of **1**. To a stirred solution of *cis*,*cis*-1,3,5-triaminocyclohexane (520 mg, 4.03 mmol) in acetonitrile (50 mL) was slowly added benzoyl isothiocyanate (1.8 mL, 13.30 mmol) during which time a thick pale yellow precipitate formed. After 12 h the product was isolated by vacuum filtration and washed with a minimal amount of acetonitrile, affording the tribenzoylated triurea derivative (**1**) as an off-white powder (2.00 g, 80%). ^1H NMR (400 MHz, DMSO-d_6) δ = 11.34 (s, 3H, -CONH), 10.86 (d, 3H, J = 7.6, -CSNH), 7.83 (d, 6H, J = 7.5,

-Ph), 7.56 (t, 3H, J = 7.4, -Ph), 7.42 (t, 6H, J = 7.7, -Ph), 4.35 (m, 3H, cy-CH), 2.44 (m – coincident with solvent, 3H, broad, -CH), 1.59 (q, 3H, J = 11.2 Hz, cy-CH). ^{13}C NMR (100 MHz, DMSO-d_6) δ = 179.9 (Q, C=O), 168.8 (Q, C=S), 133.5 (CH, Ph), 132.6 (Q, Ph), 129.0 (CH, Ph), 128.9 (CH, Ph), 50.8 (CH, cy-CH), 35.0 (CH_2, cy-CH_2). ESI-MS m/z 619 (M + H^+), HR ESI-MS found 618.1536, $C_{30}H_{30}N_6S_3O_3$ require 618.1542 (error 0.86 ppm).

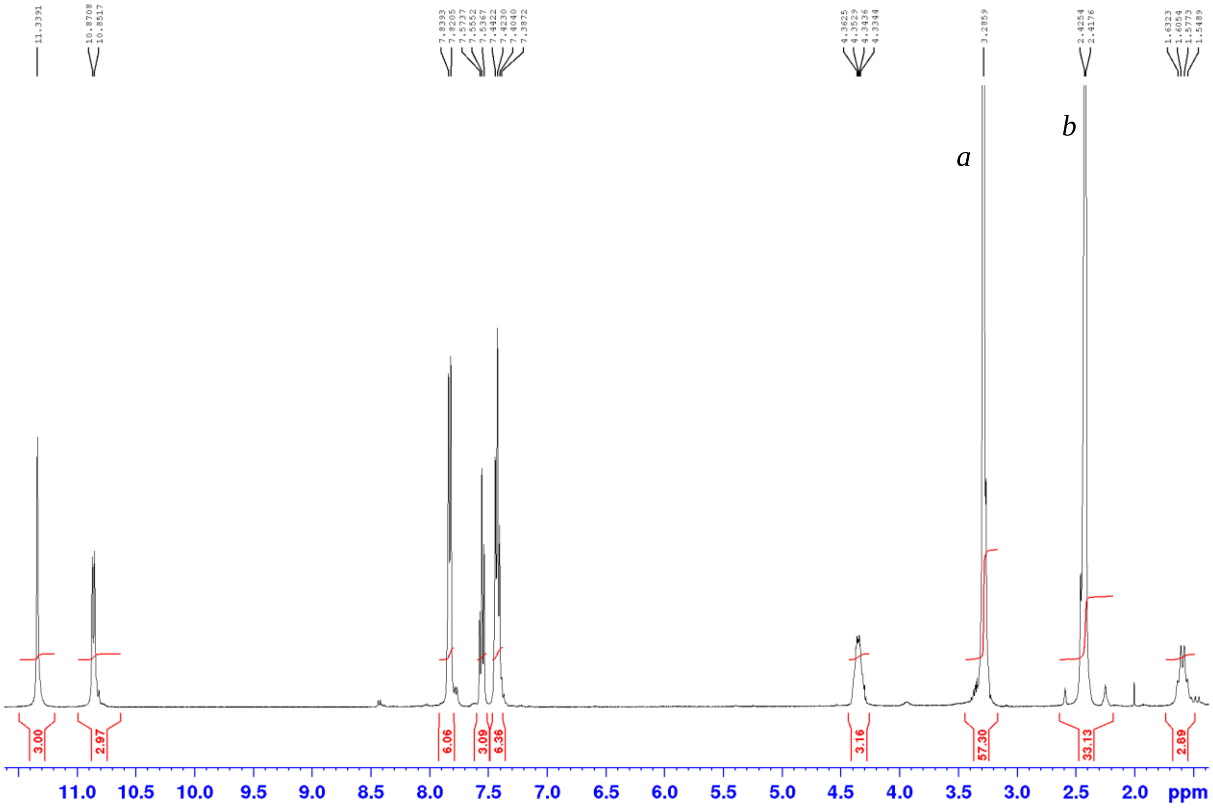

Figure 2. *^1H NMR of tribenzoylated trithiourea derivative (**1**) in DMSO-d_6. a = water impurity and b = $(CD_3)(CHD_2)SO$.*

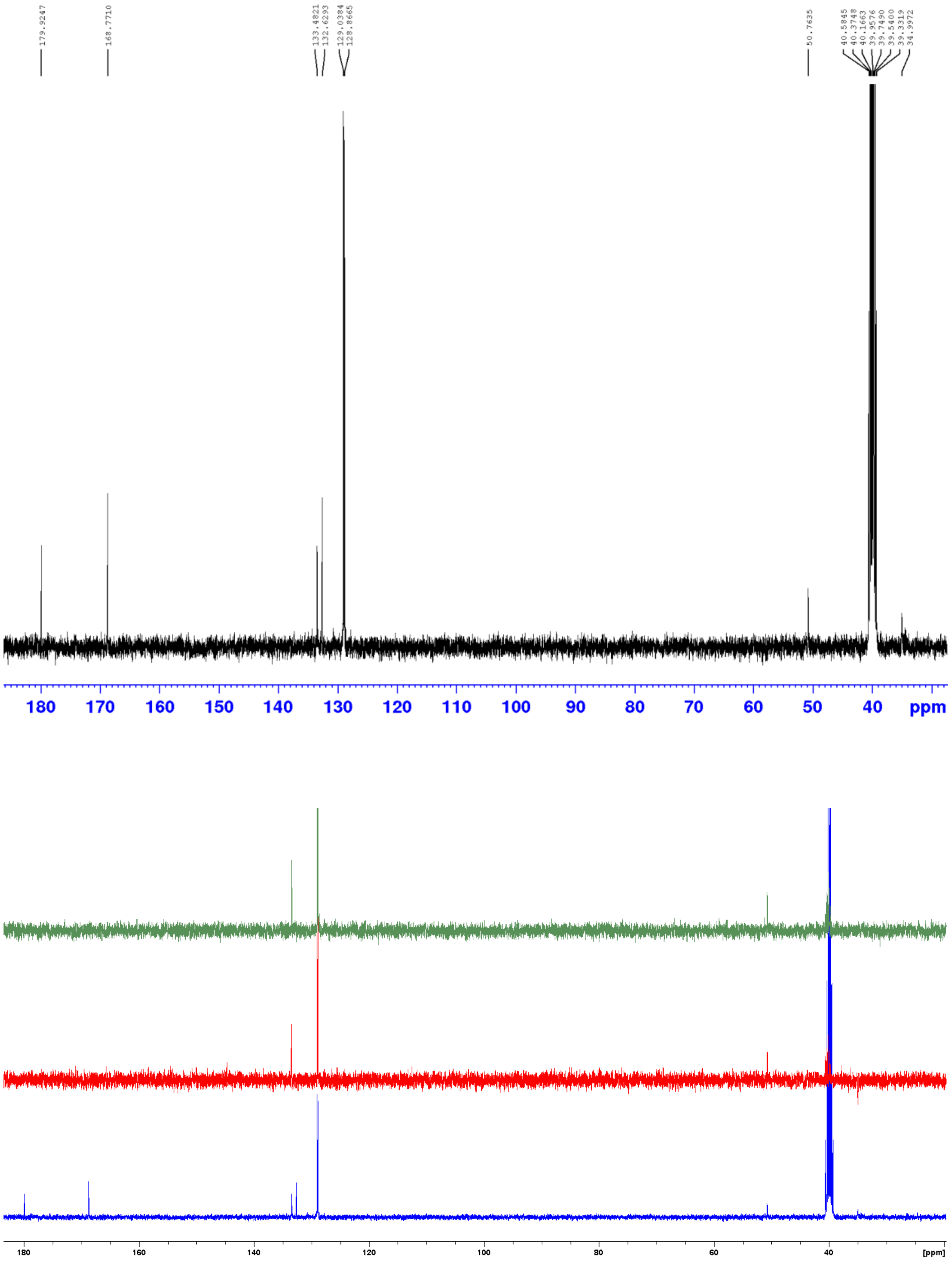

Figure 3. *^{13}C and DEPT NMR of tribenzoylated trithiourea derivative (**1**) in DMSO-d$_6$.*

Synthesis of **2**. To a stirred suspension of the tribenzoylated trithiourea (**1**) (1.88 g, 3.04 mmol) in water (40 mL) sodium hydroxide (1.22 g, 30.4 mmol) was added. The reaction was then heated at 60°C for 12 h, during which a colourless precipitate formed. After this time the mixture was allowed to cool to ambient temperature and the precipitate isolated by vacuum filtration, which was washed with water and diethyl ether, affording

the tri-thiourea derivative (**2**) as a white powder (635 mg, 68%). ^{1}H NMR (400 MHz, DMSO-d_6) δ = 7.90 – 6.70 (m, br, 9H, N*H*-C=S=N*H*$_2$), 3.98 (s, br, 3H, cy-CH), 2.24 – 1.82 (m, br, 3H, cy-CH), 1.10 (s, br, 3H, cy-CH). ESI-MS *m/z* 307 (M + H$^+$), HR ESI-MS found 306.0761, $C_9H_{18}N_6S_3$ requires 306.0755 (error 0.70 ppm). The ^{1}H NMR contains a series of broad peaks which is common with compounds that contain multiple thiourea units and arises for *intra*- and *inter*-molecular interactions.[ref] Regardless the ^{1}H NMR contains no starting material and only a minor contamination of benzoic acid and was used without purification in the next step.

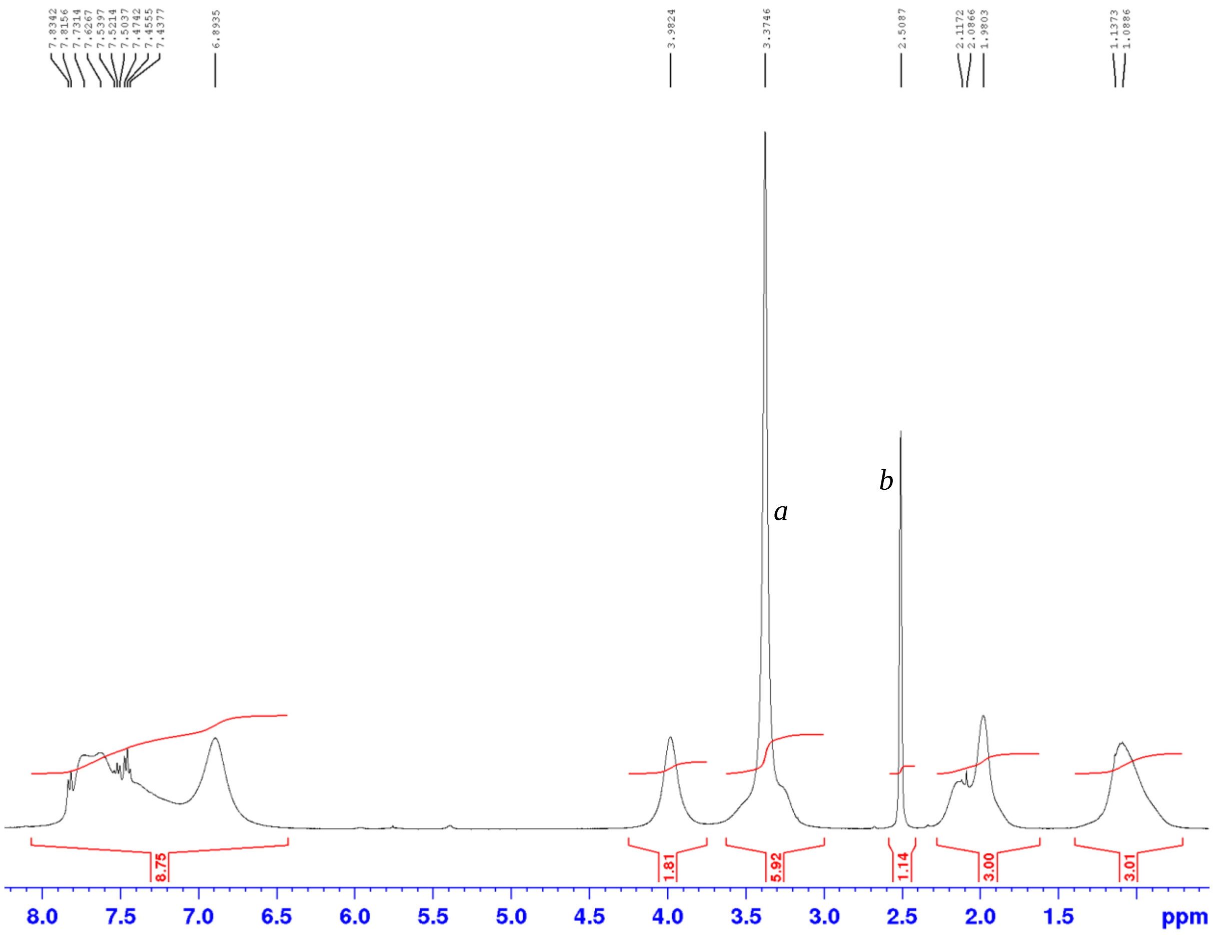

Figure 4. *^{1}H NMR of trithiourea derivative (**2**) in DMSO-d_6. a = water impurity and b = $(CD_3)(CHD_2)SO$.*

Synthesis of ligand **L^2**. To a solution of 2-(*α*-bromoacetyl)pyridine (430 mg, 2.15 mmol) in ethanol (20 mL), trithiourea **2** (200 mg, 0.654 mmol) was added and the reaction was then stirred at 60°C for 24 h. During this time a heavy yellow precipitate formed, which was isolated by vacuum filtration and washed with a minimal amount of cold ethanol. The resulting bright yellow powder was then deprotonated by stirring in an excess of concentrated ammonia at room temperature over 12 h, resulting in a colour change to pale yellow/orange. This was isolated by vacuum filtration and washed with water (3 x 10 mL) and dried under vacuum affording the product as a pale yellow solid (322mg, 81%). ^{1}H NMR (600 MHz, DMSO-d_6) δ = 8.54 (d, 3H, *J* = 4.1, py), 7.96 (d, 3H, *J* = 7.9, py), 7.85 (d, 3H, *J* = 7.3, -NH), 7.76 (t, 3H, *J* = 7.5, py), 7.36 (s, 3H, tz), 7.29 (dd, 3H, *J*

= 6.9, 5.3, py), 3.91 (m, 3H, cy-CH), 2.62 (d, 3H, J = 11.4, cy-CH), 1.29 (q, 3H, J = 11.8 Hz, cy-CH). ^{13}C NMR (150 MHz, DMSO-d_6) δ = 167.6 (Q), 152.0 (Q), 149.6 (Q), 148.8 (CH), 137.5 (CH), 122.5 (CH), 120.4 (CH), 105.3 (CH), 50.4 (CH), 37.7 (CH_2). ESI-MS m/z 306 (M + $2H^+$), HR ESI-MS found 609.1573, $C_{30}H_{27}N_9S_3$ requires 609.1552 (error 3.55 ppm).

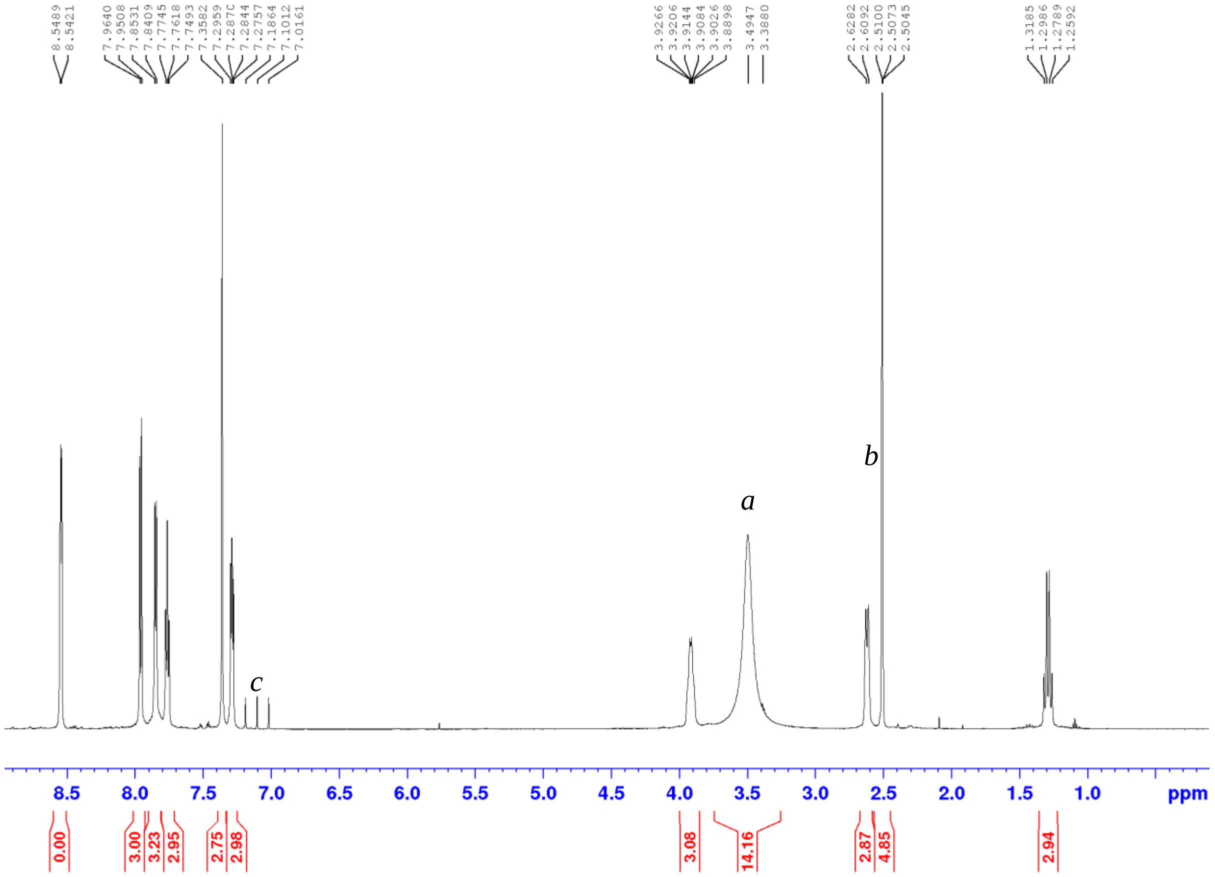

Figure 5. *^1H NMR of* ***L^2*** *in DMSO-d_6. a = water impurity, b = $(CD_3)(CHD_2)SO$ and c NH_4^+ impurity.*

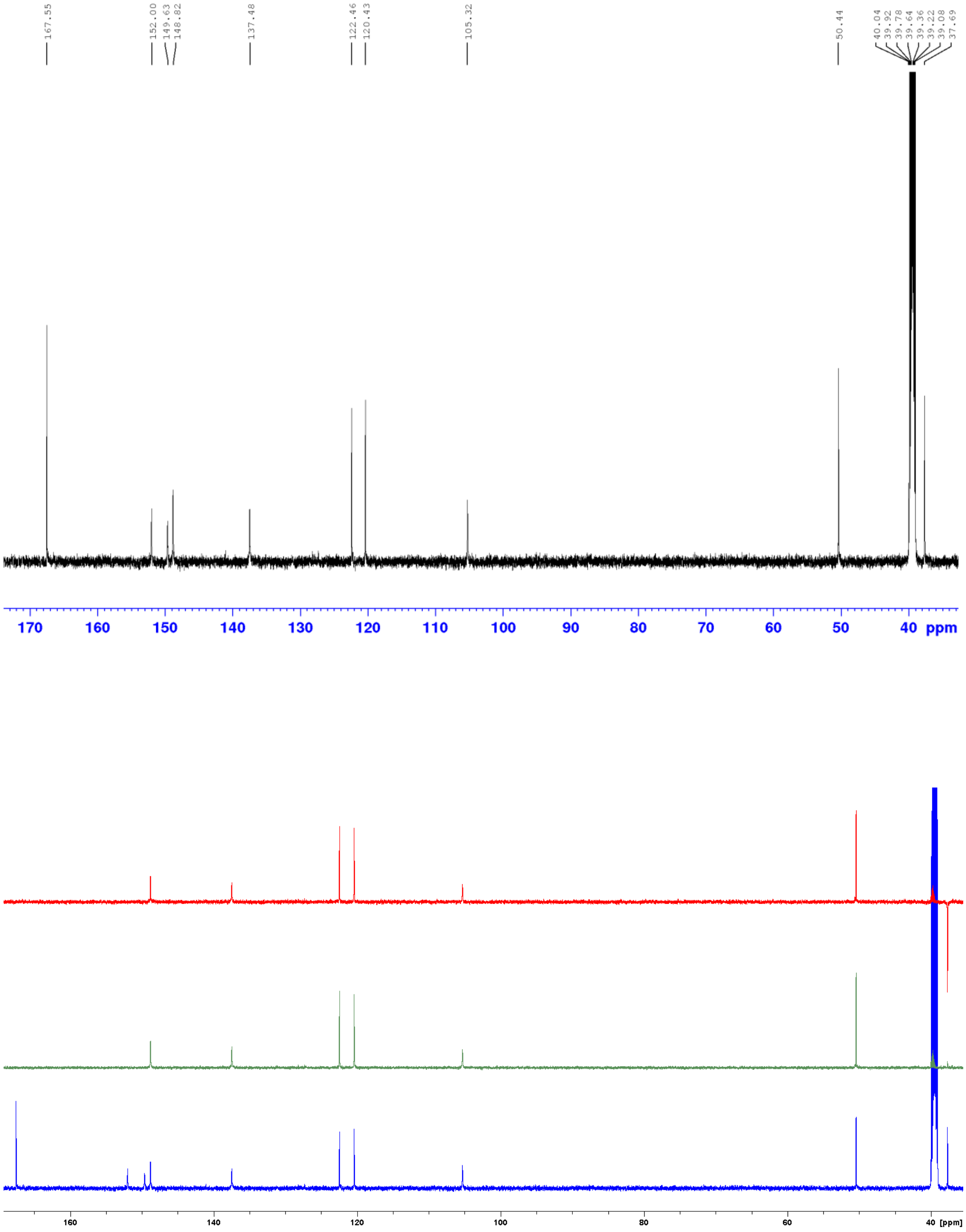

Figure 6. *^{13}C and DEPT NMR of* $\mathbf{L^2}$ *in DMSO-d_6.*

Synthesis of **L**[1a] and **L**[2a].

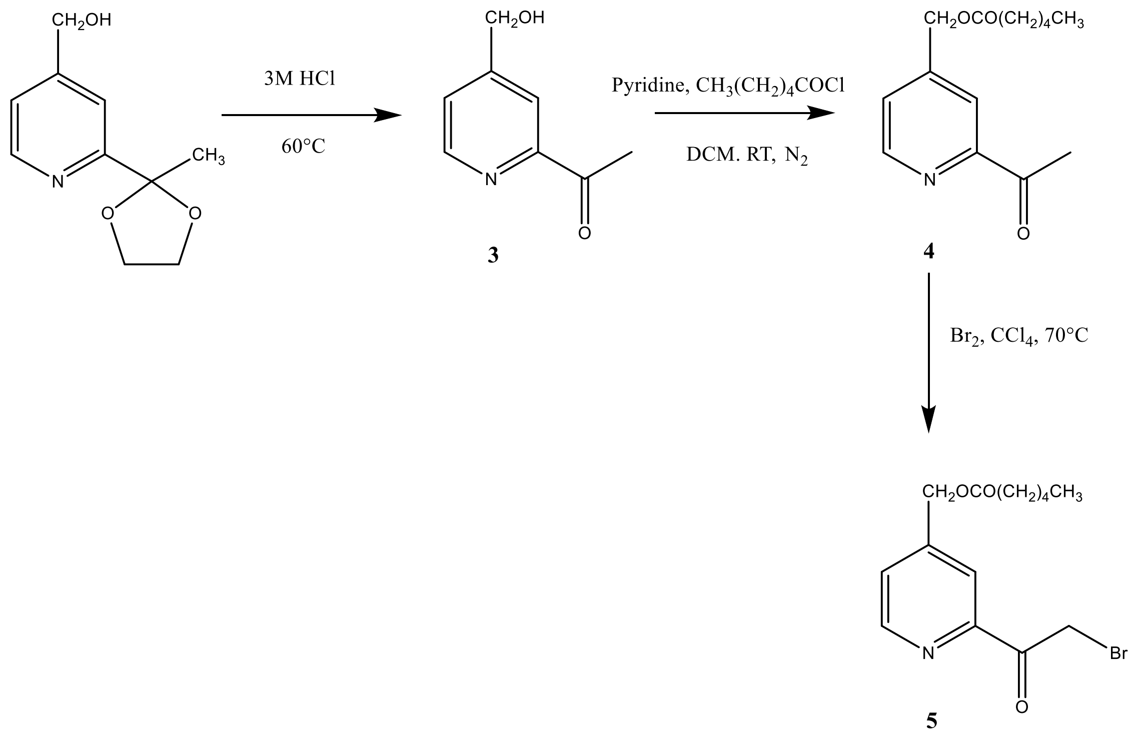

Figure 7. Synthesis of the precursors to **L**[1a] and **L**[2a].

Synthesis of 2-acetylpyridine-4-methanol (**3**). (2-(2-methyl-1,3-dioxolan-2-yl)pyridin-4-yl)methanol (1.86 g, 9.54 mmol)[ref] was stirred overnight in 3M HCl (20 mL) at 60°C. This was then cooled and pH adjusted to alkaline with solid sodium bicarbonate. This mixture was then extracted into 2% methanol in dichloromethane (4 x 100 mL), the organic layer was dried over anhydrous magnesium sulfate and solvent removed under reduced pressure, affording 2-acetylpyridine-4-methanol (**3**) as a yellow oil (1.20 g, 83%). ^{1}H NMR (300 MHz, *CDCl3)* δ (ppm) = 8.67 (d, 1H, *J* = 5.0, py), 8.02 (t, 1H, *J* = 0.8, py), 7.53 (dt, 1H, *J* = 5.0, 0.8, py), 4.83 (d, 2H, *J* = 5.9, C*H2*-OH), 2.74 (s, 3H, COC*H3*), 2.18 (t, 1H, *J* = 5.9 Hz, -OH). ^{13}C NMR (75 MHz, *CDCl3) δ* (ppm) = 200.5 (Q, *C*OCH3), 153.4 (Q), 151.6 (Q), 149.1 (CH), 124.5 (CH), 119.1 (CH), 63.0 (CH_2), 25.9 (CH_3). ESI-MS *m/z* 152 (M + H^+), HR ESI-MS found 151.0632, $C_8H_9NO_2$ requires 151.0633 (error 0.89 ppm).

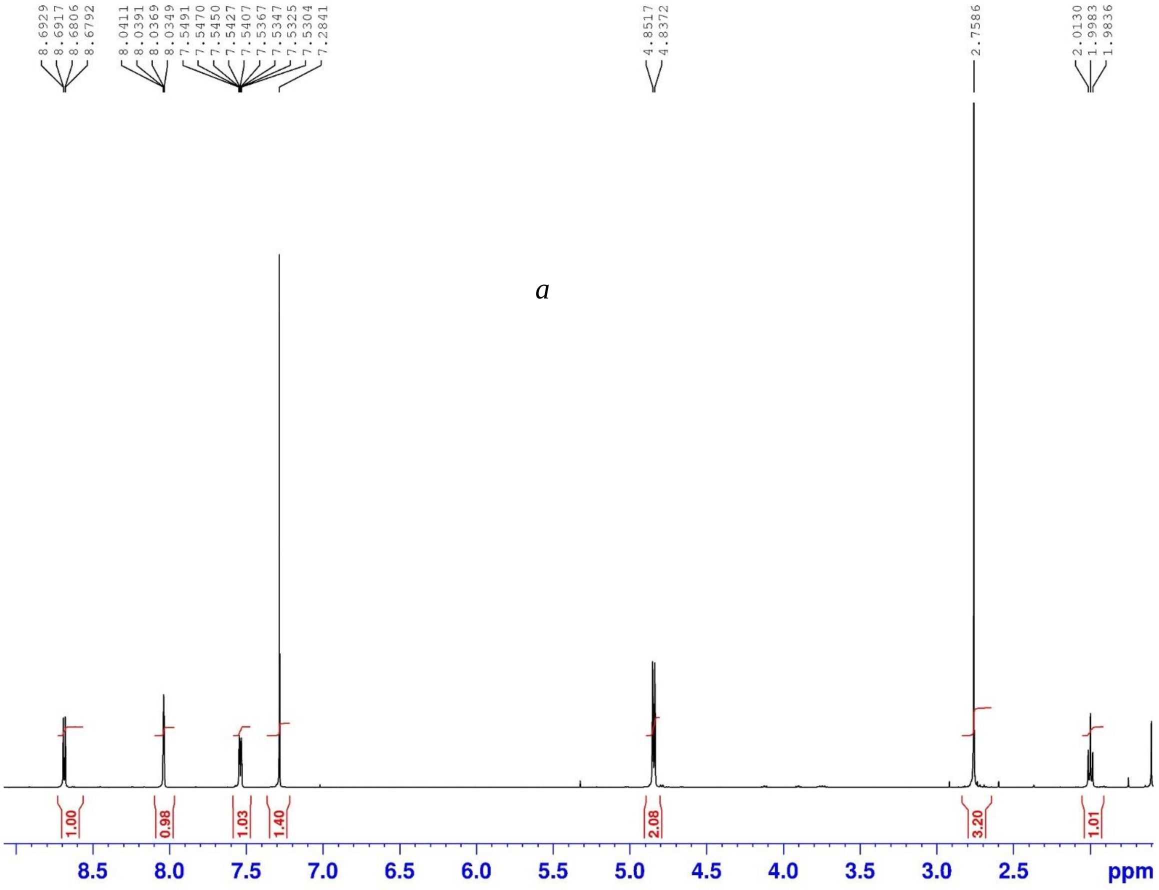

Figure 8. *^{1}H NMR of* 2-acetylpyridine-4-methanol (**3**) *in $CDCl_3$.*

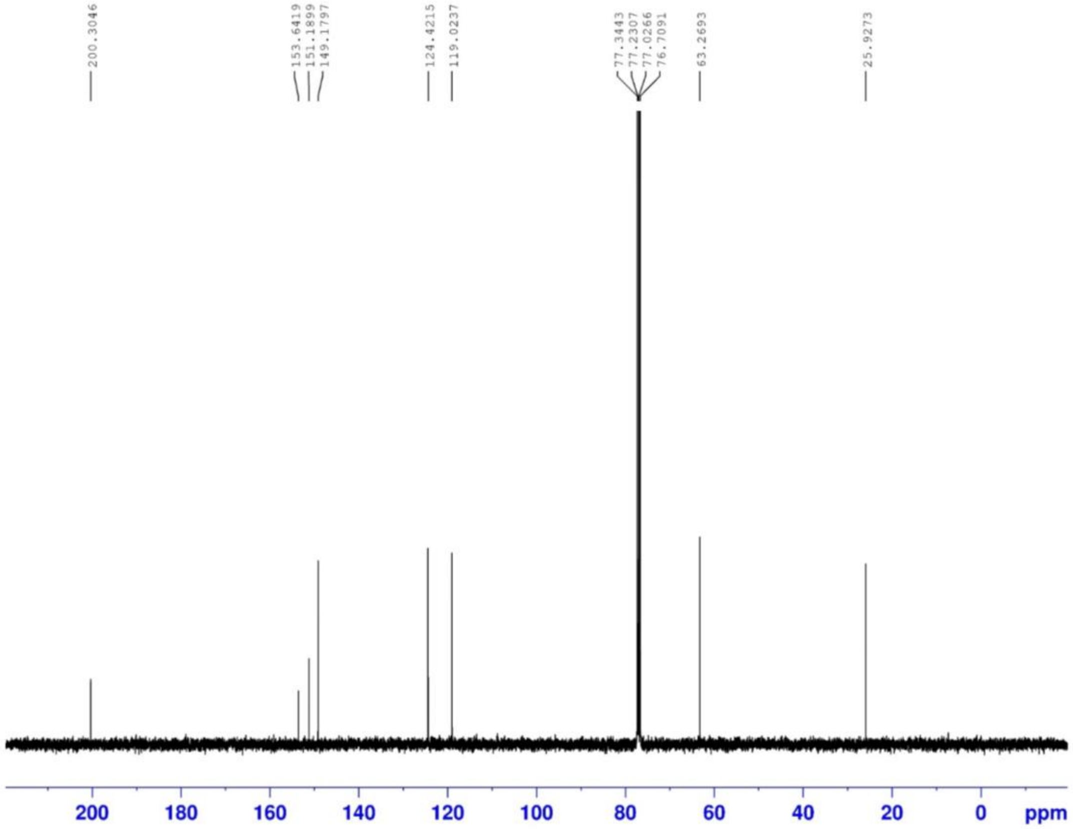

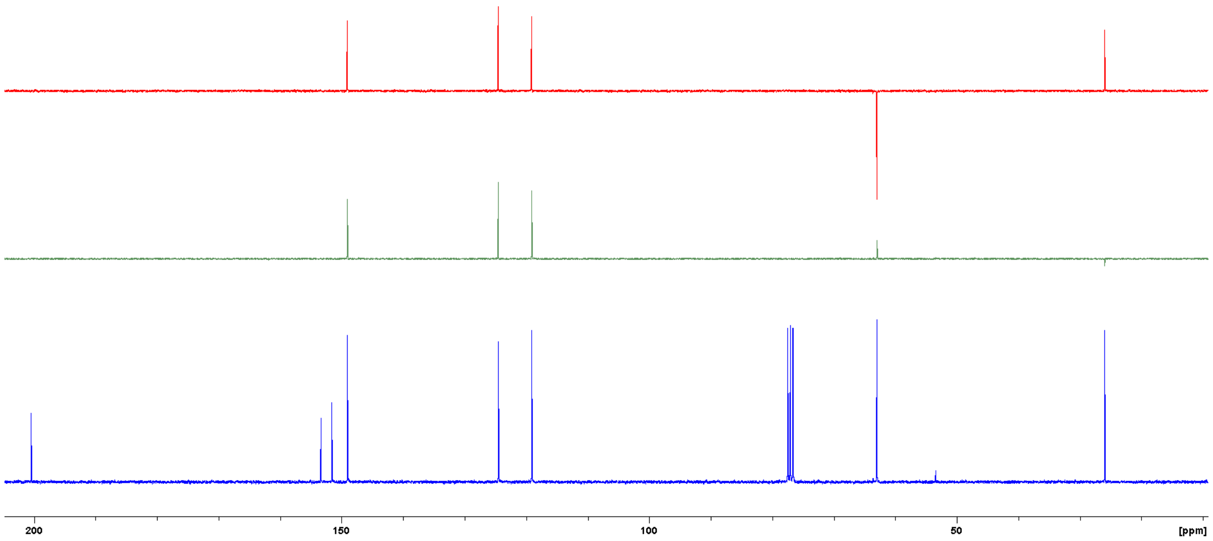

Figure 9. *^{13}C and DEPT NMR of 2-acetylpyridine-4-methanol (**3**) in $CDCl_3$.*

Synthesis of hexanoyl derivative **4**. To a mixture of the 2-acetylpyridine-4-methanol (**3**) (600 mg, 3.97 mmol) and pyridine (0.38 mL, 4.77 mmol) in anhydrous dichloromethane (20 mL) was added dropwise hexanoyl chloride (0.67 mL, 4.77 mmol) under an atmosphere of dinitrogen. This was allowed to stir at room temperature for 2 h, until analysis via TLC (SiO_2, 1% methanol in dichloromethane) showed no starting material remained. The mixture was further diluted with dichloromethane (20 mL) and washed with saturated sodium bicarbonate solution (15 mL). The combined organic layer was dried over anhydrous magnesium sulfate and solvent removed under reduced pressure, giving the crude product as a slightly pink oil. This was purified by column chromatography (SiO_2, 1% methanol in dichloromethane), affording the product (**4**) as a colourless oil (820 mg, 83%). ^{1}H NMR (400 MHz, *$CDCl_3$*) δ (ppm) = 8.68 (d, 1H, *J* = 4.9, py), 8.01(d, 1H, *J* = 0.8, py), 7.44 (dd, 1H, *J* = 5.0, 0.8, py), 5.19 (s, 2H, pyC*H_2*-), 2.74 (s, 3H, -COC*H_3*), 2.43 (t, 2H, *J* = 7.5, -COC*H_2*-), 1.68 (pent, 2H, *J* = 7.5, -$COCH_2$C*H_2*-), 1.33 (m overlap, 4H, -$COCH_2CH_2$C*H_2*C*H_2*-), 0.90 (t, 3H, *J* = 4.0 Hz, -CH_2C*H_3*). ^{13}C NMR (100 MHz, *$CDCl_3$*) δ (ppm) = 199.9 (Py*C*OCH_3), 173.3 (-CH_2*C*O_2CH_2-), 153.8 (Q), 149.3 (CH), 146.5 (Q), 125.1 (CH), 119.7 (CH), 63.8 (CH_2), 34.1 (CH_2), 31.3 (CH_2), 25.9 (-CO*C*H_3), 24.6 (CH_2), 22.3 (CH_2), 13.9 (-CH_2*C*H_3). ESI-MS *m/z* 250 (M + H^+), HR ESI-MS found 249.1367 $C_{14}H_{19}NO_3$ requires 249.1365 (error 0.84 ppm).

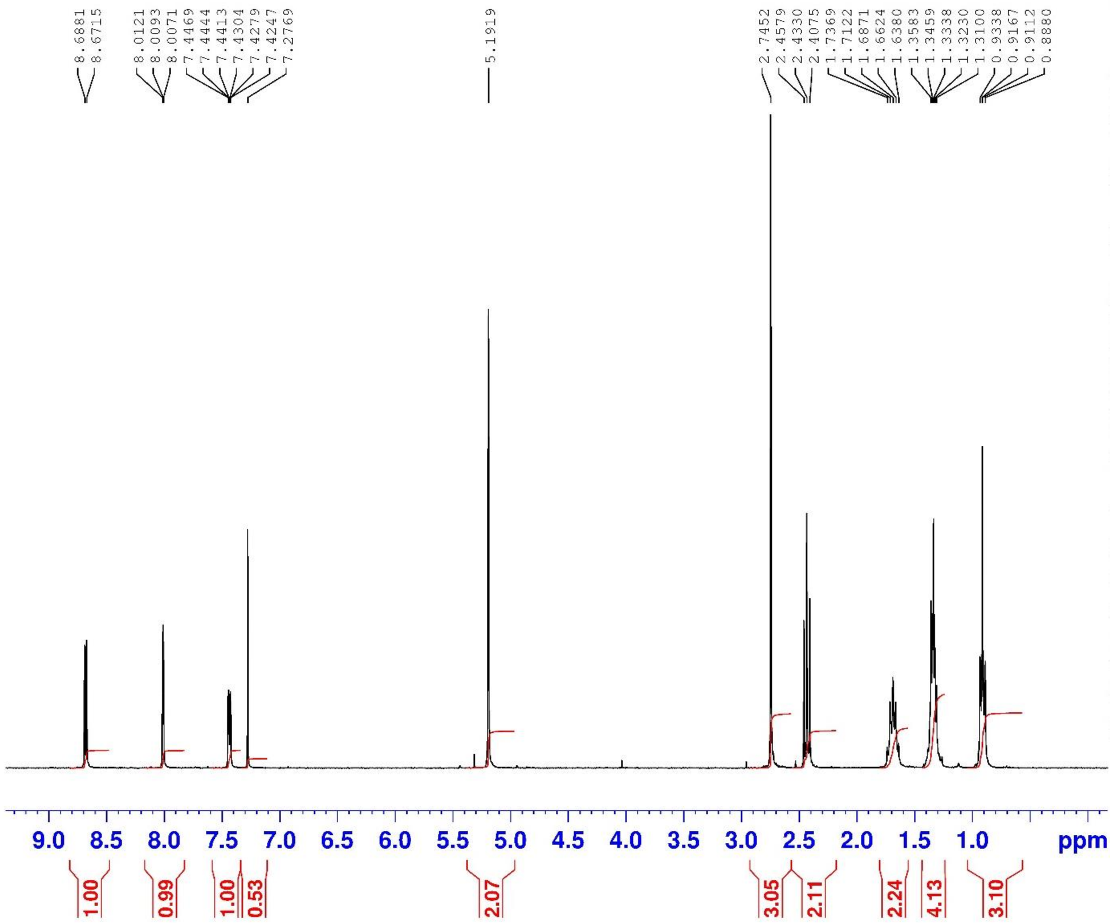

Figure 10. *^{1}H NMR of (**4**) in $CDCl_3$.*

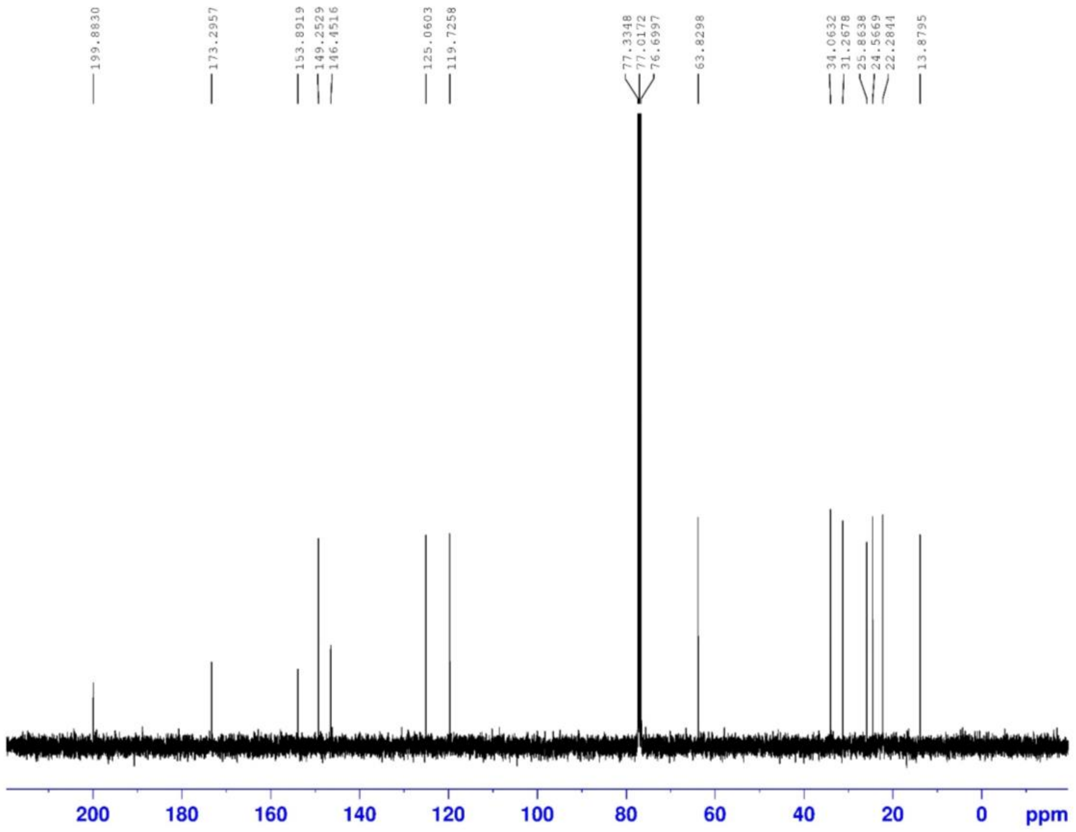

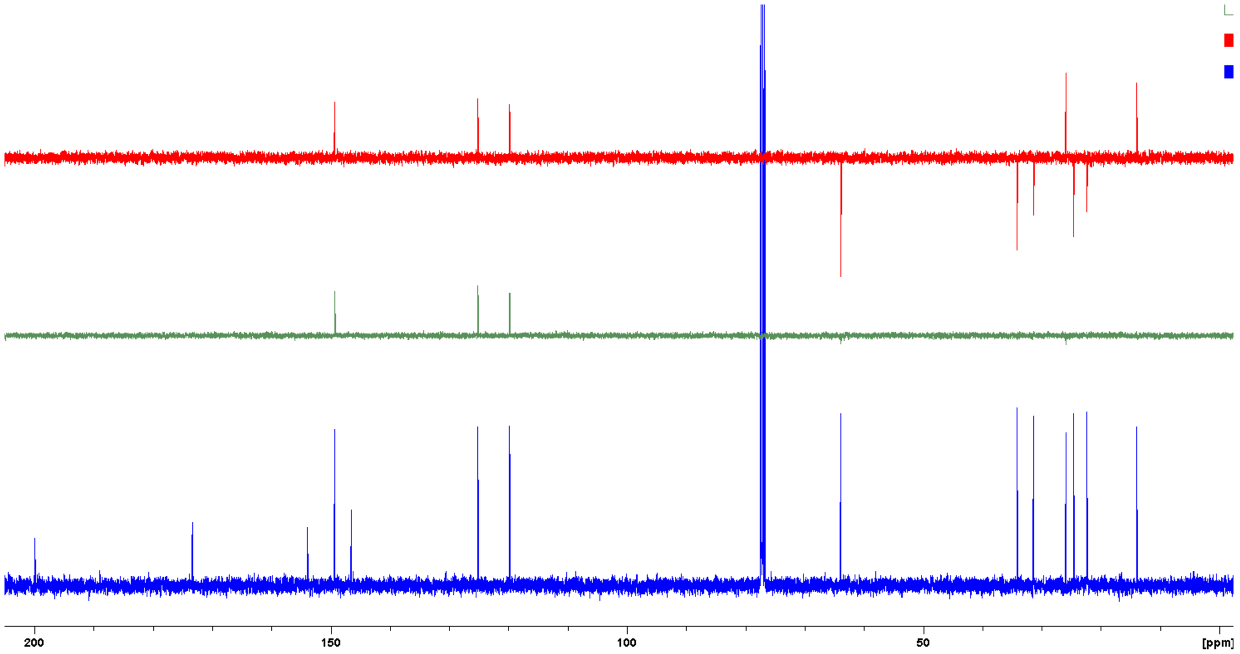

Figure 11. *C^{13} and DEPT NMR of (**4**) in $CDCl_3$.*

Synthesis of 2-(α-bromoacetyl)pyridine derivative (**5**). To a solution of bromine (462 mg, 2.89 mmol) in carbon tetrachloride (1 mL) was added dropwise to a solution of **4** (600 mg, 2.41 mmol) in carbon tetrachloride (20 mL) at 70°C. The reaction was monitored by TLC and the bromine solution added slowly until all the starting material had been consumed, during which time a yellow precipitate formed. This mixture was then allowed to cool and diluted in dichloromethane (30 mL) and washed with saturated aqueous sodium bicarbonate solution (20 mL). The organic layer was dried over anhydrous magnesium sulfate and solvent evaporated under reduced pressure to give the crude product as a slightly yellow oil. This was then purified by column chromatography (SiO_2, 1% methanol in dichloromethane) to afford the product (**5**) as a colourless oil (505 mg, 64%). ^{1}H NMR (400 MHz, *$CDCl_3$*) δ (ppm) = 8.69 (d, 1H, *J* = 5.0, Py), 8.07 (d, 1H, *J* = 0.6, Py), 7.49 (dt, 1H, *J* = 4.8, 0.7, Py), 5.22 (s, 2H, PyC*H_2*-), 4.87 (s, 2H, -PyCOC*H_2*-), 2.45 (t, 2H, *J* = 7.6, -COC*H_2*-), 1.70 (pent, 2H, *J* = 7.4, -$COCH_2$C*H_2*-), 1.35 (m overlap, 4H, -$COCH_2CH_2$C*H_2*C*H_2*-), 0.92 (t, 3H, *J* = 7.0 Hz, -CH_2C*H_3*). ^{13}C NMR (100 MHz, *$CDCl_3$*) δ (ppm) = 192.3 (Q, Py*C*OCH_2-), 173.3 (Q, CH_2*C*O_2CH_2-), 151.7 (Q), 149.4 (CH), 146.9 (Q), 125.8 (CH), 120.7 (CH), 63.7 (CH_2), 34.0 (CH_2), 32.3 (CH_2), 31.3, (CH_2) 24.6 (CH_2), 22.3 (CH_2), 13.9 (CH_3). ESI-MS *m/z* 328 (M + H^+), HR ESI-MS found 327.0475, $C_{14}H_{18}NO_3Br$ requires 327.0470 (error 1.48 ppm).

Note: other reaction solvents were tried in this experiment – chloroform gave a very poor yield and acetic acid gave almost none of the desired product.

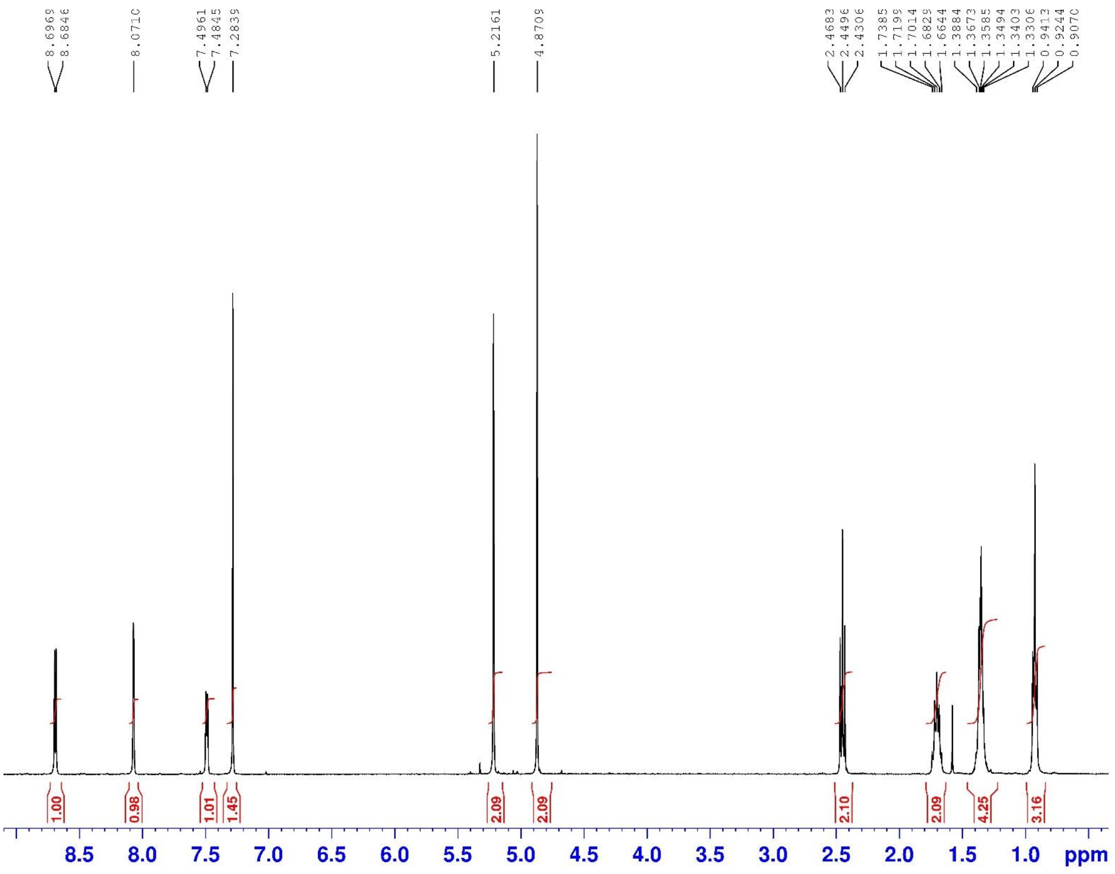

Figure 12. ^{1}*H NMR of* 2-(α-bromoacetyl)pyridine derivative (**5**) *in* $CDCl_3$ *(a =* CH_2Cl_2 *impurity).*

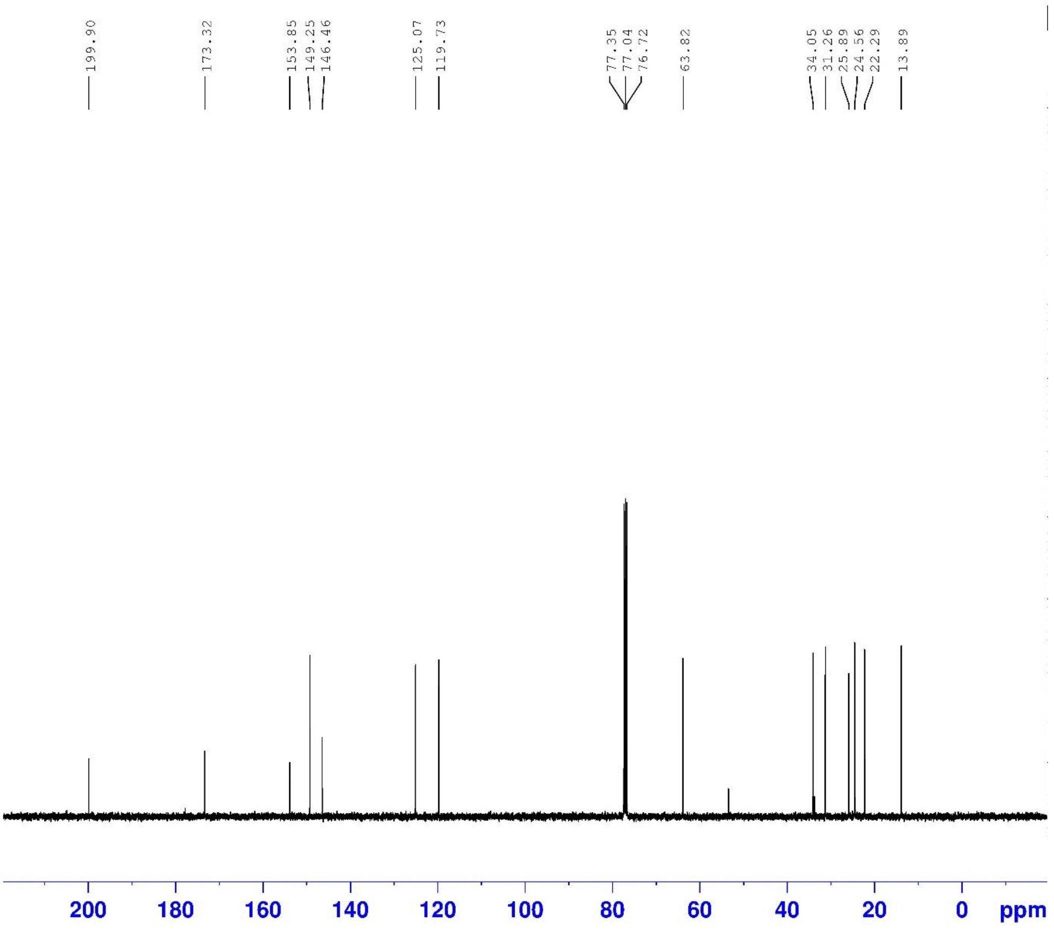

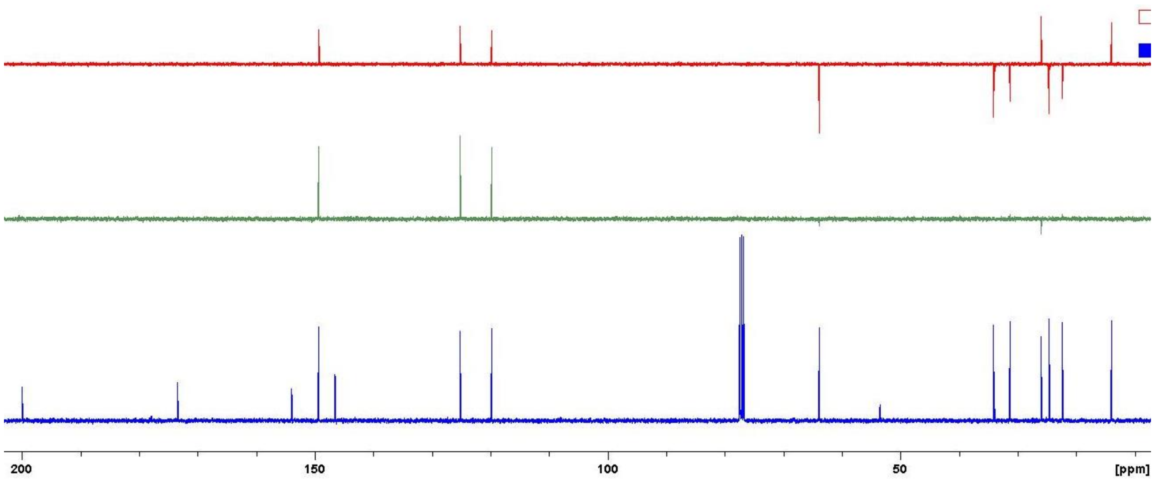

Figure 13. ^{13}C *and DEPT NMR of* 2-(α-bromoacetyl)pyridine derivative (**5**) *in CDCl₃.*

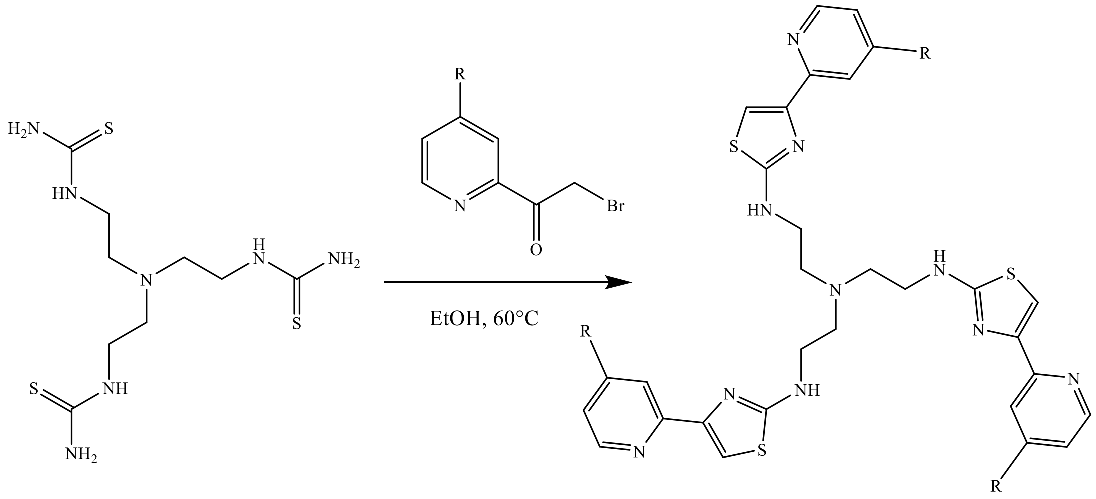

(**L**[1a])

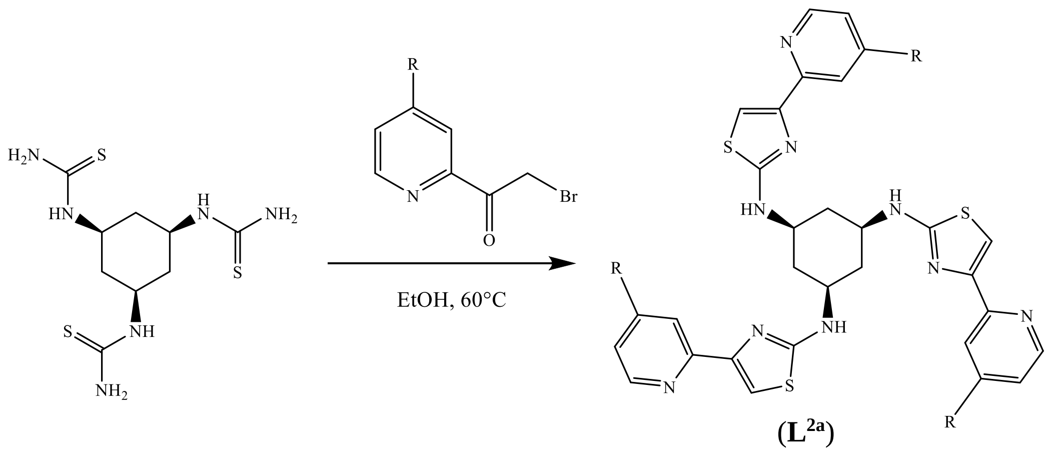

(**L**[2a])

Figure 14. Synthesis of ligands $\mathbf{L^{1a}}$ and $\mathbf{L^{2a}}$ (R = -$CH_2CO(CH_2)_4CH_3$).

Synthesis of $\mathbf{L^{1a}}$. To a solution of 2-(α-bromoacetyl)pyridine derivative (**5**) (300 mg, 0.915 mmol) in ethanol (20 mL), 1,1',1''-(nitrilotris(ethane-2,1-diyl))tris(thiourea) (90 mg, 0.278 mmol) was added. The reaction was then heated to 80°C for 12 h, during which time all the solid dissolved and the solution turned yellow. The

solution was then allowed to cool and solvent removed under reduced pressure. The resultant oil was diluted in dichloromethane (30 mL) and washed with saturated aqueous sodium bicarbonate solution (15 mL). The combined organic layers were dried over anhydrous magnesium sulfate and solvent removed under reduced pressure to give the crude product as a brown/yellow oil. This was purified by column chromatography (SiO_2, 10% methanol in dichloromethane) to afford **L^{1a}** as a light brown oil (155mg, 55%). ^{1}H NMR (300 MHz, *$CDCl_3$*) δ (ppm) = 8.55 (d, 3H, J = 5.0, py), 7.89 (s, 3H, py), 7.26 (s, 3H, tz) 7.13 (dd, 3H, J = 4.9, 0.7, py), 5.90 (s, 3H, NH), 5.14 (s, 6H, pyCH_2-), 3.44 (q, 6H, J = 5.3, NCH_2CH_2NH-), 2.90 (t, 6H, J = 5.6, NC$H_2$$CH_2$NH-), 2.40 (t, 6H, J = 7.6, -COCH_2-), 1.67 (quint, 6H, J = 7.3, -$COCH_2$CH_2-), 1.31 (m overlap, 12H, -$COCH_2CH_2$CH_2CH_2-), 0.89 (t, 9H, J = 6.5 Hz, -CH_2CH_3). ^{13}C NMR (100 MHz, *$CDCl_3$*) δ (ppm) = 173.4 (Q, -*C*O_2-), 169.5 (Q), 153.0 (Q), 150.6 (Q), 149.6 (CH), 145.9 (Q), 120.4 (CH), 119.1 (CH), 106.0 (CH), 64.3 (CH_2), 53.0 (CH_2) 43.3 (CH_2), 34.1 (CH_2), 31.3 (CH_2), 24.6 (CH_2), 22.3 (CH_2), 13.9 (CH_3, -CH_2*C*H_3). ESI-MS *m/z* 1011 (M + H$^+$), HR ESI-MS found 1010.4341 $C_{51}H_{66}N_{10}S_3O_6$ requires 1010.4329 (error 1.24 ppm).

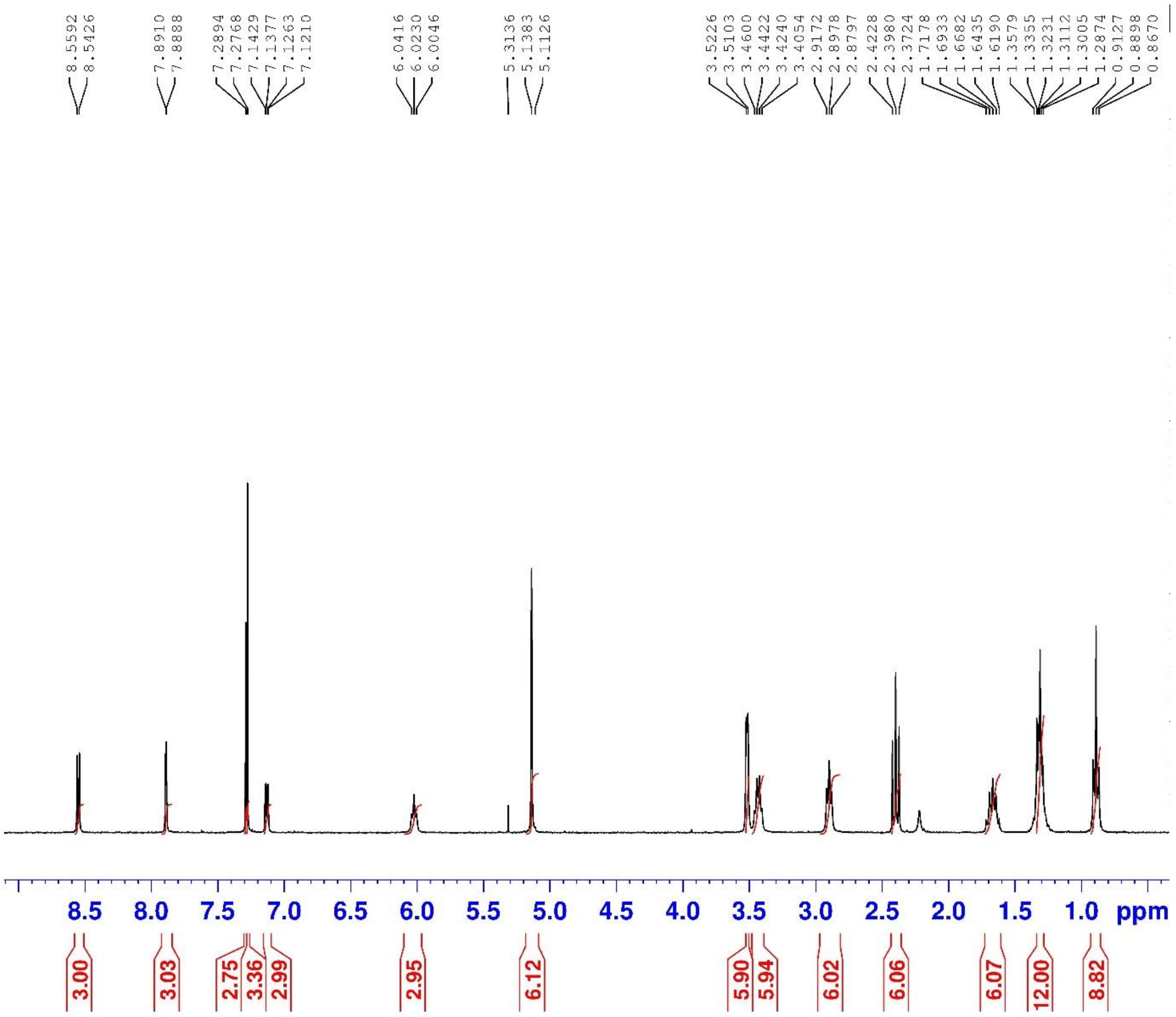

Figure 15. *^{1}H NMR of* **L^{1a}** *in $CDCl_3$ (a = CH_2Cl_2 impurity).*

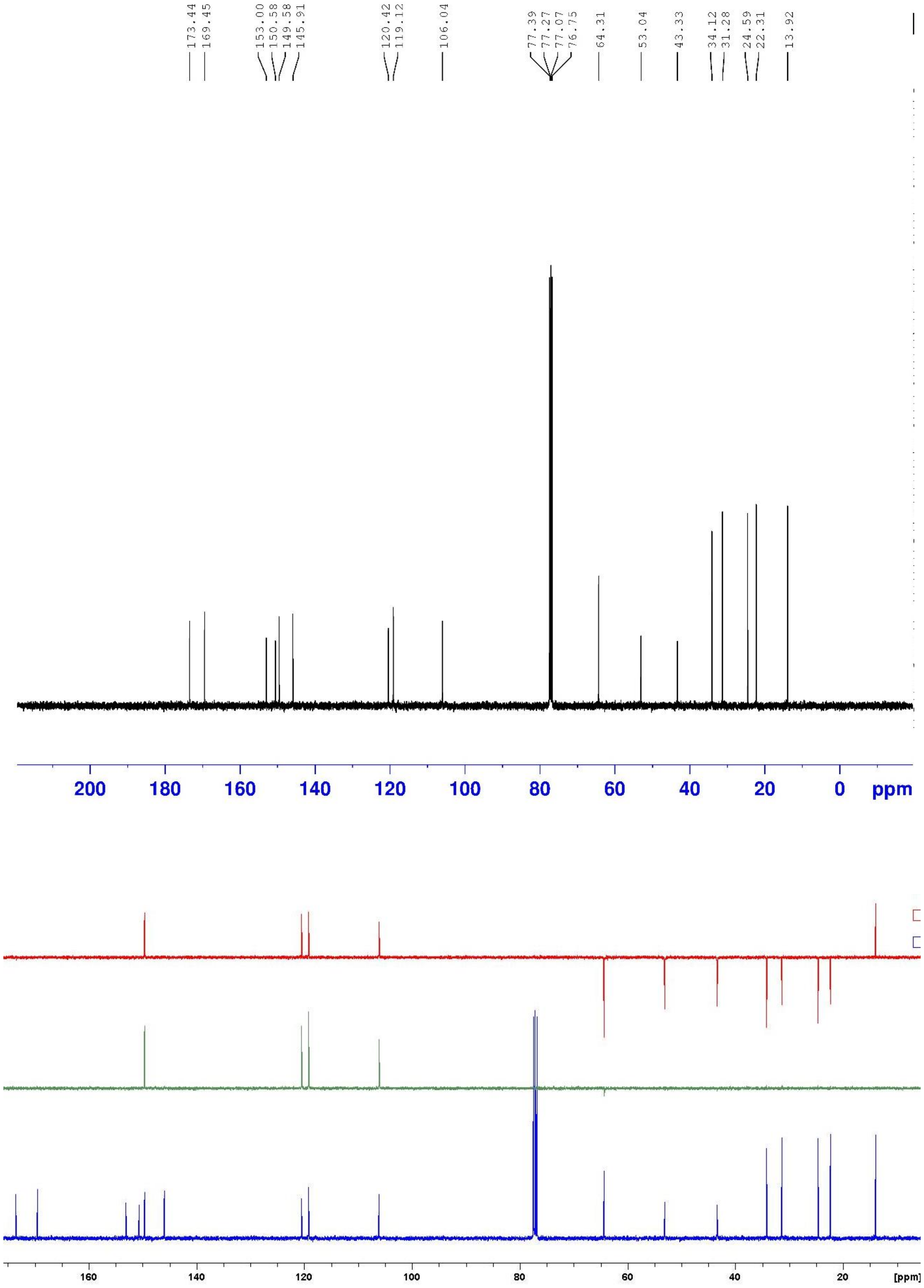

Figure 16. *^{13}C and DEPT NMR of* **L^{1a}** *in CDCl$_3$.*

Synthesis of $\mathbf{L^{2a}}$. To a solution of 2-(α-bromoacetyl)pyridine derivative (**5**) (373 mg, 1.13 mmol) in ethanol (20 mL), cis,cis-cyclohexyl-1,3,5-trithiourea (**2**) (105 mg, 0.343 mmol) was added. The reaction was heated to 80°C for 12 h, during which time all the solid dissolved and the solution turned yellow. The solution was then allowed to cool and solvent removed under reduced pressure. The resultant oil was diluted in dichloromethane (40 mL) and washed with saturated aqueous sodium bicarbonate solution (20 mL). The combined organic layers were dried over anhydrous magnesium sulfate and solvent removed under reduced pressure to give the crude product as a yellow solid. This was purified by column chromatography (SiO_2, 10% methanol in dichloromethane) to afford pure $\mathbf{L^{2a}}$ as a dark yellow solid (220 mg, 64%). ^{1}H NMR (400 MHz, *CDCl₃*) δ (ppm) = 8.47 (d, 3H, J = 5.0, py), 7.80 (s, 3H, py), 7.25 (s, 3H, tz), 7.06 (dd, 3H, J = 5.0, 1.1, py), 5.61 (d, 3H, J = 7.4, NH), 5.07 (s, 6H, py-CH_2-), 3.65 (m, 3H, cy-CH), 2.64 (d, 3H, J = 11.4, cy-CH), 2.32 (t, 6H, J = 7.5, COCH_2), 1.58 (quint, 6H, COCH$_2$CH_2-), 1.21 (m overlap, 12H, -COCH$_2$CH$_2$CH_2CH_2-), 1.00 (q, 3H, J = 11.8, cy-CH), 0.79 (t, 9H, J = 6.9 Hz, -CH$_2$CH_3). ^{13}C NMR (100 MHz, *CDCl₃*) δ (ppm) = 173.49 (Q, -CO$_2$-), 168.04 (Q), 153.0 (Q), 150.8 (Q), 149.7 (CH), 146.1 (Q), 120.4 (CH), 119.1 (CH), 106.1 (CH), 64.3 (CH$_2$), 51.5 (CH), 38.5 (CH$_2$), 34.1 (CH$_2$), 31.3 (CH$_2$), 24.6 (CH$_2$), 22.3 (CH$_2$), 13.9 (CH$_3$). ESI-MS *m/z* 994 (M + H$^+$), HR ESI-MS found 993.4077, $C_{51}H_{63}N_9S_3O_6$ requires 993.4063 (error 1.34 ppm).

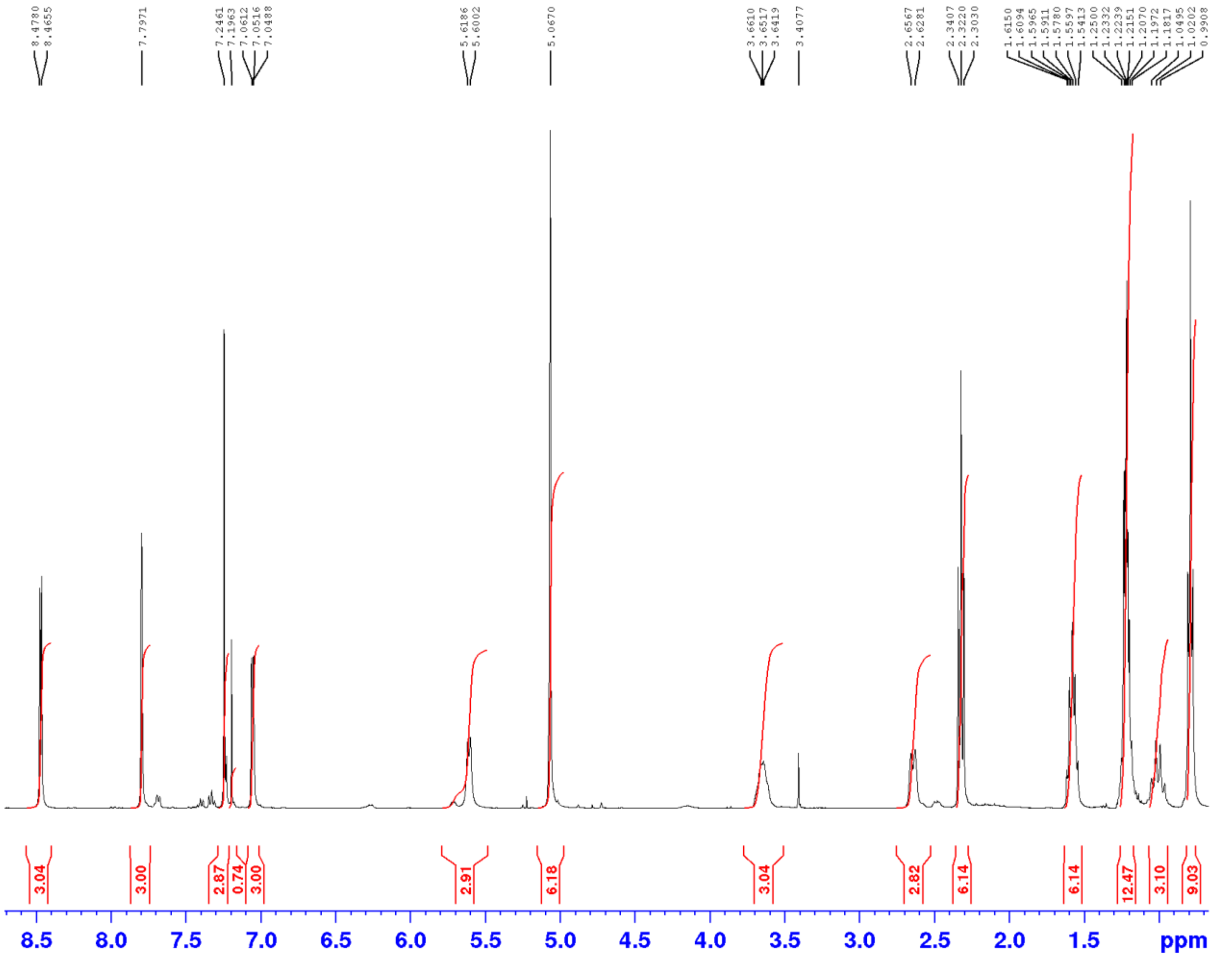

Figure 17. *^{1}H NMR of* **L^{2a}** *in $CDCl_3$.*

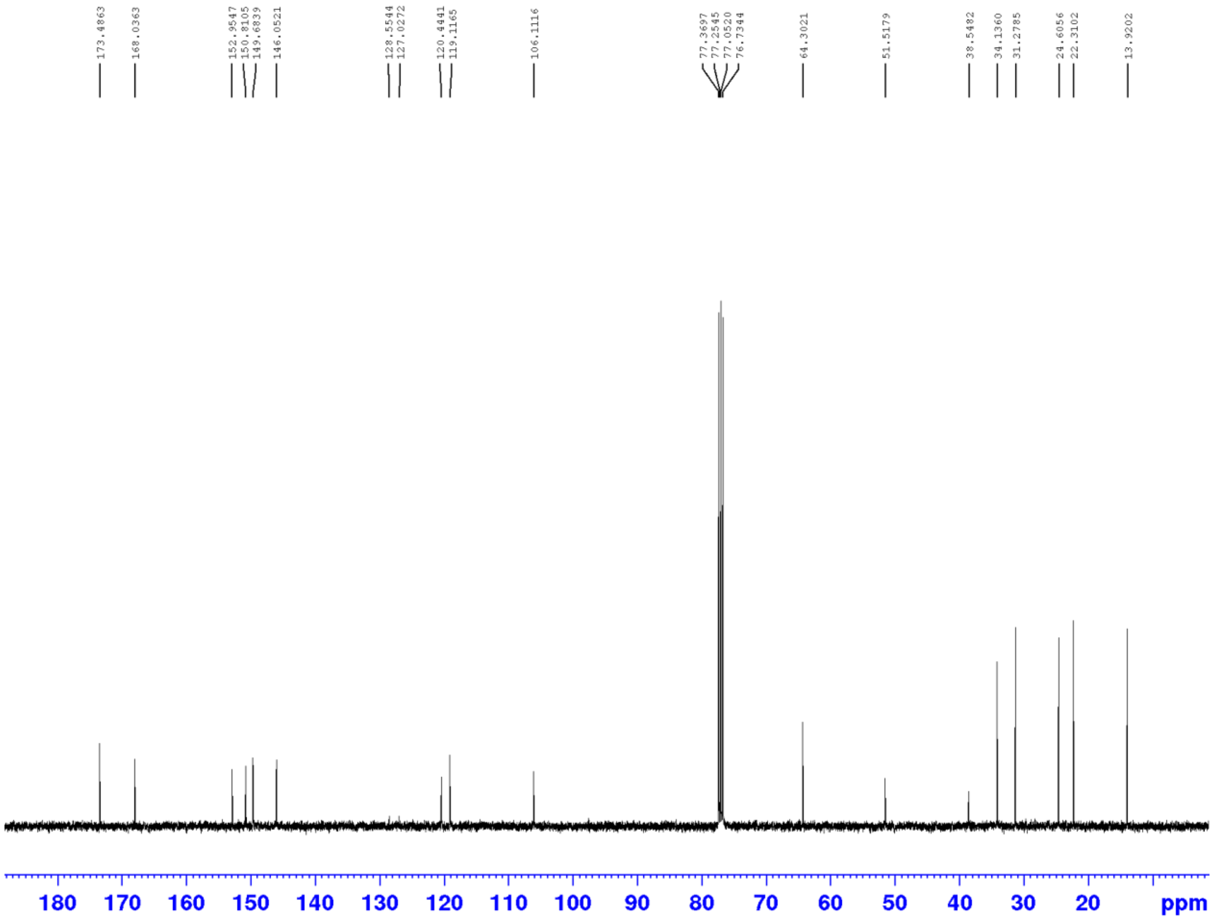

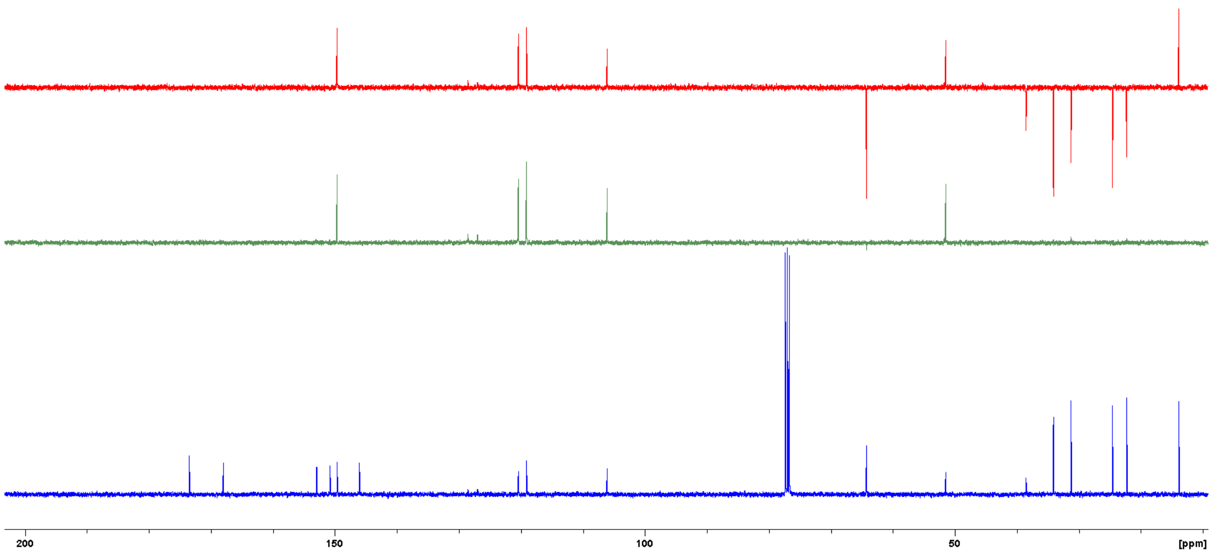

Figure 18. *^{13}C and DEPT NMR of* **L^{1a}** *in $CDCl_3$.*

Crystals structure of [(L^1)$_2$Cu$_3$(Br)]$^{5+}$.

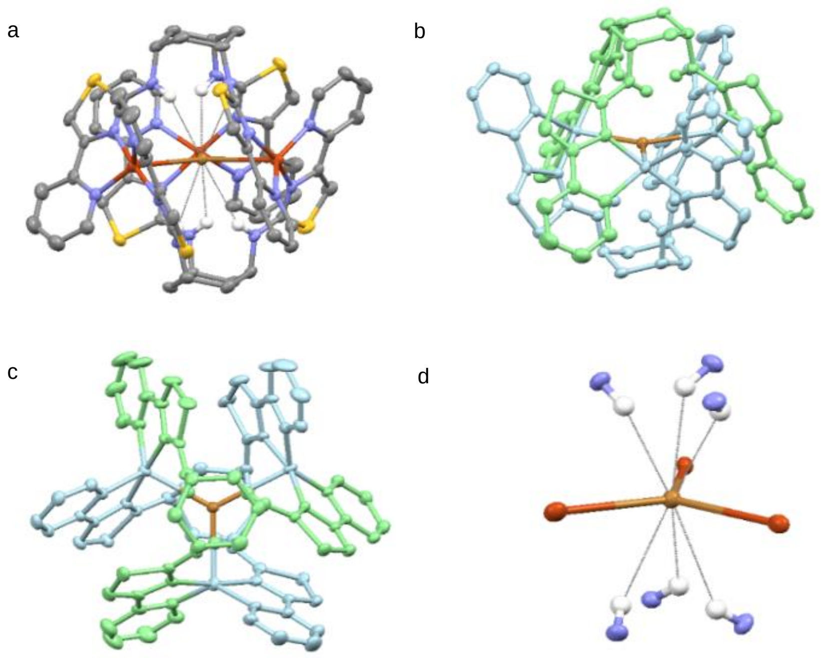

Figure 19. (a) – (d) Single-crystal X-ray structure of [(**L^1**)$_2$Cu$_3$(Br)]$^{5+}$ showing the encapsulation and hydrogen bonding of the anion. Thermal ellipsoids are shown at the 50% probability level. Selected hydrogen atoms and anions are omitted for clarity. Colour code: orange, Cu^{2+}; red, O; blue, N; yellow, S; grey, C; brown Br (apart from 2b and 2c where the ligands have been coloured for clarity). Comparison of this species with [(**L^1**)$_2$Cu$_3$Br]$^{5+}$ shows many similarities, but there are closer interactions been the anion and both the Cu^{2+} (Cu····Br range 2.762 – 3.092 Å for [(**L^2**)$_2$Cu$_3$Br]$^{5+}$ *vs* 2.884 – 3.413 Å for [(**L^1**)$_2$Cu$_3$Br]$^{5+}$) and amine hydrogen atoms (–NH····Br range 2.832 – 2.945 Å for [(**L^2**)$_2$Cu$_3$Br]$^{5+}$ *vs* 2.869 – 3.156 Å for [(**L^1**)$_2$Cu$_3$Br]$^{5+}$). With the reduction in distances again attributable to the smaller cyclohexyl spacer unit. The host-guest complex is also observed in the gas phase with ions in the ESI-MS at *m/z* 2085 and 968 corresponding to {[(**L^2**)$_2$Cu$_3$Br](trif)$_4$}$^+$ and {[(**L^2**)$_2$Cu$_3$Br](trif)$_3$}$^{2+}$ respectively.

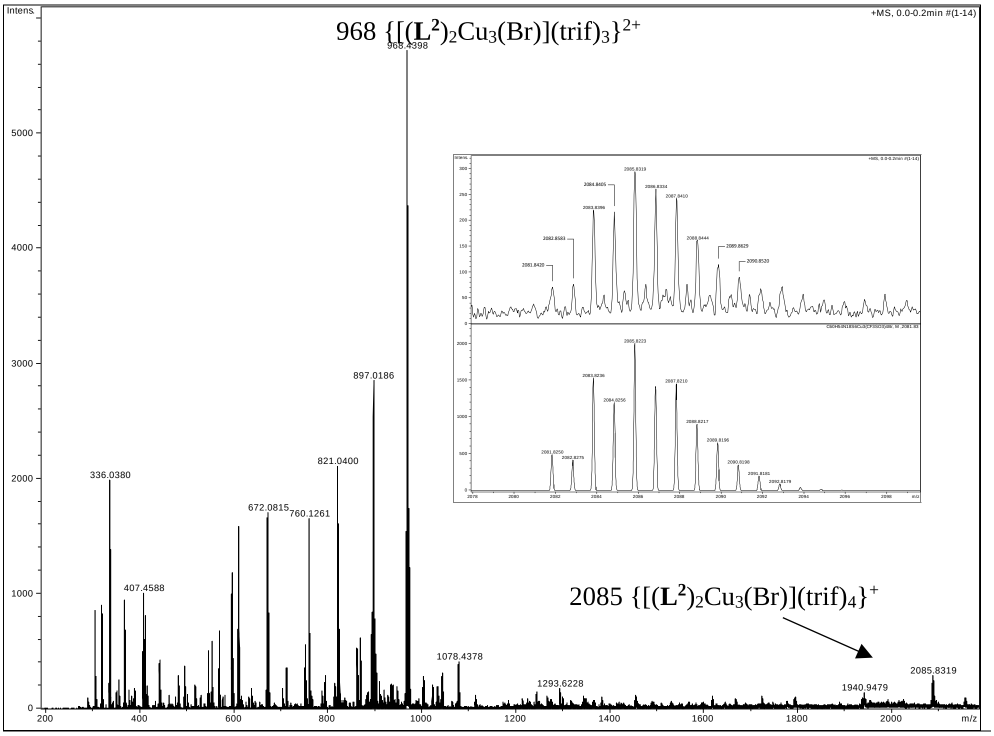

Figure 20. ESI-MS of $[(\mathbf{L}^2)_2Cu_3(Br)](trif)_5$ showing ions at *m/z* 2085 and 968 corresponding to $\{[(\mathbf{L}^2)_2Cu_3(Br)](trif)_4\}^+$ and $\{[(\mathbf{L}^2)_2Cu_3(Br)](trif)_3\}^{2+}$ for each ligand respectively**.** The isotope pattern for the ion at *m/z* 2085 is shown inset (top observed and bottom calculated).

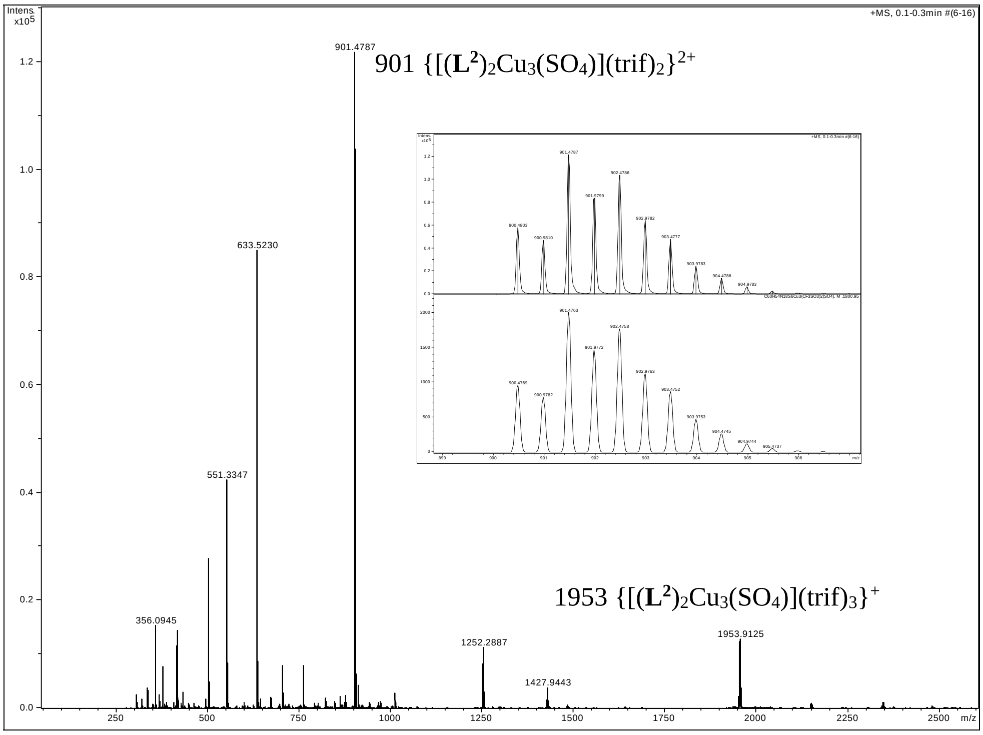

Figure 21. ESI-MS of $[(\mathbf{L}^2)_2Cu_3(SO_4)](trif)_4$ showing ions at *m/z* 1954 and 901 corresponding to $\{[(\mathbf{L}^2)_2Cu_3(SO_4)](trif)_3\}^+$ and $\{[(\mathbf{L}^2)_2Cu_3(SO_4)](trif)_2\}^{2+}$ for each ligand respectively**.** The isotope pattern for the ion at *m/z* 901 is shown inset (top observed and bottom calculated).

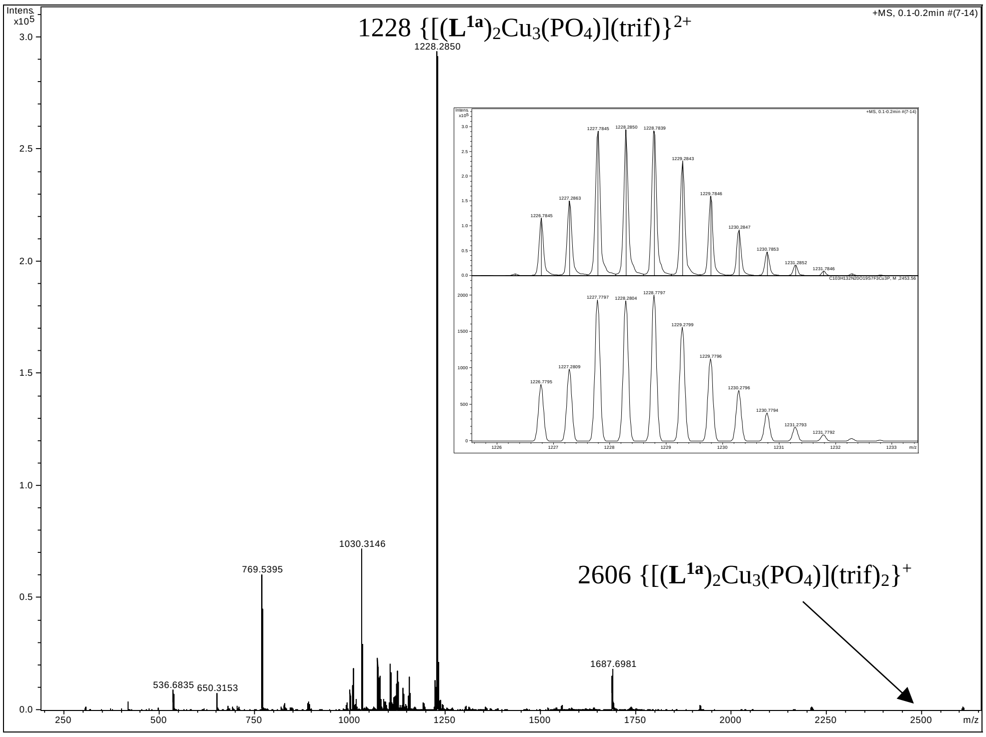

Figure 22. ESI-MS of $[(\mathbf{L^{1a}})_2Cu_3(PO_4)](trif)_3$ showing ions at *m/z* 2606 and 1228 corresponding to $\{[(\mathbf{L^{1a}})_2Cu_3(PO_4)](trif)_2\}^+$ and $\{[(\mathbf{L^{1a}})_2Cu_3(PO_4)](trif)\}^{2+}$ for each ligand respectively**.** The isotope pattern for the ion at *m/z* 1228 is shown inset (top observed and bottom calculated). The ion at *m/z* 2606 is small but reproducible.

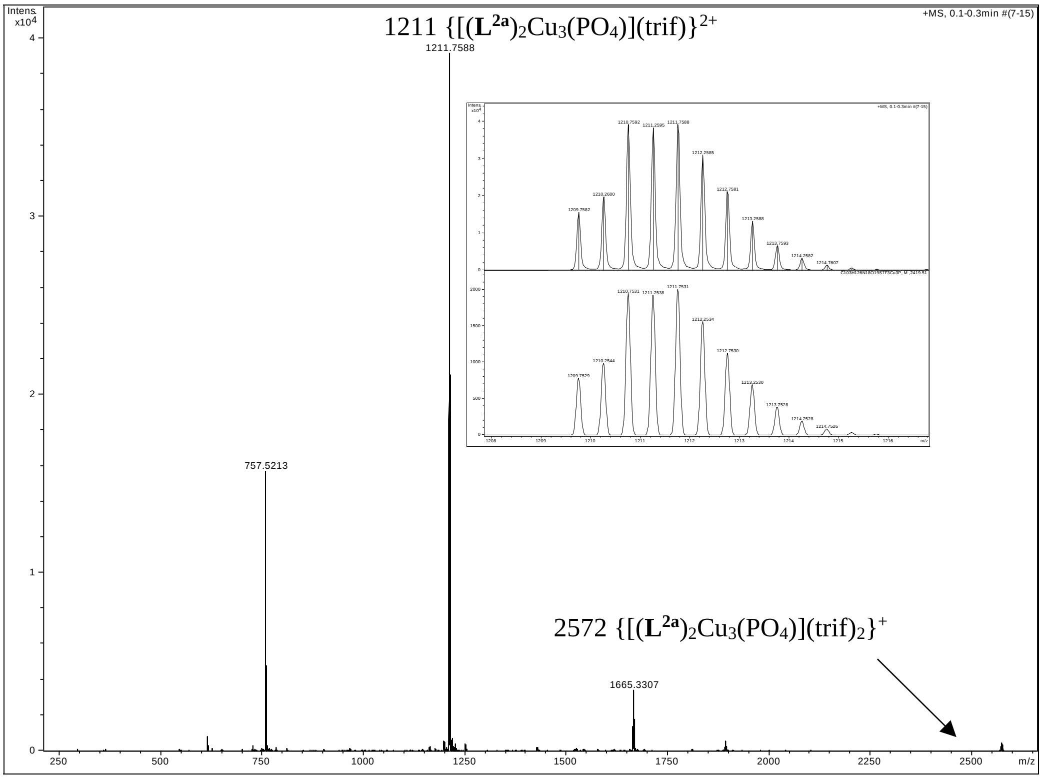

Figure 23. ESI-MS of $[(\mathbf{L^{2a}})_2Cu_3(PO_4)](trif)_3$ showing ions at *m/z* 2572 and 1221 corresponding to $\{[(\mathbf{L^{2a}})_2Cu_3(PO_4)](trif)_2\}^+$ and $\{[(\mathbf{L^{2a}})_2Cu_3(PO_4)](trif)\}^{2+}$ for each ligand respectively**.** The isotope pattern for the ion at *m/z* 1222 is shown inset (top observed and bottom calculated). The ion at *m/z* 2572 is small but reproducible.

UV-Vis studies.

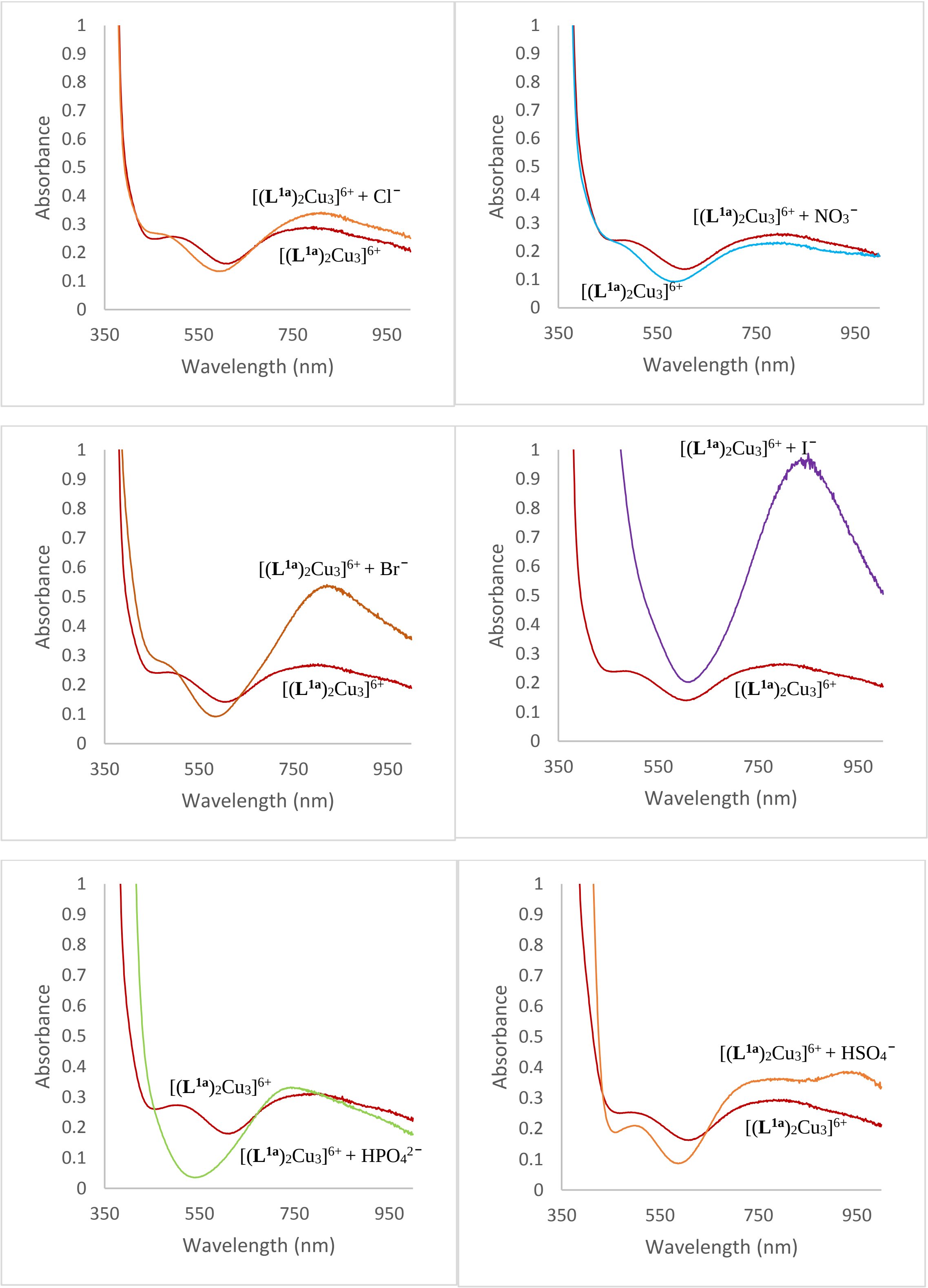

Figure 24. UV-Vis absorption spectra of a solution of [(**L**^{1a})$_2$Cu$_3$](trif)$_6$ in 3% MeOH in DCM plus the addition of one equivalent of various anions (as either their tetraethyl- or tetrabutyl-ammonium salts). [(**L**^{1a})$_2$Cu$_3$](trif)$_6$ 0.8 mM.

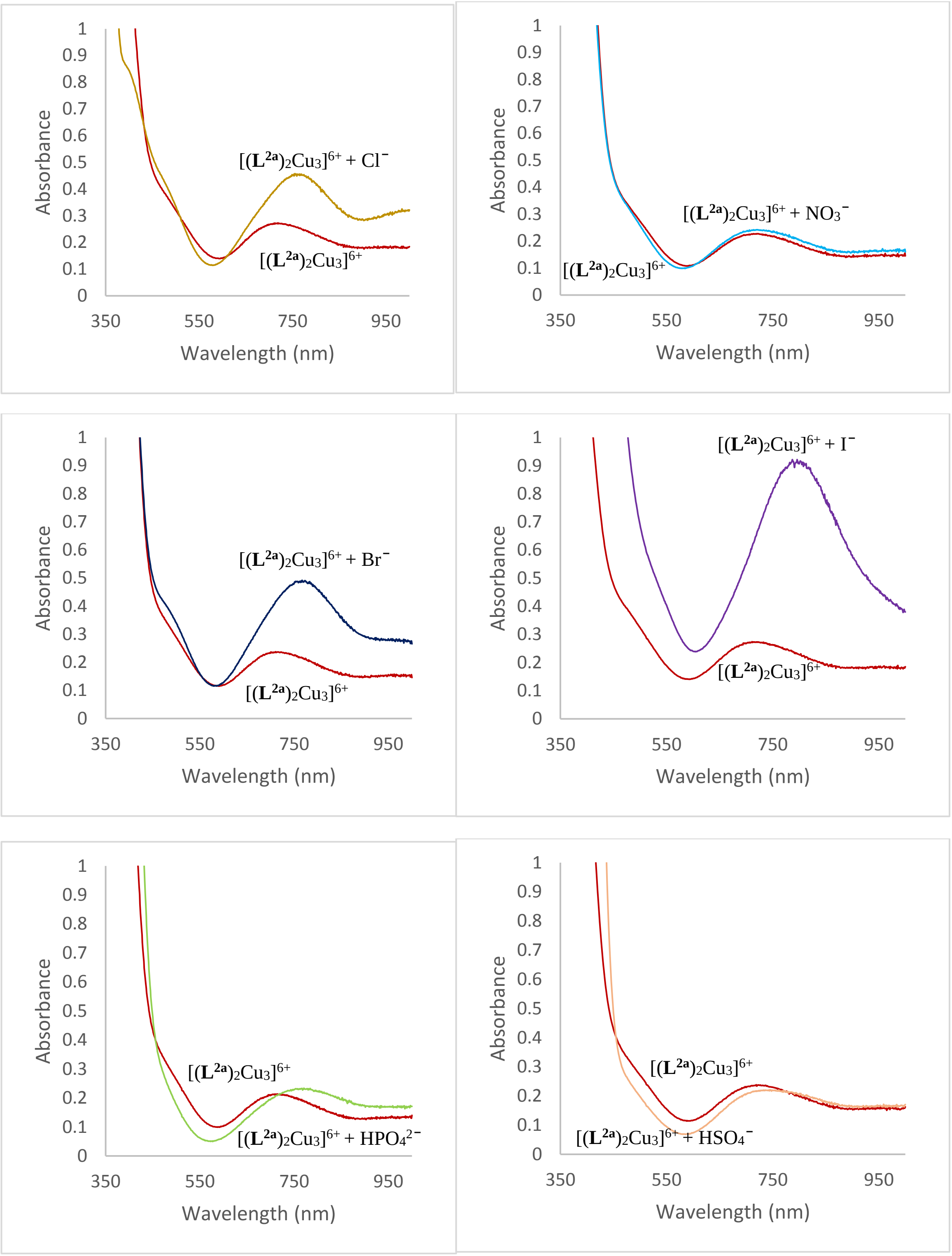

Figure 25. UV-Vis absorption spectra of a solution of $[(\mathbf{L^{2a}})_2Cu_3](trif)_6$ in 3% MeOH in DCM plus the addition of one equivalent of various anions (as either their tetraethyl- or tetrabutyl-ammonium salts). $[(\mathbf{L^{2a}})_2Cu_3](trif)_6$ 0.8 mM.

S1. D. Ben-Ishai and A. Berger, *J. Org. Chem.*, 1952, **17**, 1564 – 1550.

S2. C. Mikel and P. Potvin, *Polyhedron*, 2002, 21, 49 – 54; A. Jacques, S. Cerfontaine and B. Elias, *J. Org. Chem.*, 2015, **80,** 11143 – 11148.

S3. C. R. Rice, C. Slater, R. A. Faulkner, R. L. Allan, *Angew. Chem.* 2018, **57**, 13071-13075.

Ion Chromatography Experiments.

Calibration standards of the concentrations 0.2, 0.4, 0.6, 0.8 and 1.0 mM were prepared by the standard dilution of a 20 mM stock of each mixed salt solution used. These are detailed below:

Calibration for Experiment 1. Competitive extraction of common anions (NaCl, $NaNO_3$, $NaHSO_4$ and NaH_2PO_4).

$NaHSO_4 \cdot H_2O$ (138.0 mg, 1.0 mmol), NaH_2PO_4 (120.0 mg, 1.0 mmol), NaCl (58.5 mg, 1.0 mmol) and $NaNO_3$ (85.0 mg, 1.0 mmol) in ultrapure H_2O (50 mL, 18.2 MΩ-cm).

Calibration for Experiment 2. Competitive extraction of common anions (NaCl, $NaNO_3$, Na_2SO_4 and Na_2HPO_4) and total phosphate concentration experiments.

$Na_2SO_4 \cdot 10H_2O$ (322.2 mg, 1.0 mmol), $Na_2HPO_4 \cdot 2H_2O$ (178.0 mg, 1.0 mmol), NaCl (58.5 mg, 1.0 mmol) and $NaNO_3$ (85.0 mg, 1.0 mmol) in ultrapure H_2O (50 mL, 18.2 MΩ-cm).

Experiment 1. Competitive extraction of common anions (NaCl, $NaNO_3$, $NaHSO_4$ and NaH_2PO_4).

In a typical experiment; To a solution of $\mathbf{L^{1a}}$ (10 mg, 0.010 mmol) and $Cu(trif)_2$ (5.37 mg, 0.015 mmol) in 3% MeOH in DCM (3 mL) and ultrapure H_2O (2 mL, 18.2 MΩ-cm) was added 1 mL of a mixed salts solution consisting of $NaHSO_4 \cdot H_2O$ (68.3 mg, 0.495 mmol), NaH_2PO_4 (59.4 mg, 0.495 mmol), NaCl (29.0 mg, 0.495 mmol) and $NaNO_3$ (42.1 mg, 0.495 mmol) in ultrapure H_2O (100 mL, 18.2 MΩ-cm) and this was set to stir at RT for 18 hours. After this time, 2 mL of the aqueous layer was taken and adjusted volumetrically to 5 mL with ultrapure H_2O for analysis by IC (theoretical maximum concentration of each anion 0.66mM). The remaining experiments were carried out in an identical manner but using different mixed salts solutions and the details of these are below.

Experiment 2. Competitive extraction of common anions (NaCl, $NaNO_3$, Na_2SO_4 and Na_2HPO_4).

$Na_2SO_4 \cdot 10H_2O$ (159.4 mg, 0.495 mmol), $Na_2HPO_4 \cdot 2H_2O$ (88.1 mg, 0.495 mmol), NaCl (29.0 mg, 0.495 mmol) and $NaNO_3$ (42.1 mg, 0.495 mmol) in ultrapure H_2O (100 mL, 18.2 MΩ-cm).

Experiment 3. Total NaH_2PO_4 concentration.

In a typical experiment; To a solution of $\mathbf{L^{1a}}$ (10 mg, 0.010 mmol) and $Cu(trif)_2$ (5.37 mg, 0.015 mmol) in 3% MeOH in DCM (3 mL) was added 1 mL of a solution containing $Na_2HPO_4 \cdot 2H_2O$ (88.1 mg, 0.5 mmol) in ultrapure H_2O (100 mL), ultrapure H_2O (2 mL, 18.2 MΩ-cm) and this was set to stir at RT for 18 hours. 2 mL of the aqueous layer was then taken and adjusted volumetrically to 5 mL with ultrapure H_2O for analysis by IC (theoretical maximum concentration of phosphate = 0.66mM).

Further experiments (experiments **4 - 8**) were carried out in an identical manner but using differing amounts of $\mathbf{L^{1a}}$ and $\mathbf{L^{2a}}$ and the resulting change in the stoichiometric amount of $Cu(trif)_2$ to form the complex and the details of these are tabulated below.

Experiment no.	Ligand (**L**)	Mass of **L** (mg)	Mass (mg) of $Cu(Trif)_2$ (mg)
**4**	$\mathbf{L^{1a}}$	11.0	5.91
**5**	$\mathbf{L^{1a}}$	12.0	6.44
**6**	$\mathbf{L^{2a}}$	10.0	5.38
**7**	$\mathbf{L^{2a}}$	11.0	5.92
**8**	$\mathbf{L^{2a}}$	12.0	6.46

Table 1. The masses of ligand and stoichiometric amounts of $Cu(trif)_2$ used in experiments **4** – **8** detailed below.

Ion Chromatography. Conditions and instrumentation:

IC		
Instrument	Metrohm 850 IC system	
Column	Metrohm A Supp 5 column (150 mm x 4.0 mm)	
Oven (^{o}C)	30°C	
Pump	Mobile Phase A	3.2 mM Sodium Carbonate / 1.0 mM Sodium Bicarbonate
	Mobile Phase B	N.A.
	Flow (mls/min)	0.70
	Isocratic/Gradient	Isocratic
	Runtime (mins)	20
Injector	Volume (µl)	10
Suppressor	Regenerant Solution	0.1 M Oxalic acid / 0.1 M Sulfuric acid in 10% acetone
Detector	Conductance / PAD	Conductance

<u>Experiment 1</u> <u>(calibration curves):</u>

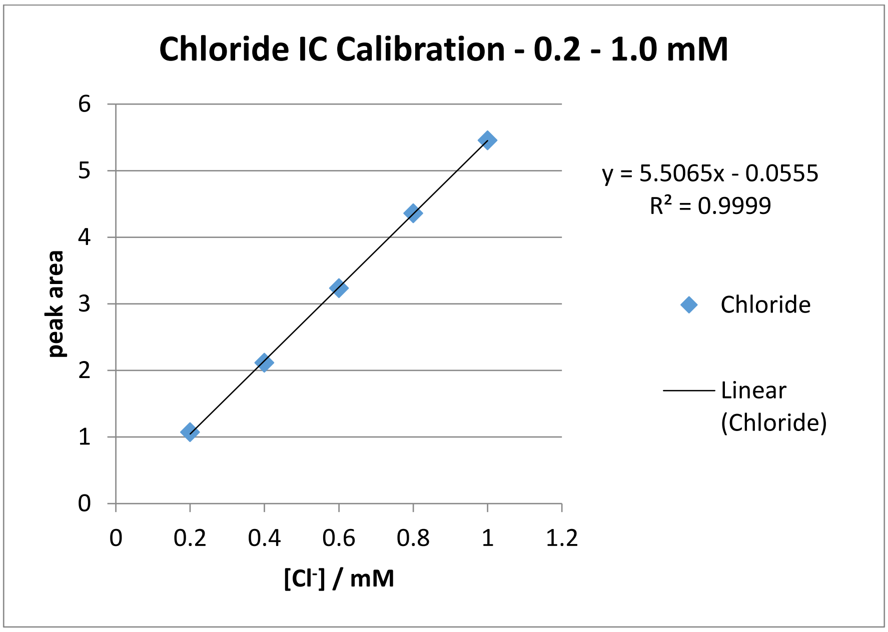

Figure IC-1. IC calibration curve of 0.2 – 1.0 mM solutions of NaCl for experiment **1**.

$[NO_3^-]$ / mM	Peak area
0.2	1.034
0.4	2.046
0.6	3.143
0.8	4.236
1	5.296

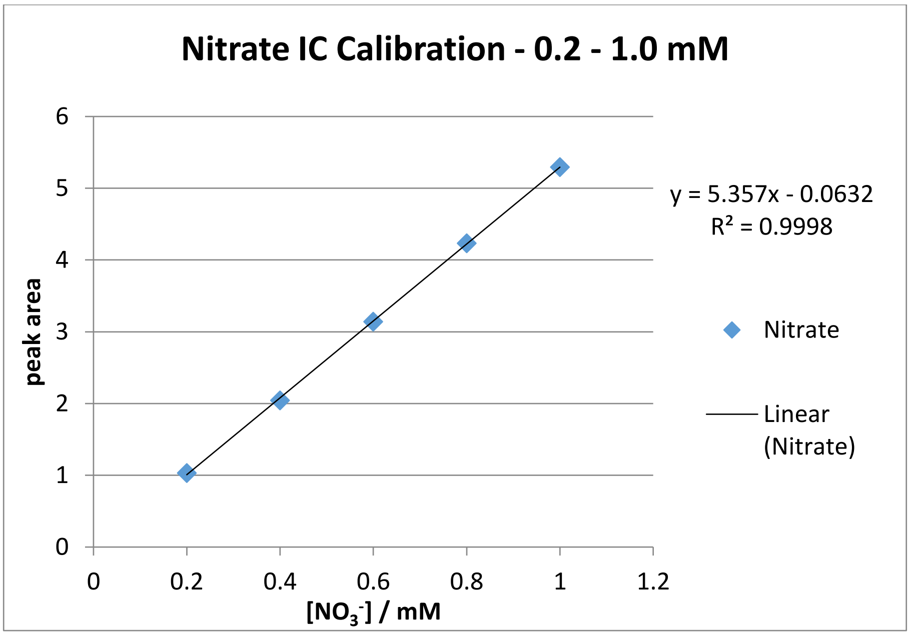

Figure IC-2. IC calibration curve of 0.2 – 1.0 mM solutions of $NaNO_3$ for experiment **1**.

$[PO_4^{3-}]$ / mM	Peak area
0.2	0.849
0.4	1.723
0.6	2.676
0.8	3.624
1	4.569

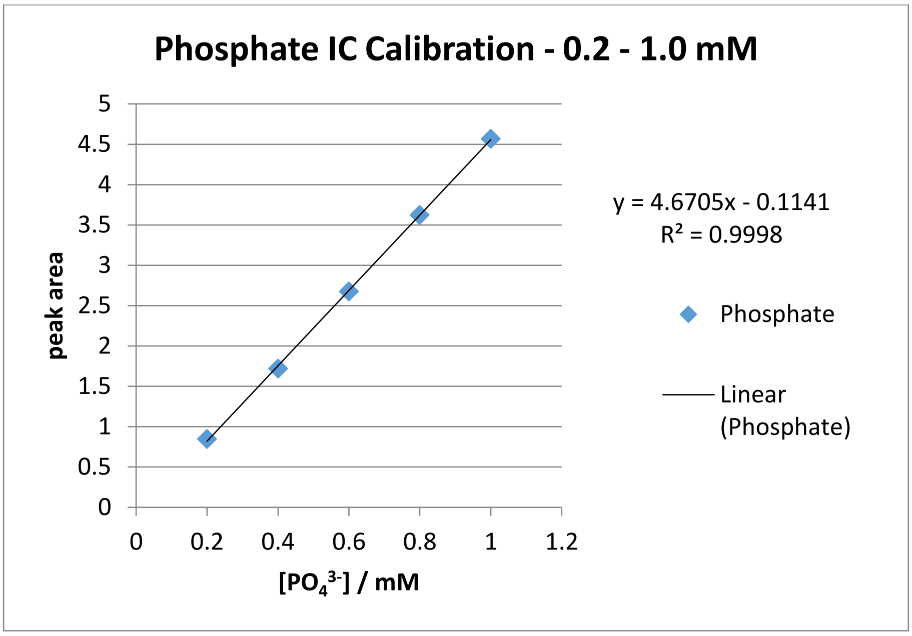

Figure IC-3. IC calibration curve of 0.2 – 1.0 mM solutions of NaH_2PO_4 for experiment **1**.

$[SO_4^{2-}]$ / mM	Peak area
0.2	2.184
0.4	4.35
0.6	6.645
0.8	8.93
1	11.161

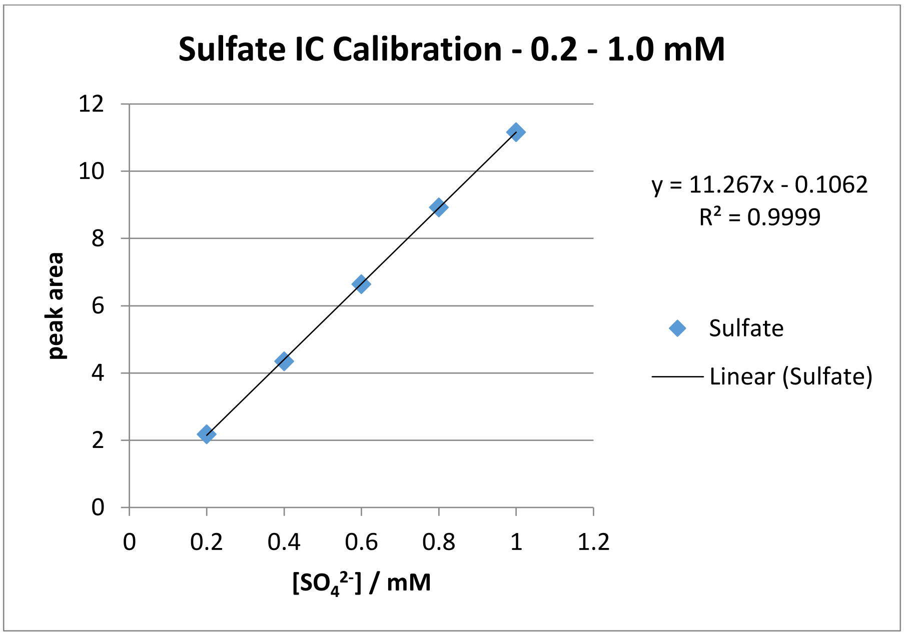

Figure IC-4. IC calibration curve of 0.2 – 1.0 mM solutions of $NaHSO_4 \cdot H_2O$ for experiment **1**.

Experiment 2 (calibration curves):

$[Cl^-]$ / mM	Peak area
0.2	1.07
0.4	2.122
0.6	3.232
0.8	4.332
1	5.436

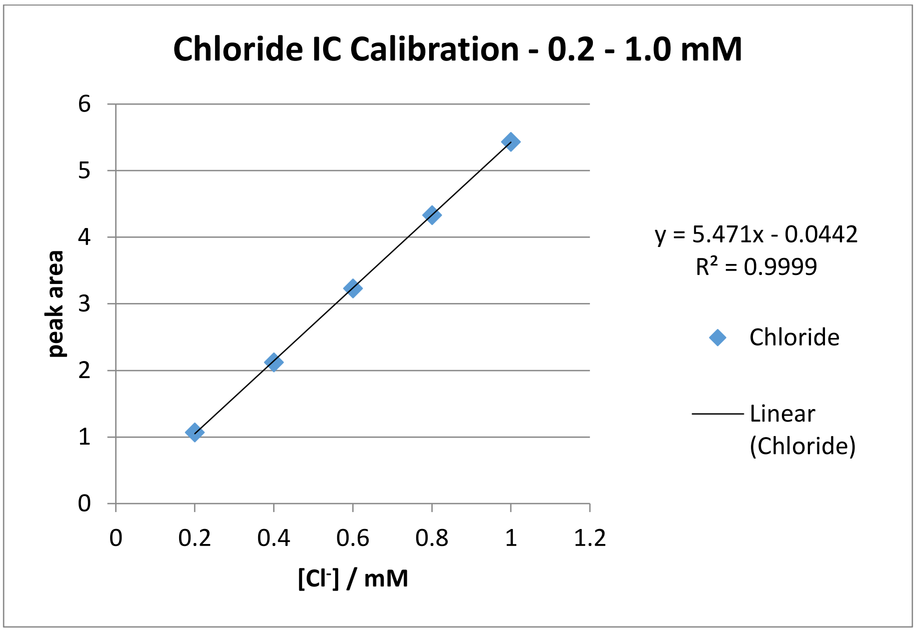

Figure IC-5. IC calibration curve of 0.2 – 1.0 mM solutions of NaCl for experiment **2**.

$[NO_3^-]$ / mM	Peak area
0.2	1.033
0.4	2.05
0.6	3.138
0.8	4.208
1	5.282

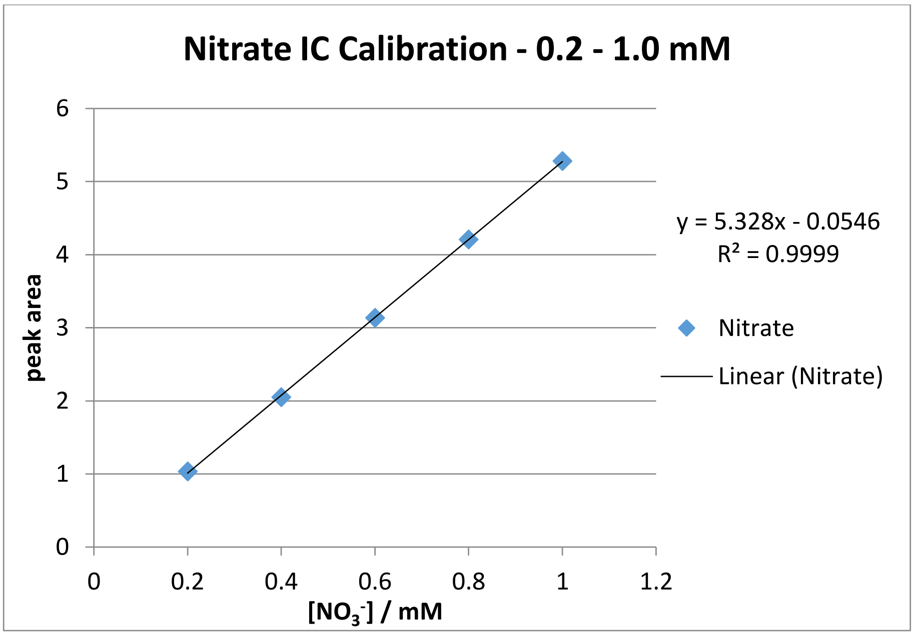

Figure IC-6. IC calibration curve of 0.2 – 1.0 mM solutions of $NaNO_3$ for experiment **2**.

$[PO_4^{3-}]$ / mM	Peak area
0.2	0.855
0.4	1.727
0.6	2.658
0.8	3.603
1	4.545

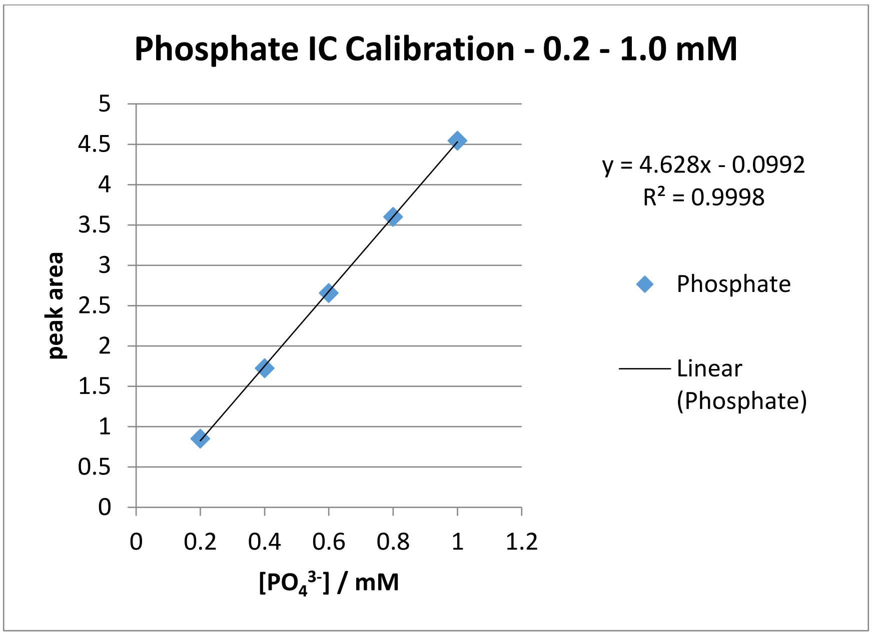

Figure IC-7. IC calibration curve of 0.2 – 1.0 mM solutions of $Na_2HPO_4·2H_2O$ for experiment **2**.

$[SO_4^{2-}]$ / mM	Peak area
0.2	2.189
0.4	4.337
0.6	6.598
0.8	8.891
1	11.091

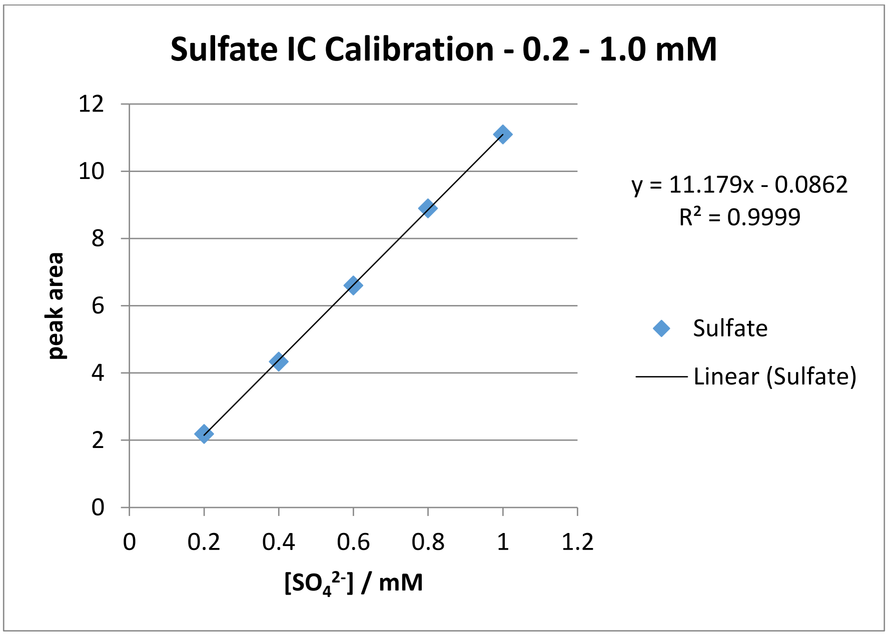

Figure IC-8. IC calibration curve of 0.2 – 1.0 mM solutions of $Na_2SO_4·10H_2O$ for experiment **2**.

Experiment 3 – 8 (calibration curves)

$[Na_2HPO_4]$ / mM	Peak area
0.2	0.88
0.4	1.84
0.6	2.84
0.8	3.874
1	4.875

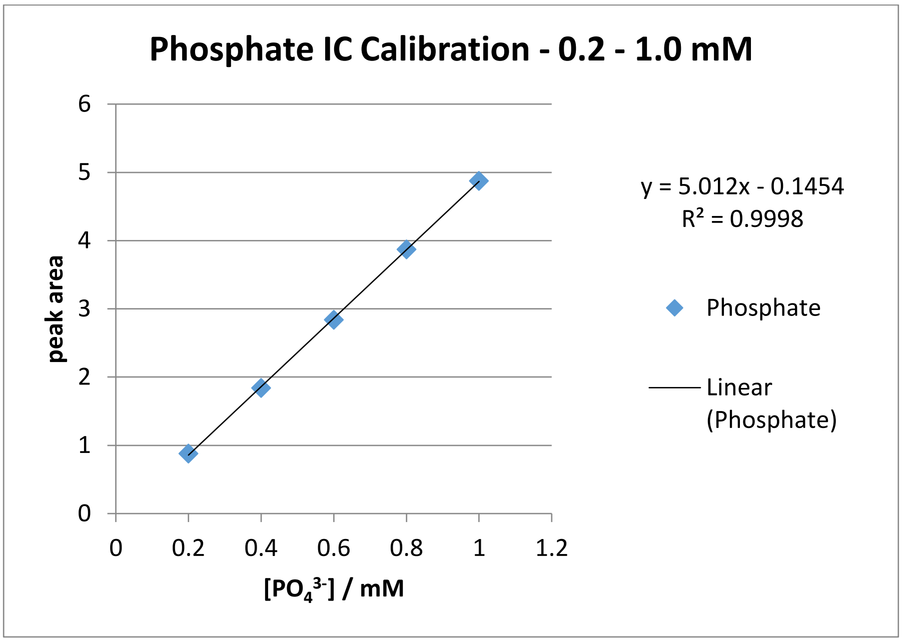

Figure IC-9. IC calibration curve of 0.2 – 1.0 mM solutions of $Na_2HPO_4 \cdot 2H_2O$ for experiment **3 - 8**.

Example chromatograms are shown below:

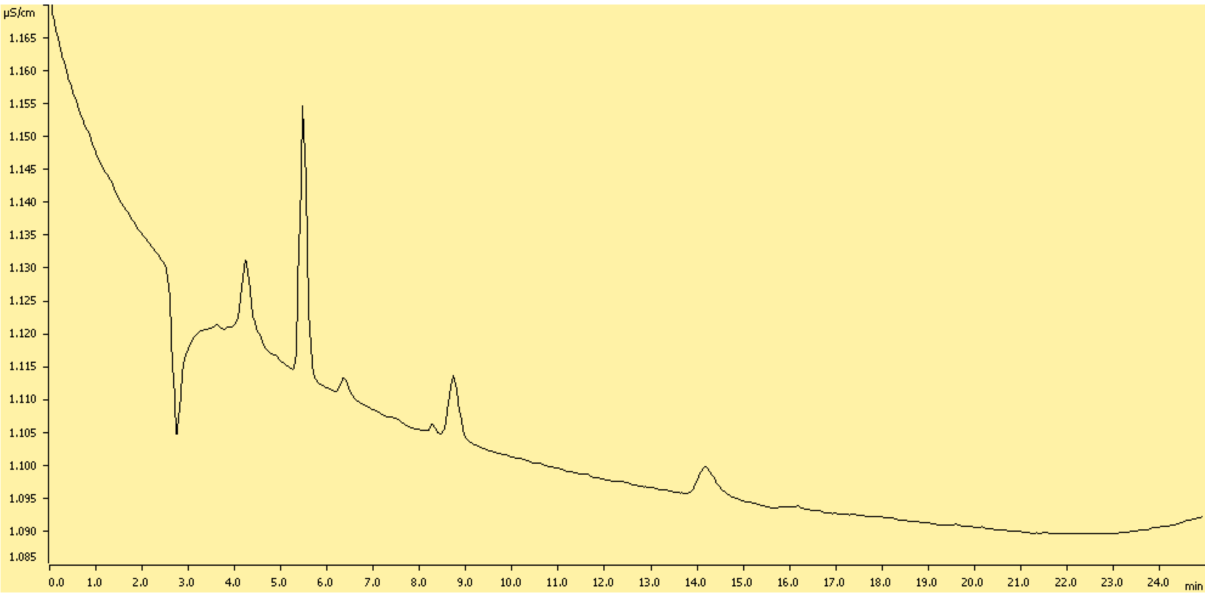

Figure IC-10. Blank

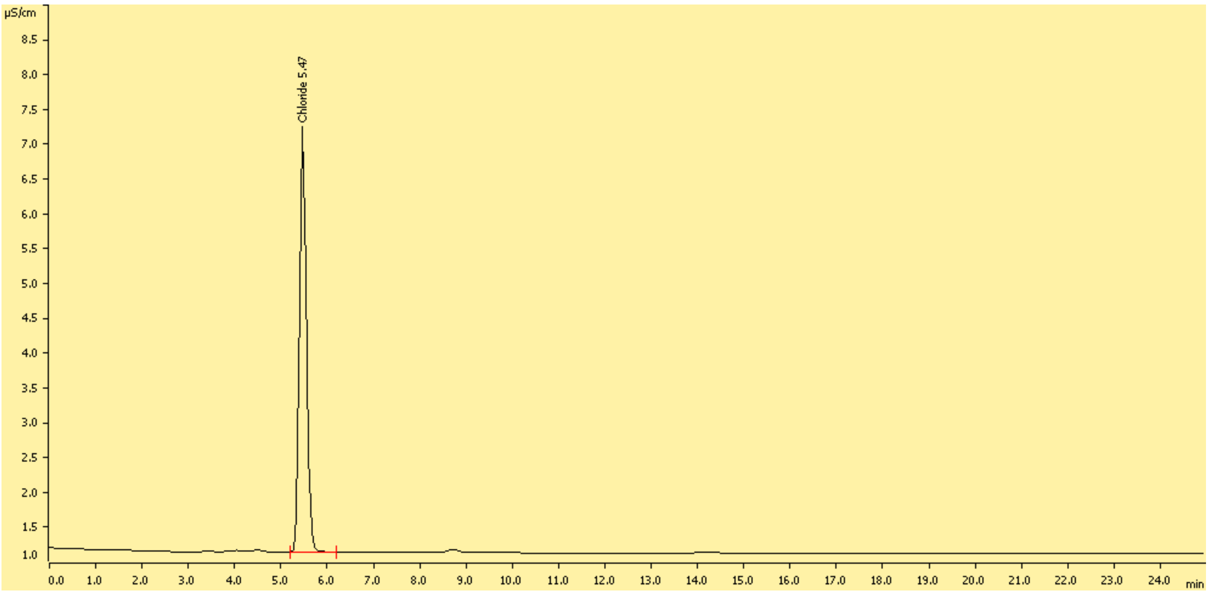

Figure IC-11. An IC plot showing the individual peak for Cl^- ions at 0.6 mM concentration at 5.47 minutes.

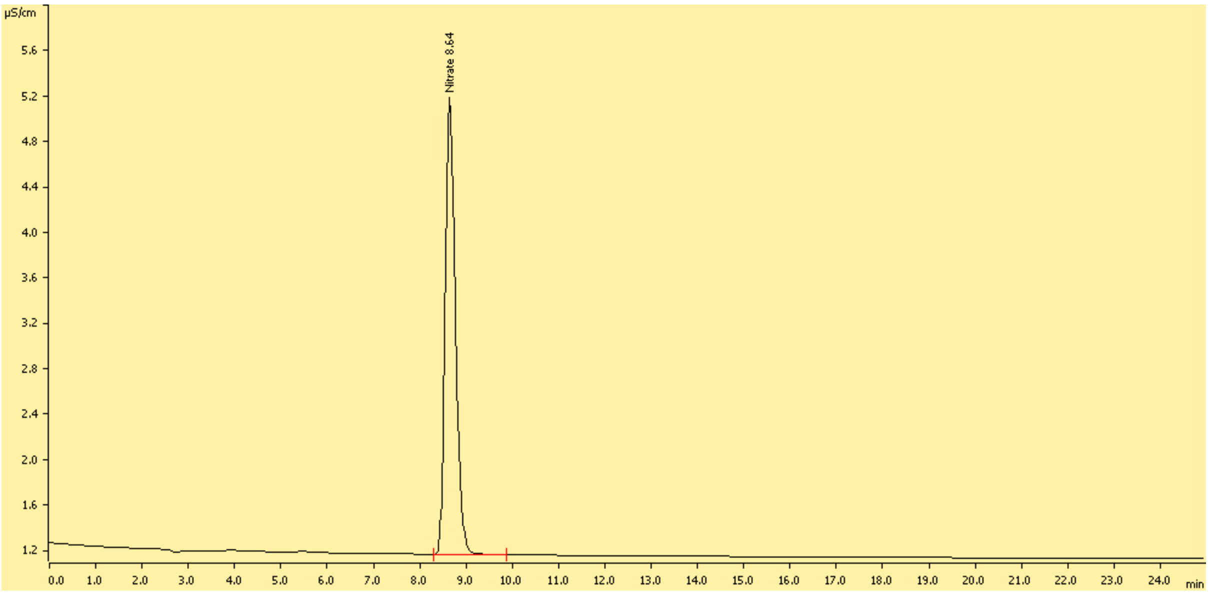

Figure IC-12. An IC plot showing the individual peak for NO_3^- ions at 0.6 mM concentration at 8.64 minutes.

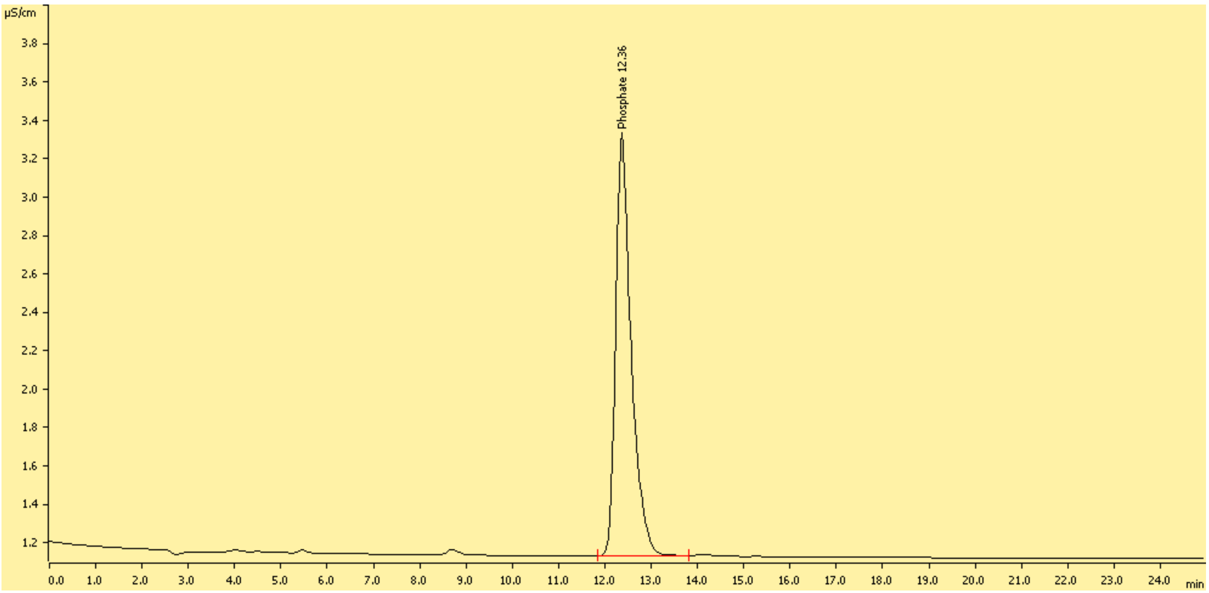

Figure IC-13. An IC plot showing the individual peak for PO_4^{3-} ions at 0.6 mM concentration at 12.36 minutes.

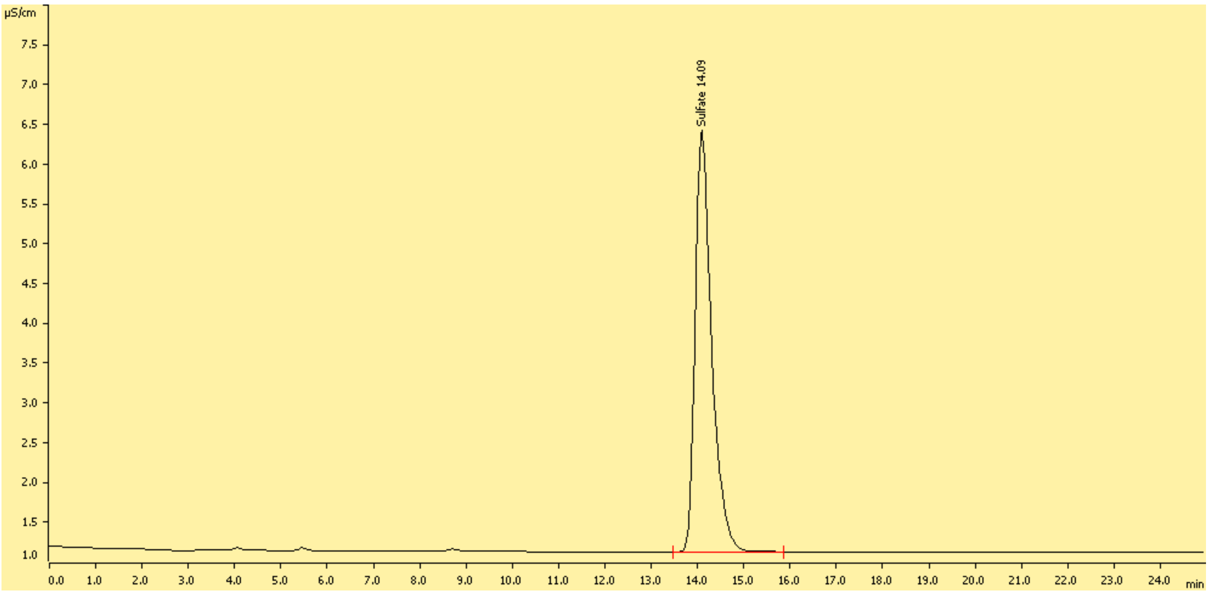

Figure IC-14. An IC plot showing the individual peak for SO_4^{2-} ions at 0.6 mM concentration at 14.09 minutes.

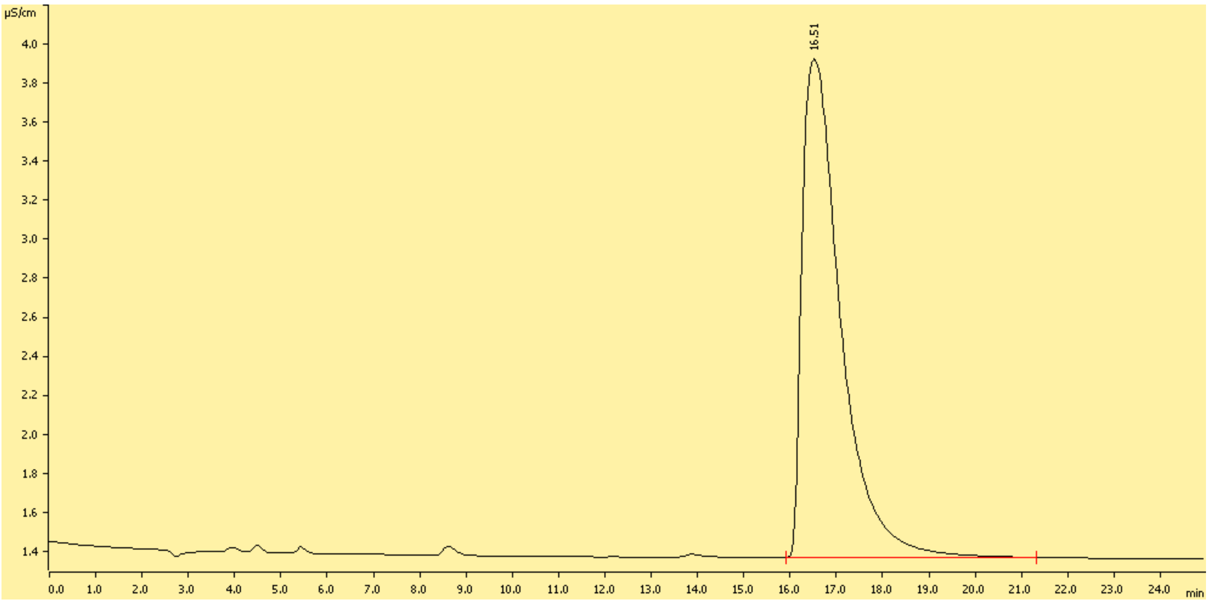

Figure IC-15. An IC plot showing the individual peak for triflate ions at 0.6 mM concentration at 16.51 minutes.

Results and discussion:

Experiment **1**.

<u>Experiment **1** (mono sodium salts):</u>

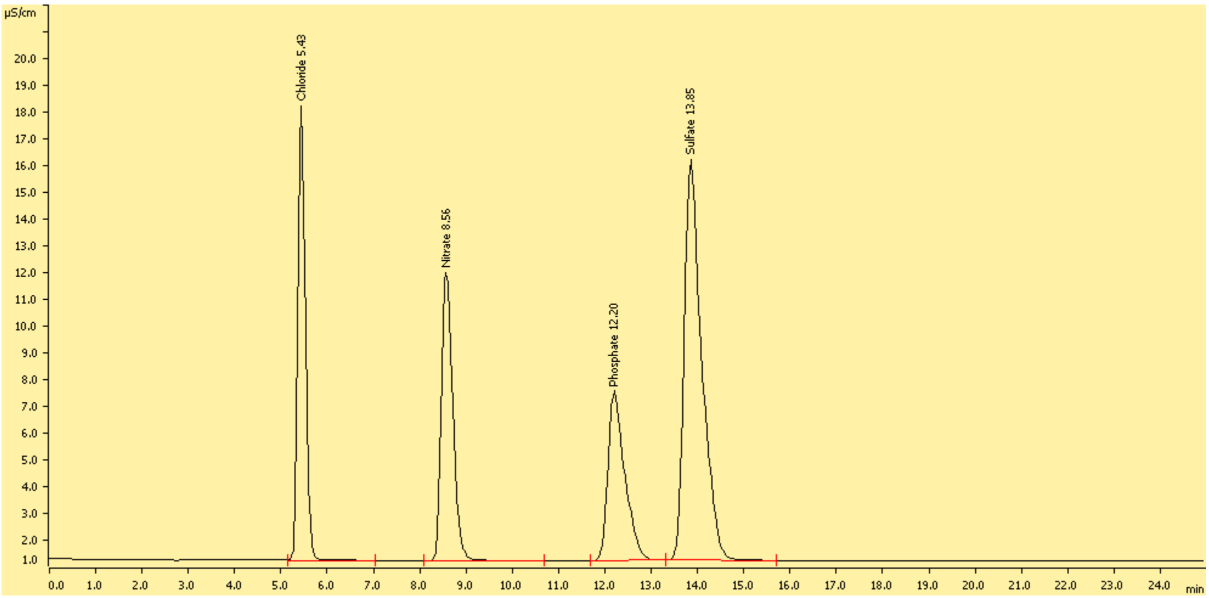

Figure IC-16. The IC chromatogram showing the four individual peaks of the mono-sodium salts at 0.6 mM concentration with the peaks at 5.43, 8.56, 12.20 and 13.85 minutes corresponding to Cl^-, NO_3^- PO_4^{3-} and SO_4^{2-} ions respectively.

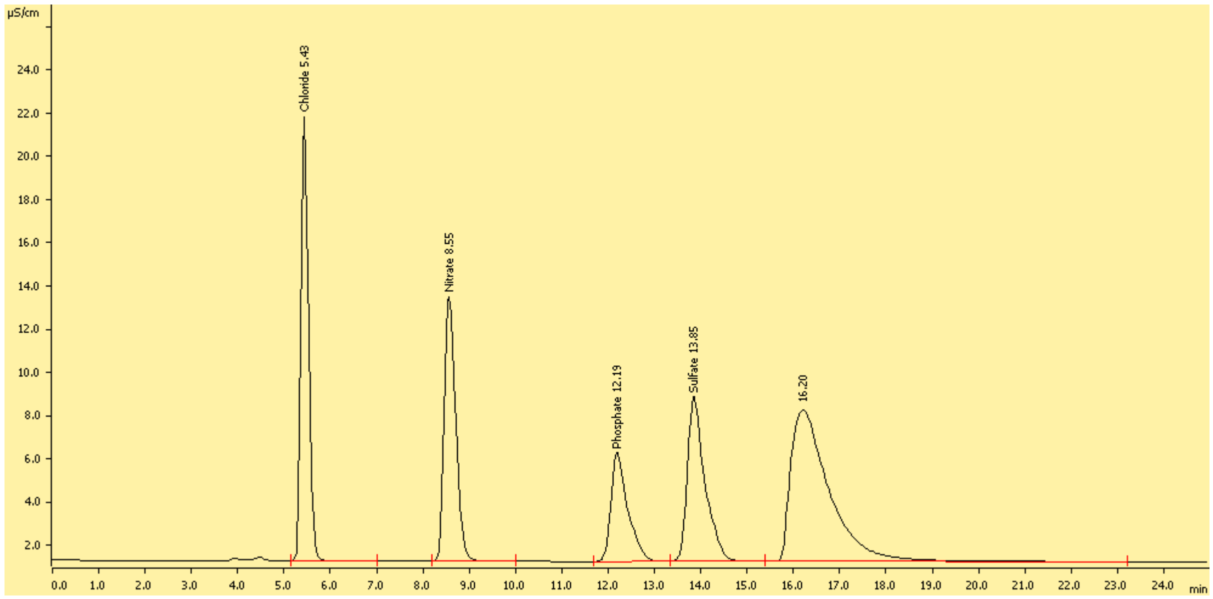

Figure IC-17. The IC chromatogram of experiment **2** after treatment with the [(**L**[1a])$_2$Cu$_3$](trif)$_6$ complex showing the amounts of Cl^-, NO_3^- PO_4^{3-} and SO_4^{2-} ions present. The peak at 16.20 minutes corresponds to triflate anions.

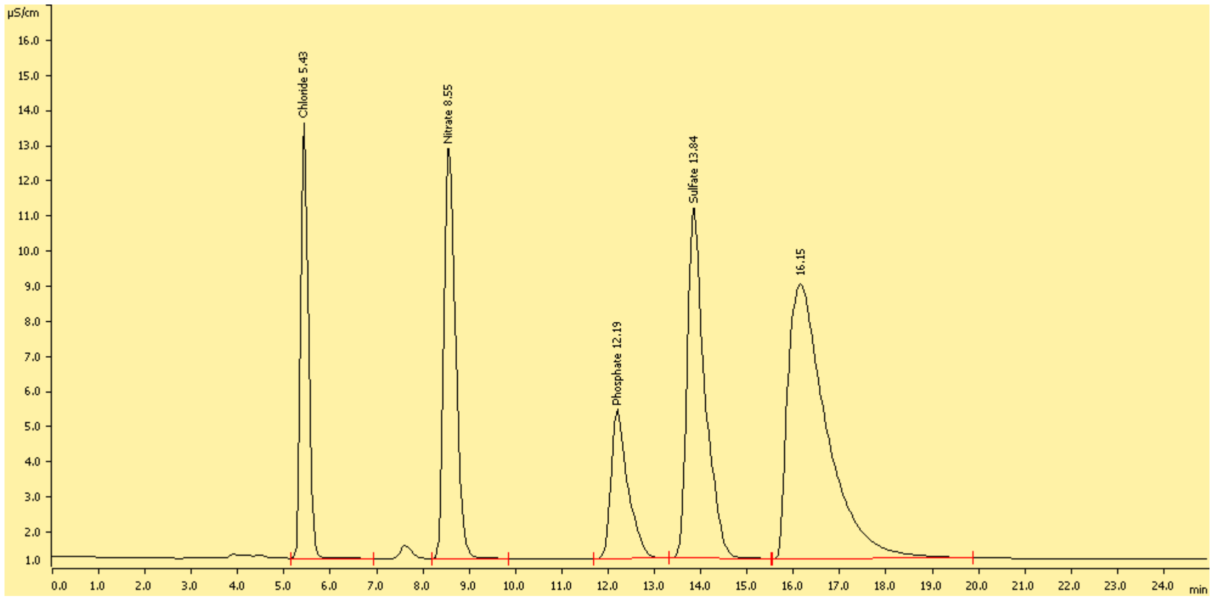

Figure IC-18. The IC chromatogram of experiment **2** after treatment with the [(**L**[2a])$_2$Cu$_3$](trif)$_6$ complex showing the amounts of Cl^-, NO_3^- PO_4^{3-} and SO_4^{2-} ions present. The peak at 16.15 minutes corresponds to the triflate anions.

Salt	Amount of salt present remaining after treatment with [(**L**[1a])$_2$Cu$_3$](trif)$_6$ (mM)	Amount of salt present remaining after treatment with [(**L**[2a])$_2$Cu$_3$](trif)$_6$ (mM)
Cl^-	0.73 (110%)	0.45 (68%)
NO_3^-	0.69 (105%)	0.65 (99%)
PO_4^{3-}	0.48 (73%)	0.41(62%)
SO_4^{2-}	0.31 (47%)	0.40 (61%)

Table 2. A table to show the decrease in the amount of Cl^-, NO_3^- PO_4^{3-} and SO_4^{2-} ions (theoretical maximum concentration of each anion 0.66mM) present in the aqueous solution after treatment with the [(**L**[1a])$_2$Cu$_3$](trif)$_6$ and the [(**L**[2a])$_2$Cu$_3$](trif)$_6$ complexes.

Experiment **2** (disodium salts):

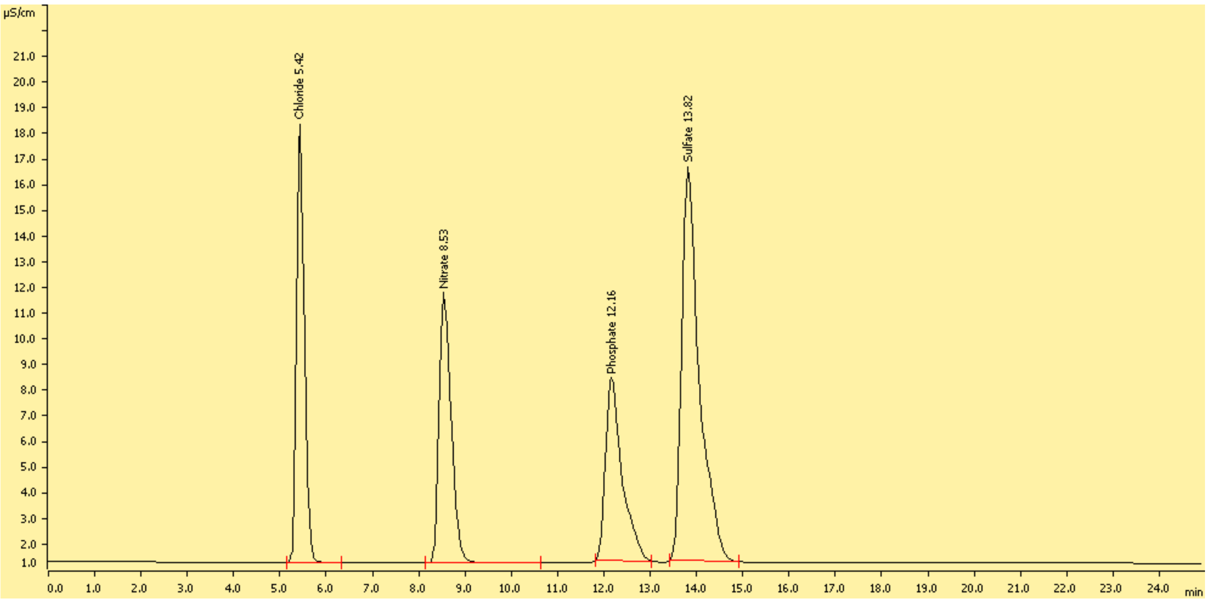

Figure IC-19. The IC chromatogram plot showing the four individual peaks of the di-sodium salts at 0.6 mM concentration with the peaks at 5.42, 8.53, 12.16 and 13.82 minutes corresponding to Cl^-, NO_3^- PO_4^{3-} and SO_4^{2-} ions respectively.

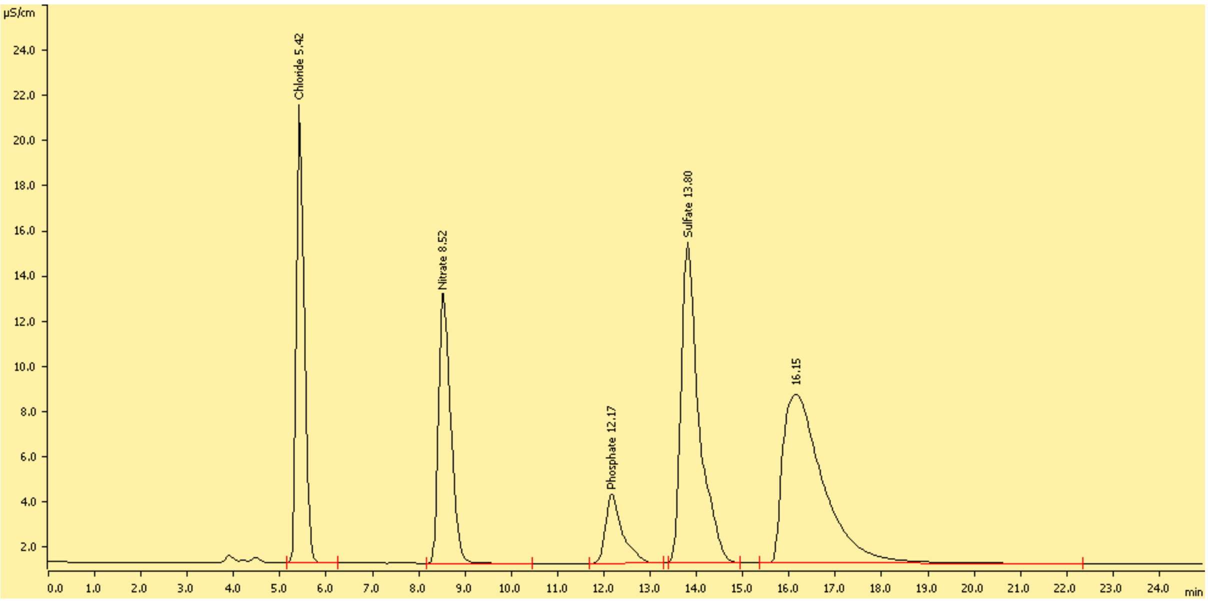

Figure IC-20. The IC chromatogram of experiment **2** after treatment with the $[(\mathbf{L^{1a}})_2Cu_3](trif)_6$ complex showing the decrease in amounts of Cl^-, NO_3^- PO_4^{3-} and SO_4^{2-} ions present. The peak at 16.15 minutes corresponds to triflate anions.

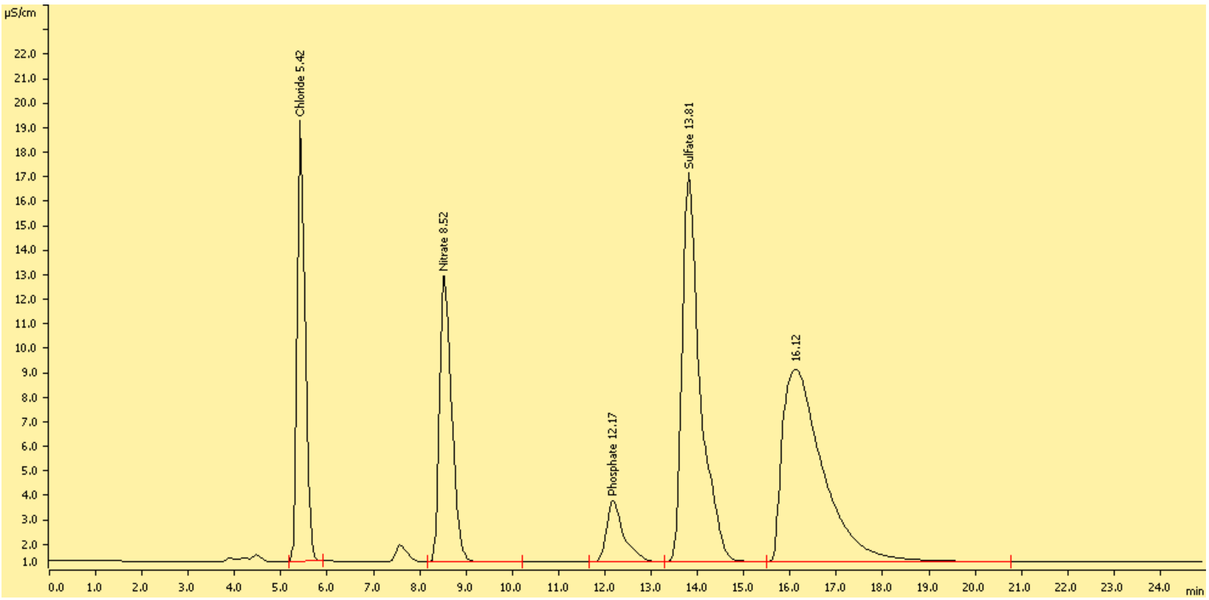

Figure IC-21. The IC chromatogram of experiment **2** after treatment with the $[(\mathbf{L^{2a}})_2Cu_3](trif)_6$ complex showing the decrease in amounts of Cl^-, NO_3^- PO_4^{3-} and SO_4^{2-} ions present. The peak at 16.12 minutes corresponds to triflate anions.

Salt	Amount of salt present remaining after treatment with $[(\mathbf{L^{1a}})_2Cu_3](trif)_6$ (mM)	Amount of salt present remaining after treatment with $[(\mathbf{L^{2a}})_2Cu_3](trif)_6$ (mM)
Cl^-	0.71 (107%)	0.64 (97%)
NO_3^-	0.67 (101%)	0.66 (100%)
PO_4^{3-}	0.27 (41%)	0.24 (36%)
SO_4^{2-}	0.56 (85%)	0.63 (95%)

Table 3. A table to show the decrease in the amount of Cl^-, NO_3^- PO_4^{3-} and SO_4^{2-} ions present (theoretical maximum concentration of each anion 0.66mM) in the aqueous solution after treatment with the $[(\mathbf{L^{1a}})_2Cu_3](trif)_6$ and the $[(\mathbf{L^{2a}})_2Cu_3](trif)_6$ complexes.

Experiments **3** - **5**:

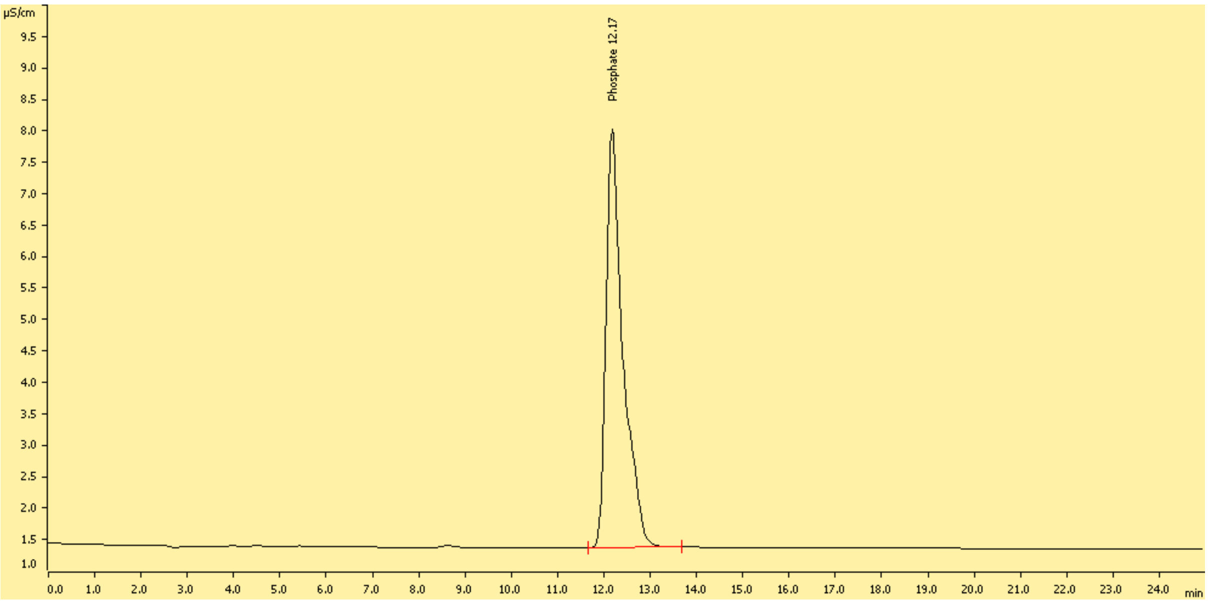

Figure IC-22. An IC plot showing the individual peak for PO_4^{3-} ions at 0.66 mM concentration at 12.17 minutes.

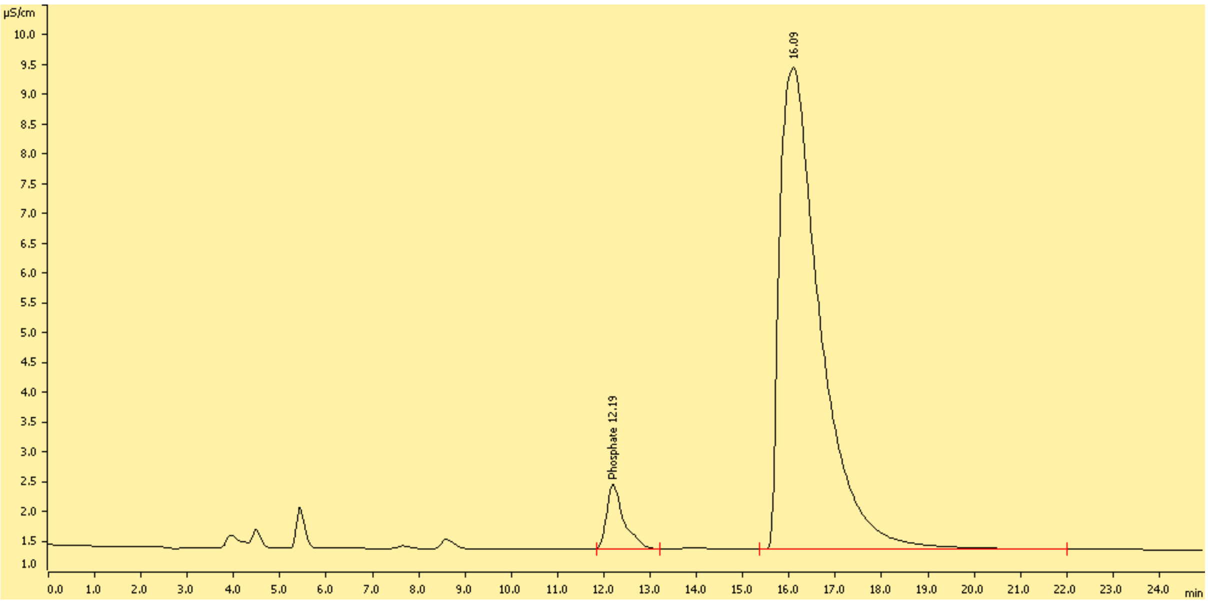

Figure IC-23. The IC chromatogram for experiment **3** showing the total amount of PO_4^{3-} ions left in the aqueous solution after treatment with the [(**L**^{1a})$_2$Cu$_3$](trif)$_6$ complex. The peak at 16.09 minutes corresponds to triflate anions.

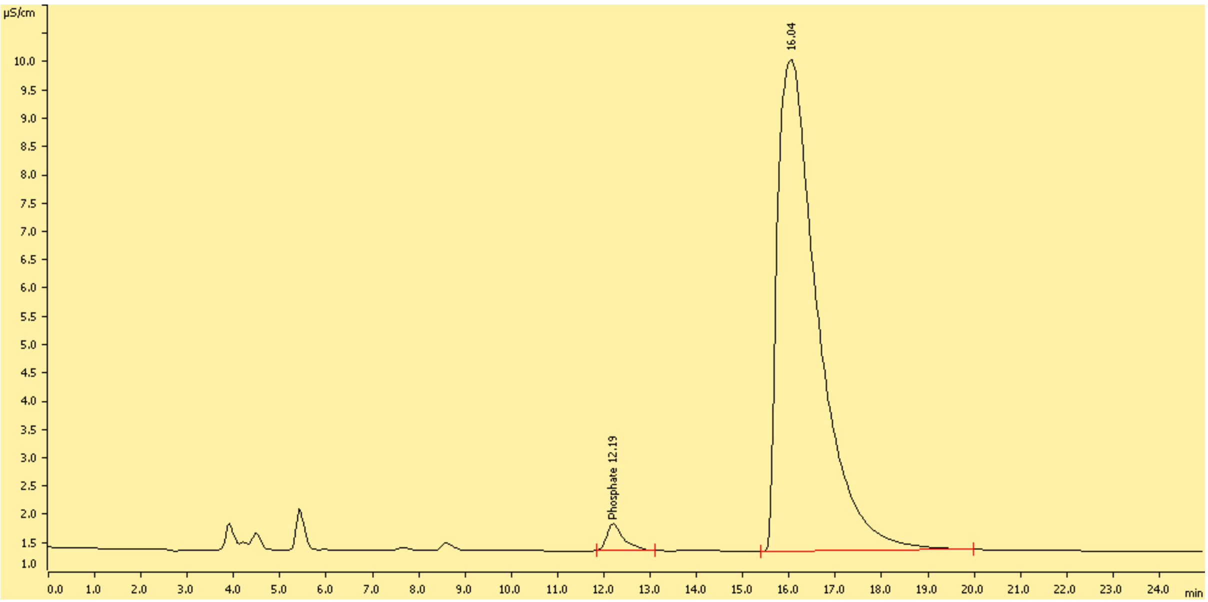

Figure IC-24. The IC chromatogram for experiment **4** showing the total amount of PO_4^{3-} ions left in the aqueous solution after treatment with the [(**L**^{1a})$_2$Cu$_3$](trif)$_6$ complex where the complex was formed from the corresponding masses of ligand and Cu(trif)$_2$ in Table 1. The peak at 16.04 minutes corresponds to triflate anions.

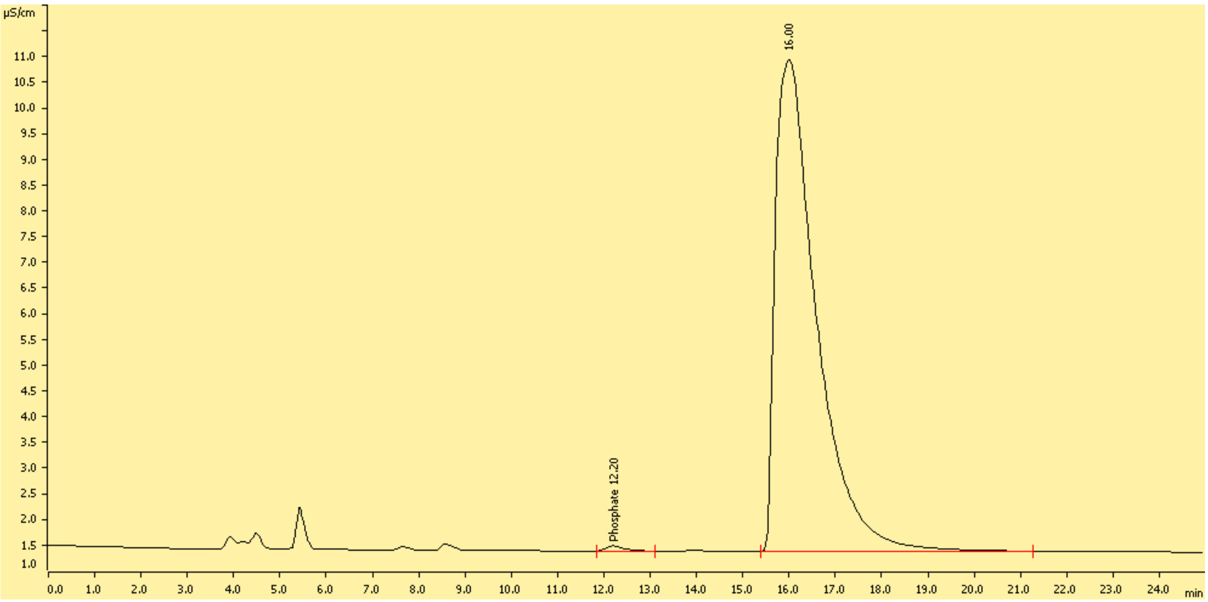

Figure IC-25. The IC chromatogram for experiment **5** showing the total amount of PO_4^{3-} ions left in the aqueous solution after treatment with the [(**L**^{1a})$_2$Cu$_3$](trif)$_6$ complex where the complex was formed from the corresponding masses of ligand and Cu(trif)$_2$ in Table 1. The peak at 16.00 minutes corresponds to triflate anions.

Experiment no.	Amount of PO_4^{3-} ions present (mM) after treatment with the corresponding stoichiometric amount of [(**L**^{1a})$_2$Cu$_3$](trif)$_6$ detailed in table 1
**3**	0.122 (18%)
**4**	0.07 (11%)
**5**	0.038 (6%)

Table 4. A table showing the decrease in the amount of PO_4^{3-} ions present in the aqueous solution as the ratio of the [(**L**^{1a})$_2$Cu$_3$](trif)$_6$ complex present increases (initial concentration of PO_4^{3-} = 0.66mM).

Experiments **6 - 8**:

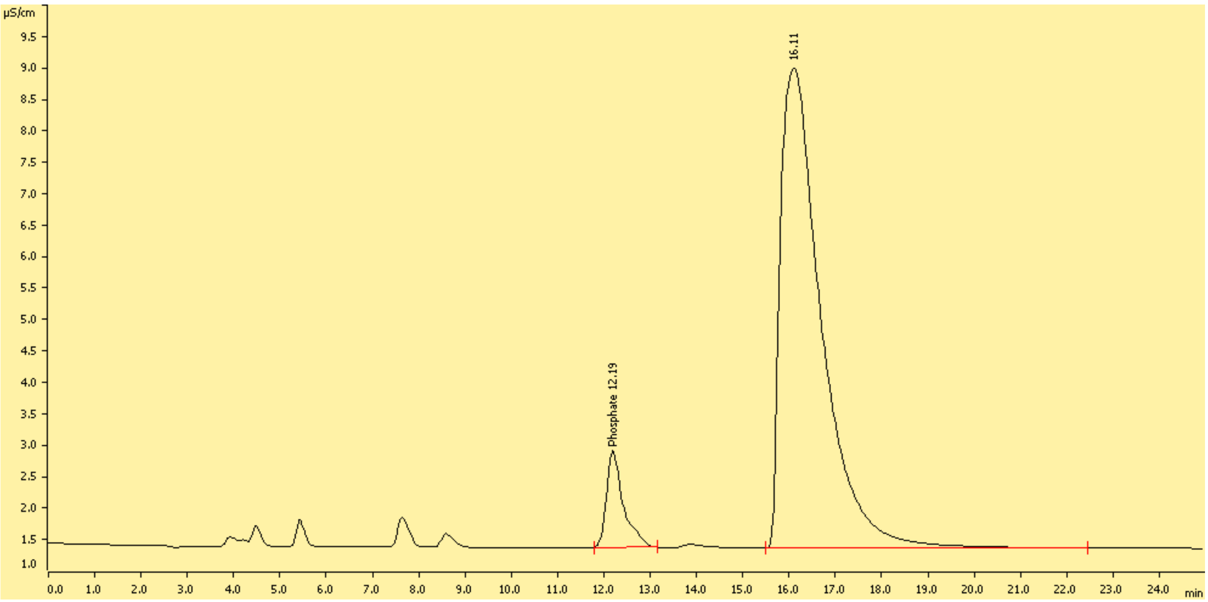

Figure IC-26. The IC chromatogram for experiment **6** showing the total amount of PO_4^{3-} ions left in the aqueous solution after treatment with the $[(\mathbf{L}^{2a})_2Cu_3](trif)_6$ complex where the complex was formed from the corresponding masses of ligand and $Cu(trif)_2$ in Table 1. The peak at 16.11 minutes corresponds to triflate anions.

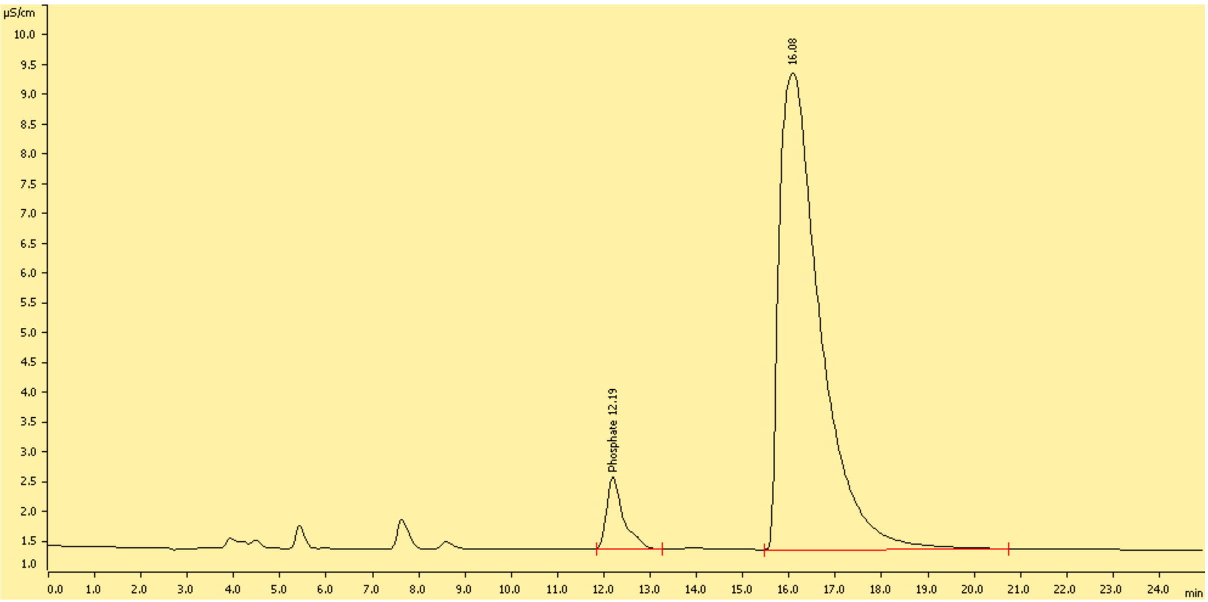

Figure IC-27. The IC chromatogram for experiment **7** showing the total amount of PO_4^{3-} ions left in the aqueous solution after treatment with the $[(\mathbf{L}^{2a})_2Cu_3](trif)_6$ complex where the complex was formed from the corresponding masses of ligand and $Cu(trif)_2$ in Table 1. The peak at 16.08 minutes corresponds to triflate anions.

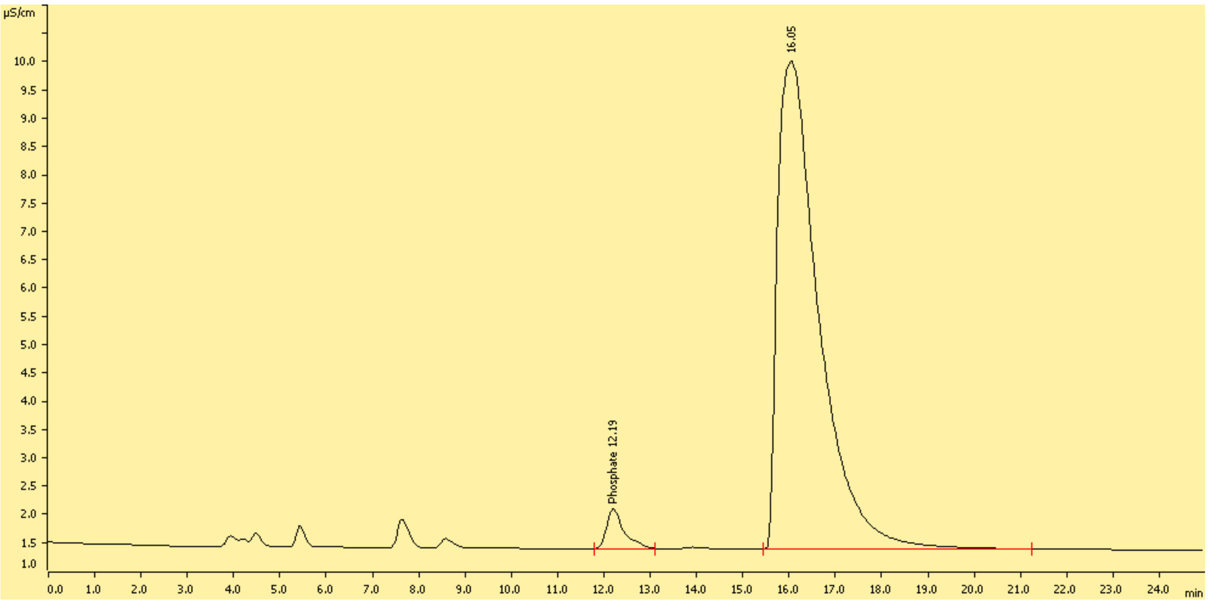

Figure IC-28. The IC chromatogram for experiment **8** showing the total amount of PO_4^{3-} ions left in the aqueous solution after treatment with the $[(\mathbf{L}^{2a})_2Cu_3](trif)_6$ complex where the complex was formed from the corresponding masses of ligand and $Cu(trif)_2$ in Table 1. The peak at 16.05 minutes corresponds to triflate anions.

Experiment no.	Amount of PO_4^{3-} ions present (mM) after treatment with the corresponding stoichiometric amount of $[(\mathbf{L}^{2a})_2Cu_3](trif)_6$ detailed in table 1
**6**	0.159 (24%)
**7**	0.133 (20%)
**8**	0.090 (14%)

Table 5. A table showing the decrease in the amount of PO_4^{3-} ions present in the aqueous solution as the amount of the $[(\mathbf{L}^{2a})_2Cu_3](trif)_6$ complex present increases (initial concentration of PO_4^{3-} = 0.66mM).

BBM Media.

BBM media is comprises the following salts: and $NaNO_3$ (14.7 mM), $CaCl_2 \cdot 2H_2O$ (1.7 mM), $MgSO_4 \cdot 7H_2O$ (1.52 mM), $K_2HPO_4 \cdot 3H_2O$ (2.1l mM), KH_2PO_4 (6.4 mM), NaCl (2.1 mM), vitamin B1 (4.5 x 10^{-3} mM), vitamin B12 (trace). Also contains trace elements $FeCl_3 \cdot 6H_2O$, $MnCl_2 \cdot 4H_2O$, $ZnCl_2$, $CoCl_2 \cdot 6H_2O$ and $Na_2MoO_4 \cdot 2H_2O$ (Each ~ 2 x 10^{-3} mM)

The anions were extracted with a DCM solution of $[(\mathbf{L}^{1a})_2Cu_3](trif)_6$ (1.2 equivalents relative to the total concentration of PO_4^{3-}) in an analogous fashion to that described in Experiment 3.

Anion	BBM (original sample) ppm	BBM (treated) ppm
Cl^-	27.5	26.4 (96%)
NO_3^-	520.7	545.8 (105%)
PO_4^{3-}	168.0	< 5 (< 3%)
SO_4^{2-}	28.4	< 1 (3%)

Table 6. A table showing the change in parts per million of chloride, nitrate, sulfate and phosphate in a BBM solution upon treatment with $[(\mathbf{L}^{1a})_2Cu_3](trif)_6$.

Crystallography.

Single crystal X-ray diffraction data was collected at 150(2) K on a Bruker D8 Venture diffractometer equipped with a graphite monochromated Mo(Kα) radiation source and a cold stream of N_2 gas. Solutions were generated by conventional heavy atom Patterson or direct methods and refined by full-matrix least squares on all F^2 data, using SHELXS-97 and SHELXL software respectively.[C1] Absorption corrections were applied based on multiple and symmetry-equivalent measurements using SADABS.[C2]

Crystal for $[(\mathbf{L}^2)_2Cu_3(SO_4)](ClO_4)_4$. Crystal data for $C_{67.12}H_{66}Cl_4Cu_3N_{23}O_{29.75}S_7$, M = 2227.75, *monoclinic*, a = 30.9946 (8), b = 26.3900 (7), c = 25.3578 (6) Å, β = 120.8910 (10)°, V = 17799.1 (8) Å^3, T = 150 K, space group *C2/c*, Z = 8, 100877 reflections measured, 17510 independent reflections (R_{int} = 0.0616). The final R_1 values were 0.0536 ($I > 2\sigma(I)$). The final $wR(F^2)$ values were 0.1425 ($I > 2\sigma(I)$). The final R_1 values were 0.0679 (all data). The final $wR(F^2)$ = 0.1528 (all data). The goodness of fit on F^2 was 1.018. Peak and hole = 1.632 / -1.042. CCDC 1941880. The structure contained a two disordered perchlorate counter anions and disordered diethyl ether and nitromethane solvent molecules. The disorder was modelled in two positions using the *PART* instruction in the l.s. refinement and for three $MeNO_2$ molecules bond lengths were restrained by the *DFIX* and *SADI* instruction. The anisotropic displacement parameters were restrained using the *DELU*, *SIMU* and in some cases *ISOR* instructions.

Crystal for $[(\mathbf{L}^2)_2Cu_3Br](BF_4)_5$. Crystal data for $C_{60}H_{54}B_{4.538}BrCu_3F_{18.153}N_{18}O_3S_6$, M = 1932.16, *triclinic*, a = 30.483 (7), b = 25.801 (6), c = 23.930 (6) Å, β = 120.638 (9)°, V = 16193 (7) Å^3, T = 150 K, space group *C2/c*, Z = 8, 94378 reflections measured, 24716 independent reflections (R_{int} = 0.0681). The final R_1 values were 0.0720 ($I > 2\sigma(I)$). The final $wR(F^2)$ values were 0.1864 ($I > 2\sigma(I)$). The final R_1 values were 0.1237 (all data). The final $wR(F^2)$ = 0.2193 (all data). The goodness of fit on F^2 was 1.0676. Peak and hole = 3.4034/ -1.4582. CCDC 1941879. The structure contained a number of disordered tetrafluoroborate counter anions some of which were modelled in two positions using the *PART* instruction in the l.s. refinement. Consequently this lead to a "missing" $0.5BF_4$ (if the charges are to be balanced) however, the oxidation state of the Cu^{2+} ions isn't in question. Furthermore, the diffuse electron density was removed using the solvent mask facility in Olex2, resulting in voids in the crystal structure.[C3] The solvent mask removed a total of 45 electrons per asymmetric unit which corresponds to approximately $0.5BF_4$. Some of the poorly behaved anions were constrained by the *SADI*, *DELU*, *SIMU* and *ISOR* instructions in the least squares refinement and the ADP's of the oxygen solvent molecules were treated similarly.

C1. SHELXTL Program System, Vers. 5.1, Bruker Analytical X-ray Instruments Inc., Madison, WI, 1998.

C2. G. M. Sheldrick, SADABS: A Program for Absorption Correction with the Siemens SMART System, University of Göttingen (Germany), 1996.

C3. O. V. Dolomanov, L. J. Bourhis, R. J. Gildea, J. A. K. Howard and H. Puschmann, OLEX2: a complete structure solution, refinement and analysis program. *J. Appl. Cryst.* (2009). **42**, 339-341.

Computational Details

Geometry optimisations were carried out using Gaussian09 (Revision D.01) and the default values for the calculation parameters and convergence criteria.[M1] Cu centres were describes using the Stuttgart effective core potentials and basis set,[M2] whilst the Pople 6-311G basis set was employed for all other atoms, and the anionic moieties.[M3] Optimisations were carried out using the B3LYP density functional,[M4-8] with dispersion interactions computed through Grimme's D3 parameter set with Becke-Johnson damping.[M9] Solvent effects were computed by employing the integral equation formalism polarised continuum model for DCM.[M10] All stationary points were fully characterised through analytical frequency calculations as minima *i.e.* with positive eigenvalues. All energies were recomputed with a larger basis set featuring LANL2DZ for the Cu centers[M11] and DEF2TZVP for all other atoms.[M12] The optimized geometries are presented below. The formation energy of the complex (comprised of the cage and the anionic moiety) was calculated from the individual empty cage and the ionic moiety as reference; negative values represent a thermodynamically favourable formation. The formation energies of the complexes are -1197 kJ mol^{-1} $[(\mathbf{L^{1a}})_2Cu_3(PO_4)]^{3+}$, -1030 kJ mol^{-1} $[(\mathbf{L^{2a}})_2Cu_3(PO_4)]^{3+}$, -1750 kJ mol^{-1} $[(\mathbf{L^{1a}})_2Cu_3(SO_4)]^{4+}$, and -478 kJ mol^{-1} $[(\mathbf{L^{2a}})_2Cu_3(SO_4)]^{4+}$.

M1. M. J. Frisch, G. W. Trucks, H. B. Schlegel, G. E. Scuseria, M. A. Robb, J. R. Cheeseman, G. Scalmani, V. Barone, B. Mennucci, G. A. Petersson, H. Nakatsuji, M. Caricato, X. Li, H. P. Hratchian, A. F. Izmaylov, J. Bloino, G. Zheng, J. L. Sonnenberg, M. Hada, M. Ehara, K. Toyota, R. Fukuda, J. Hasegawa, M. Ishida, T. Nakajima, Y. Honda, O. Kitao, H. Nakai, T. Vreven, J. A. Montgomery, Jr., J. E. Peralta, F. Ogliaro, M. Bearpark, J. J. Heyd, E. Brothers, K. N. Kudin, V. N. Staroverov, R. Kobayashi, J. Normand, K. Raghavachari, A. Rendell, J. C. Burant, S. S. Iyengar, J. Tomasi, M. Cossi, N. Rega, J. M. Millam, M. Klene, J. E. Knox, J. B. Cross, V. Bakken, C. Adamo, J. Jaramillo, R. Gomperts, R. E. Stratmann, O. Yazyev, A. J. Austin, R. Cammi, C. Pomelli, J. W. Ochterski, R. L. Martin, K. Morokuma, V. G. Zakrzewski, G. A. Voth, P. Salvador, J. J. Dannenberg, S. Dapprich, A. D. Daniels, Ö. Farkas, J. B. Foresman, J. V. Ortiz, J. Cioslowski, and D. J. Fox, Gaussian 09 (Revision D.01); Gaussian Inc.: Wallingford, CT, 2009.

M2. X. Cao, M. Dolg and H. Stoll, J. Chem. Phys., 2003, 118, 487.

M3. R. Krishnan, J. S. Binkley, R. Seeger and J. A. Pople, J. Chem. Phys., 1980, 72, 650.

M4. A. D. Becke, J. Chem. Phys, 1993, 98, 5648–5652.

M5. C. T. Lee, W. T. Yang and R. G. Parr, Phys. Rev. B, 1988, 37, 785–789.

M6. P. J. Stephens, F. J. Devlin, C. F. N. Chabalowski and M. J. Frisch, J. Phys. Chem., 1994, 98, 11623–11627.

M7. E. A. B. Kantchev, T. B. Norsten and M. B. Sullivan, Proc. Int. Conf. Comput. Sci., 2011, 4, 1157–1166.

M8. S. H. Vosko, L. Wilk and M. Nusair, Can. J. Phys., 1980, 58, 1200–1211.

M9. S. Grimme, S. Ehrlich and L. Goerigk, J. Comput. Chem., 2011, 32, 1456–1465.

M10, J. Tomasi, B. Mennucci and E. Cances, J. Mol. Struct., 1999, 464, 211–226.

M11. C. E. Check, T. O. Faust, J. M. Bailey, B. J. Wright, T. M. Gilbert and L. S. Sunderlin, J. Phys. Chem. A, 2001, 105, 8111–8116.

M12, F. Weigend and R. Ahlrichs, Phys. Chem. Chem. Phys., 2005, 7, 3297–3305.

$[(\mathbf{L^1})_2Cu_3]^{6+}$	$[(\mathbf{L^2})_2Cu_3]^{6+}$
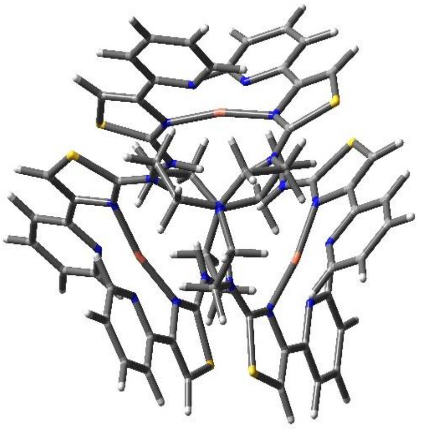	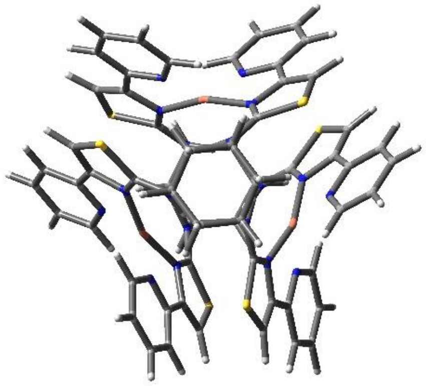
$[(\mathbf{L^1})_2Cu_3(SO_4)]^{4+}$	$[(\mathbf{L^2})_2Cu_3(SO_4)]^{4+}$
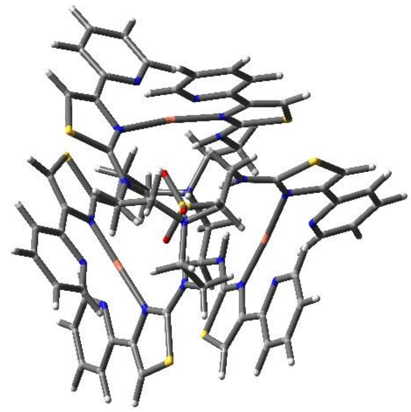	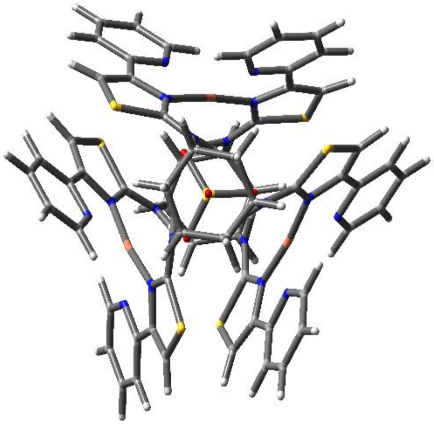
$[(\mathbf{L^1})_2Cu_3(PO_4)]^{3+}$	$[(\mathbf{L^2})_2Cu_3(PO_4)]^{3+}$
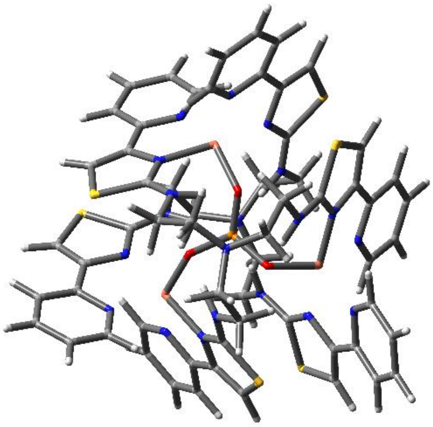	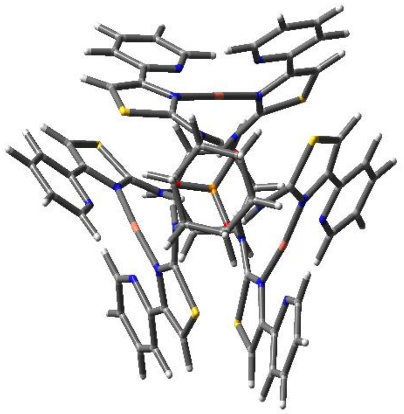

Fig CD1. Computationally Optimised Structures

Cartesian Coordinates for Computationally Optimised Structures

SO_4^{2-}

S	-0.98698300	-0.04338800	0.00000000
O	-2.48256100	-0.03857600	0.00000000
O	-0.48917100	0.66295500	1.22042200
O	-0.48578000	-1.45124300	0.00000000
O	-0.48917100	0.66295500	-1.22042200

PO_4^{3-}

P	-1.50488400	0.58230400	0.00000000
O	-3.07009300	0.57918100	0.00000000
O	-0.97958500	1.32066700	1.27667900
O	-0.98246500	-0.89584300	0.00000000
O	-0.97958500	1.32066700	-1.27667900

Empty Cage $[(L^1)_2Cu_3]^{6+}$

Cu	-0.58737896	1.37528544	2.51480350
Cu	0.61527897	1.68091035	-2.44803765
Cu	0.15577324	-3.00022427	-0.01243752
S	-2.03637284	5.17266118	-1.48890769
S	-3.34521780	-1.82842410	4.05809715
S	-1.40391749	-3.30284616	-4.20906625
N	2.18842390	2.92736848	-2.55705364
N	-0.26338731	3.22591930	-1.67155369
N	-2.36071221	2.48613643	-0.87689995
H	-2.01089345	1.54444247	-0.71074375
N	-4.10176607	0.15026974	-0.95760562
N	-1.87160840	-1.34340022	-2.30413180
H	-1.62247356	-0.80389366	-1.47794305
N	-0.13458069	-2.90977108	-1.92993624
N	1.77385879	-4.06127438	-0.55754102
N	-3.08331865	-0.78602519	1.50232662

H	-2.60222870	-0.26106965	0.77255881
N	-1.45014213	-0.25019493	3.11650630
N	0.63625175	1.03549426	4.06856612
C	3.45545444	2.62390335	-2.92922358
H	3.68908186	1.57710761	-3.05966129
C	4.40175572	3.61635442	-3.14779208
H	5.40749039	3.34782989	-3.43735190
C	4.02303121	4.95548002	-3.00312602
H	4.74197985	5.74619259	-3.16949729
C	2.71308855	5.26797905	-2.64766273
H	2.40034926	6.29589472	-2.53009679
C	1.80716946	4.23295305	-2.42851220
C	0.41056338	4.41728220	-2.04844282
C	-0.34474962	5.53380863	-2.03318286
H	-0.07626116	6.53824294	-2.30896810
C	-1.53268927	3.42579446	-1.31816457
C	-3.82991025	2.62617484	-0.75310138
H	-4.10892761	2.60853417	0.30019204
H	-4.13340094	3.58315801	-1.18051419
C	-4.48344912	1.48795862	-1.53750910
H	-4.12486522	1.53043675	-2.56677706
H	-5.57308094	1.61498008	-1.54553442
C	-5.01958837	-0.15121167	0.19953840
H	-5.12436126	0.75763186	0.79492139
H	-6.01315031	-0.45312056	-0.15508269
C	-4.42756175	-1.24583520	1.09249386
H	-4.34578706	-2.20275430	0.57545801
H	-5.05755998	-1.37503924	1.97543926
C	-2.56957627	-0.85966104	2.72126005
C	-1.96922423	-1.32879558	5.12442493
H	-1.94195706	-1.65608486	6.14865023
C	-1.10394353	-0.53302855	4.46258697

C	0.10925632	0.12000622	4.93659520
C	0.71465262	-0.13027290	6.16701198
H	0.29248838	-0.86407334	6.83819843
C	1.86712858	0.57086478	6.51089463
H	2.35138974	0.38503266	7.45991883
C	2.38934068	1.52071886	5.62557802
H	3.27282774	2.09179603	5.87262458
C	1.74398660	1.73703546	4.41594513
H	2.09236589	2.48212403	3.71753756
C	-4.28226858	-0.90912138	-2.01225706
H	-4.33616307	-1.88065087	-1.52034815
H	-5.21988902	-0.75194188	-2.56155121
C	-3.11440244	-0.92481266	-2.99747750
H	-2.95444246	0.05344460	-3.44908071
H	-3.33131678	-1.64006249	-3.79107259
C	-1.13293411	-2.39620068	-2.64670862
C	-0.00514268	-4.36286345	-3.75785449
H	0.28683647	-5.16588772	-4.41130005
C	0.51915158	-3.99569674	-2.57183235
C	1.65020231	-4.54241996	-1.83041143
C	2.56305658	-5.46439512	-2.33742083
H	2.46083656	-5.82735665	-3.35007053
C	3.60933358	-5.89676656	-1.52556631
H	4.32894056	-6.60917392	-1.90564951
C	3.72260454	-5.40709429	-0.21989352
H	4.51955571	-5.73271448	0.43291778
C	2.78186269	-4.49465298	0.23709973
H	2.81156502	-4.10042753	1.24180595
S	1.93929210	4.72416199	0.80368905
S	2.87379185	-2.07919538	-3.64606186
S	1.23131835	-3.01676927	4.39952804
N	-2.18583805	2.59783372	2.39644310

N	0.30106314	2.79411887	1.54279929
N	2.49644674	2.03583474	1.08673524
H	2.25310891	1.08147721	1.34684710
N	4.39983725	-0.10810900	1.14838864
N	2.38191211	-1.61861517	2.32292030
H	2.43391879	-1.22048696	1.38373853
N	0.35627339	-2.77766156	1.91446105
N	-1.67261435	-3.70070084	0.50015939
N	3.01579550	-0.63483297	-1.31407689
H	2.71209650	0.07479907	-0.64929559
N	1.31755770	-0.08028624	-2.88617922
N	-0.66744462	1.30429569	-3.95360265
C	-3.42349199	2.36272929	2.89470220
H	-3.61469350	1.37760712	3.28996212
C	-4.39115220	3.35868250	2.92972737
H	-5.36942167	3.14292884	3.33454407
C	-4.06953135	4.63176317	2.44821435
H	-4.80525356	5.42433887	2.46474942
C	-2.78718368	4.88133340	1.96203635
H	-2.51272951	5.86303723	1.60459769
C	-1.85303101	3.84655339	1.95458106
C	-0.46683208	3.98886233	1.52895146
C	0.21101323	5.10240745	1.17795488
H	-0.13216637	6.12017301	1.13238862
C	1.56530588	2.98497016	1.17207353
C	3.94669192	2.33005082	0.93428418
H	4.20638045	2.37459387	-0.12567829
H	4.15808178	3.30273124	1.38737534
C	4.73340969	1.25319481	1.68357078
H	4.45483111	1.28546009	2.73955819
H	5.80776021	1.45904077	1.60680511
C	5.14254969	-0.36596252	-0.13137408

H	5.30409359	0.58217795	-0.64535080
H	6.12352469	-0.81307798	0.06465079
C	4.32527669	-1.28763051	-1.03338936
H	4.16249795	-2.26472725	-0.57397938
H	4.85826526	-1.43143547	-1.97722511
C	2.36504264	-0.78831238	-2.46288226
C	1.50830293	-1.55164397	-4.70079596
H	1.35269922	-2.02941180	-5.65107622
C	0.82526443	-0.53013034	-4.14360260
C	-0.31848801	0.19899950	-4.67314389
C	-1.02235317	-0.15786706	-5.82324383
H	-0.74499450	-1.04231661	-6.37830597
C	-2.07967531	0.64658987	-6.24528292
H	-2.63624578	0.38565008	-7.13518874
C	-2.40845803	1.79624556	-5.51942503
H	-3.21052467	2.44759816	-5.83512325
C	-1.67866711	2.10082578	-4.37716635
H	-1.87746028	2.99103316	-3.79986966
C	4.74707503	-1.16477107	2.16122964
H	4.90649688	-2.10511767	1.62961098
H	5.66698168	-0.91173038	2.70020957
C	3.58735472	-1.34862861	3.14295538
H	3.42949372	-0.45900721	3.75808144
H	3.78738147	-2.20268251	3.79410005
C	1.36577577	-2.38472381	2.69363515
C	-0.33066550	-3.78538536	3.91569030
H	-0.88566117	-4.36024864	4.63521283
C	-0.61284695	-3.54623370	2.61767047
C	-1.74768514	-4.00196078	1.82858425
C	-2.83217676	-4.71456242	2.34152123
H	-2.87606712	-4.95084014	3.39434582
C	-3.84690719	-5.12117687	1.47938182

H	-4.69360585	-5.67434598	1.86221742
C	-3.75493598	-4.82120165	0.11560607
H	-4.51586801	-5.13978484	-0.58233569
C	-2.64766371	-4.12104182	-0.34290782
H	-2.51083497	-3.91068398	-1.39059392

Empty Cage $[(L^2)_2Cu_3]^{6+}$

Cu	1.06256843	2.70120417	-0.24410001
Cu	-2.93131278	-0.63125713	-0.03084720
Cu	1.90973503	-2.20275932	0.28579890
N	2.64712759	2.89556982	-1.44468143
N	2.38540650	2.04898025	1.02796269
N	1.17734356	1.35634731	2.94318075
H	0.29792328	1.38146164	2.42190544
N	0.43127415	-1.32762633	3.27947177
H	1.04842148	-0.68977016	2.77464403
N	0.49096501	-2.83633943	1.47214964
N	1.30616863	-3.72688385	-0.86493214
N	-1.61262410	0.67710050	2.91896117
H	-1.29350139	-0.21737734	2.54976313
N	-2.87657960	1.06369329	0.95304132
N	-3.82954048	0.48508787	-1.43048889
N	0.01008671	3.98627410	0.88095359
N	-0.48715804	2.81467348	-1.41612095
N	-0.10583435	1.27851472	-3.16933708
H	0.63109754	0.82055541	-2.62778408
N	-1.30142688	-1.33463133	-2.97813922
H	-1.30969984	-0.39400192	-2.58220504
N	-2.37037584	-2.19053256	-1.05682095
N	-3.47640765	-2.00821038	1.30945002
N	1.54534741	-0.98115642	-2.89259701
H	0.80722595	-1.47729422	-2.38825370

N	2.89699566	-1.08073448	-0.96414350
N	3.40180923	-1.69512715	1.52975930
C	2.62499571	3.37058781	-2.71505989
H	1.65731646	3.46751515	-3.18364218
C	3.79130548	3.73877131	-3.37033611
H	3.74397129	4.10871400	-4.38429383
C	5.00965080	3.63785835	-2.69050873
H	5.93143829	3.92630516	-3.17713754
C	5.02969176	3.17871398	-1.37555110
H	5.95826760	3.11618990	-0.82592348
C	3.83176274	2.80778407	-0.76768039
C	3.70767573	2.35348396	0.61282827
C	4.64786497	2.23624252	1.57436650
H	5.70114881	2.44616431	1.52492433
C	2.30730794	1.68774733	2.30522893
C	1.02487011	1.40835007	4.44447693
H	1.74358093	2.14556612	4.81534287
C	1.31011967	0.04444200	5.10228918
H	2.31045322	-0.31133115	4.83845076
H	1.28774246	0.18858217	6.18613181
C	0.25531774	-1.01289827	4.73582947
H	0.44785005	-1.93128669	5.29927291
C	0.09474713	-2.47694092	2.69136657
C	-0.82957939	-4.69877168	1.96884893
H	-1.31167367	-5.65831280	1.91434101
C	-0.03052134	-4.09130650	1.06648632
C	0.43617217	-4.56789744	-0.22777728
C	0.10959580	-5.80625181	-0.77717704
H	-0.56994683	-6.46597811	-0.25658678
C	0.68951917	-6.18916371	-1.98403981
H	0.44756014	-7.14830422	-2.42113578
C	1.60370535	-5.33756060	-2.61368077

H	2.09246079	-5.61892350	-3.53531217
C	1.89846434	-4.11749009	-2.02318111
H	2.62797640	-3.44625689	-2.44992705
C	-1.16855523	-0.50988388	5.04595357
H	-1.92073174	-1.24220785	4.74278689
H	-1.24944139	-0.39740375	6.13142507
C	-1.47595464	0.85526715	4.41056541
H	-2.44570040	1.20253856	4.78127559
C	-0.39691229	1.89505564	4.76460928
H	-0.45425073	2.07641941	5.84216170
H	-0.59132036	2.84531534	4.26051632
C	-2.44870167	1.40541049	2.16348320
C	-3.94666986	3.13376581	1.12120390
H	-4.57599672	3.98218921	0.92024618
C	-3.71534248	2.04319428	0.36053145
C	-4.23372192	1.70236228	-0.95804260
C	-5.10538674	2.50085857	-1.69563738
H	-5.42591479	3.45728127	-1.30866740
C	-5.56764204	2.04487659	-2.92776385
H	-6.24690926	2.65255728	-3.50968277
C	-5.16201882	0.79169794	-3.39721566
H	-5.51949941	0.40226499	-4.33944888
C	-4.29927313	0.03233990	-2.62035338
H	-3.99151729	-0.95668160	-2.92449149
C	0.41229890	4.51871514	2.06153712
H	1.29496480	4.09407442	2.51404078
C	-0.25561893	5.58927076	2.63830563
H	0.08759013	5.99325451	3.57961941
C	-1.35462087	6.13886635	1.97033553
H	-1.88674035	6.97901963	2.39545281
C	-1.75316660	5.60886504	0.74564177
H	-2.58736341	6.03659137	0.20825328

C	-1.05614609	4.52356065	0.21623908
C	-1.34063096	3.89611433	-1.06851767
C	-2.25426794	4.22782265	-2.00466717
H	-2.97108514	5.02896104	-2.00018871
C	-0.74830846	2.31469136	-2.62197212
C	-0.10561467	0.97025895	-4.64405122
H	-0.23044090	1.91984021	-5.17261085
C	-1.24949683	0.01589699	-5.03928719
H	-2.22024914	0.42124650	-4.73999079
H	-1.25288416	-0.06089799	-6.13099958
C	-1.07093239	-1.39775290	-4.46264989
H	-1.84343651	-2.05146950	-4.87907037
C	-1.87684620	-2.32225404	-2.28385823
C	-2.82908606	-4.45768072	-1.36311994
H	-3.18888122	-5.46019367	-1.21921374
C	-2.90466220	-3.39470988	-0.53436096
C	-3.49723444	-3.27477334	0.79277065
C	-4.08241658	-4.32449000	1.49824423
H	-4.10555365	-5.31795841	1.07235311
C	-4.64518236	-4.07289758	2.74740912
H	-5.10139068	-4.87839019	3.30653662
C	-4.62853488	-2.77346022	3.26494519
H	-5.07024245	-2.54648362	4.22453056
C	-4.04395648	-1.76101413	2.51706380
H	-4.04202984	-0.73575555	2.85725223
C	0.31766423	-1.97917413	-4.78806516
H	0.45140110	-2.95165556	-4.30436554
H	0.37126829	-2.14065443	-5.86835018
C	1.46238562	-1.03299912	-4.39792233
H	2.40785277	-1.44991687	-4.75845954
C	1.26363455	0.36896183	-5.00151398
H	1.32116234	0.27157121	-6.08959015

H	2.06511310	1.04722685	-4.69587128
C	2.69747168	-0.77140008	-2.23913077
C	4.98802222	-0.15547012	-1.41568156
H	6.01075493	0.16361779	-1.32791054
C	4.18265813	-0.72415152	-0.49331786
C	4.44493132	-1.07729165	0.89436785
C	5.65835210	-0.86822001	1.54555249
H	6.47241187	-0.37827366	1.03070526
C	5.81036049	-1.31180047	2.85666039
H	6.74664353	-1.15739290	3.37546072
C	4.75188202	-1.97044747	3.48983144
H	4.84823955	-2.34514361	4.49846565
C	3.56284059	-2.15353841	2.79749369
H	2.73317683	-2.68922371	3.23404349
S	3.91950538	1.68980532	3.13865880
S	-1.01024803	-3.69310643	3.46415298
S	-3.06926471	3.02784039	2.69885155
S	-2.12810763	3.15316363	-3.45580355
S	-2.03114447	-4.01467382	-2.92814721
S	4.14146748	0.01261698	-3.00744808

$[(L^1)_2Cu_3PO_4]^{3+}$

Cu	-0.03899000	-2.78579000	0.95219000
Cu	2.54501000	1.46121000	-0.50681000
Cu	-2.67099000	1.45521000	-0.34281000
S	4.14444330	-1.57846495	2.36742749
S	-4.02347630	-1.71093516	2.67591667
S	-0.12618558	5.08359495	0.32861440
S	-3.76254808	-4.19928903	-1.33510510
S	-1.46566693	3.92560645	-4.03934060
S	4.75599612	-2.15205744	-2.28747894
N	0.92284760	-4.15759128	-0.27716851

N	1.82922946	-2.47655871	1.49140281
N	1.56703071	-0.89958405	3.20817341
H	0.60041494	-0.74704531	2.86037464
N	0.07422323	1.43278205	4.33218876
N	-2.39027430	0.54649816	2.87790203
H	-1.71192635	1.12338524	2.34527033
N	-3.45071694	0.08364621	0.83639896
N	-4.03942708	0.60134862	-1.64328001
N	0.91165570	3.06029408	1.94901240
H	1.16762654	2.05402115	1.93727242
N	1.52824445	3.13863424	-0.31433907
N	2.78331569	2.25218997	-2.41504574
N	-0.81223771	-3.90710319	2.60299740
N	-1.82349048	-3.28225086	0.21407118
N	-1.94665649	-2.13692271	-1.85056861
H	-1.20060999	-1.44369543	-1.56502125
N	-0.22205156	-1.30216601	-4.13526507
N	-0.55915042	1.45680909	-3.09047759
H	-0.41988879	0.73519082	-2.33096841
N	-2.02765973	2.73035723	-1.74483019
N	-3.57193656	3.26131155	0.37113333
N	2.03807327	-1.50672821	-2.19840596
H	1.20085870	-1.00263326	-1.79703052
N	3.57271039	-0.17781325	-0.98336269
N	4.35582013	1.86356345	0.54286606
O	-0.52868445	-0.98425729	1.42841512
O	1.44589574	0.61458466	0.82903602
O	-1.02850071	1.46694481	0.66079365
O	-0.12537605	-0.32465384	-1.16348824
P	-0.06299000	0.18021000	0.38219000
C	0.32685890	-5.02681248	-1.12656283
H	-0.75165590	-5.02658529	-1.13854678

C	1.06279967	-5.87826654	-1.94252513
H	0.55313546	-6.55868376	-2.60993057
C	2.45860033	-5.83287842	-1.87923383
H	3.05683955	-6.48278612	-2.50384688
C	3.07613017	-4.94723820	-0.99978330
H	4.15219634	-4.89830013	-0.92486652
C	2.28603835	-4.11677611	-0.20296691
C	2.81095163	-3.16073961	0.76315019
C	4.09177897	-2.84927307	1.07103191
H	5.00686049	-3.23916708	0.66602328
C	2.31098076	-1.62903153	2.38932246
C	2.14588789	0.07193399	4.14870437
H	2.59546286	0.91129882	3.60375677
H	2.92564226	-0.42438225	4.73504051
C	1.03229769	0.55652545	5.07612633
H	0.49055697	-0.32204327	5.43597176
H	1.45861520	1.07422473	5.94632807
C	-1.30201329	1.31900010	4.90834443
H	-1.84685666	2.24200526	4.69327213
H	-1.27003516	1.19010068	5.99874190
C	-2.06103247	0.16190576	4.25956319
H	-1.45941316	-0.75577733	4.26636773
H	-2.99116756	-0.02394367	4.80610632
C	-3.17826768	-0.18713404	2.10486341
C	-4.77560508	-1.82479401	1.02752711
H	-5.46164210	-2.61992972	0.80419527
C	-4.34295734	-0.81511561	0.23723863
C	-4.68042548	-0.49597121	-1.14307664
C	-5.58865182	-1.21657079	-1.92062869
H	-6.08113911	-2.08426884	-1.50694157
C	-5.84841006	-0.79906636	-3.22346158
H	-6.54965198	-1.34670783	-3.83892313

C	-5.20134282	0.33437103	-3.72454064
H	-5.38687601	0.68909959	-4.72848403
C	-4.30352053	1.01031810	-2.90629405
H	-3.78066740	1.89057524	-3.24572190
C	0.53685487	2.85596590	4.34176416
H	1.62962053	2.86741560	4.37376894
H	0.16550009	3.39137892	5.22594128
C	0.10026406	3.57290126	3.06452766
H	-0.96607301	3.40935785	2.86433361
H	0.27879676	4.64821781	3.16524040
C	0.87068747	3.60151094	0.73929091
C	0.52208285	4.96193471	-1.36260481
H	0.23100447	5.68477833	-2.10111884
C	1.34168301	3.89139420	-1.48190902
C	2.07639245	3.39880502	-2.63951928
C	2.08946207	4.02012646	-3.88991055
H	1.52216545	4.92559955	-4.04748399
C	2.84152901	3.45909037	-4.91895037
H	2.86133920	3.92754901	-5.89396117
C	3.57274052	2.29169299	-4.67925646
H	4.17256048	1.83586899	-5.45423248
C	3.52161348	1.71697726	-3.41477689
H	4.07327293	0.82047962	-3.17875080
C	-0.28290650	-4.01812978	3.83995748
H	0.64575981	-3.48984604	4.01044199
C	-0.90583299	-4.75991982	4.83831641
H	-0.46381261	-4.82785572	5.82260579
C	-2.10692129	-5.41111312	4.53815875
H	-2.61359445	-5.99787215	5.29317009
C	-2.65606408	-5.29178463	3.26340086
H	-3.59404784	-5.77113733	3.01877583
C	-1.98717365	-4.52125885	2.30981194

C	-2.49110050	-4.27736780	0.95601780
C	-3.51848904	-4.88815104	0.32505508
H	-4.14600455	-5.68966460	0.67007015
C	-2.34761738	-3.05373592	-0.98605245
C	-2.41205932	-2.05358630	-3.24754667
H	-2.78874877	-1.04672055	-3.42755288
H	-3.23085396	-2.75806995	-3.40408960
C	-1.25691624	-2.37147245	-4.20456848
H	-0.80419696	-3.31507353	-3.89010370
H	-1.64275218	-2.50122563	-5.22761480
C	-0.58437586	-0.12721395	-4.97568609
H	-1.67070295	-0.00923012	-4.96001382
H	-0.27039803	-0.26067819	-6.02273232
C	0.04175951	1.15041492	-4.40228663
H	1.11733102	1.02602064	-4.27289386
H	-0.12706111	1.98404991	-5.08649419
C	-1.30847019	2.51782968	-2.84286106
C	-2.52472301	4.74818111	-2.81794697
H	-2.94791557	5.71149874	-3.03887088
C	-2.67438472	3.98282522	-1.71363873
C	-3.41833539	4.29804593	-0.49154702
C	-3.91000711	5.56913166	-0.18642415
H	-3.77254612	6.38818057	-0.87862719
C	-4.55493105	5.76713335	1.03241944
H	-4.93740251	6.74636578	1.28947032
C	-4.69250175	4.69843888	1.92442721
H	-5.17909158	4.82747457	2.88124561
C	-4.18987217	3.45477026	1.55573047
H	-4.26229732	2.59075637	2.20310838
C	1.13227657	-1.82261712	-4.47053723
H	1.76824953	-0.98020277	-4.75328308
H	1.10149218	-2.52713374	-5.31640856

C	1.75661623	-2.50500983	-3.24783754
H	1.07816849	-3.25464665	-2.84077710
H	2.68360989	-3.00444955	-3.53519963
C	3.25420236	-1.18800261	-1.78632896
C	5.73091316	-1.03806053	-1.23976168
H	6.79622278	-1.16528534	-1.17110076
C	4.94140697	-0.11056562	-0.65337424
C	5.32870249	0.95427640	0.27581256
C	6.58257811	1.04146321	0.88438039
H	7.34734322	0.30873594	0.66734379
C	6.82242625	2.07327444	1.78917213
H	7.78562645	2.15319677	2.27592986
C	5.81002385	2.99628360	2.07096409
H	5.96707469	3.80122710	2.77542474
C	4.58679734	2.86130323	1.42252634
H	3.76474250	3.54242970	1.59914563

$[(L^2)_2Cu_3PO_4]^{3+}$

Cu	0.06357695	-2.52995814	-1.66520270
Cu	1.71361310	0.18277417	2.43334472
Cu	-1.81162442	2.44913878	-0.90371352
P	0.17387992	0.13713483	-0.15271823
S	-0.48814797	-5.10063170	2.07689736
S	2.02875652	4.49191227	0.51123849
S	-5.37615817	-0.36187114	-1.47718127
S	-0.01112115	0.61274140	-4.99161304
S	-1.80431145	1.78180081	4.92244171
S	4.68701377	-1.80954770	-0.40781542
O	-0.34464032	0.87615139	-1.32414455
O	1.07428537	-0.94167768	-0.63552799
O	0.99232490	1.07402674	0.68110634
O	-0.89075550	-0.40807009	0.61939594

N	2.77126914	1.89452223	3.10716816
N	0.29002021	1.06002582	3.49111708
N	-1.79351831	-2.79934237	1.10296190
H	-1.57745856	-1.85218425	0.70146331
N	0.06201501	-3.64479860	-0.04821154
N	3.06869118	-0.77912382	1.39363960
N	-0.09237328	-1.31926169	-3.20291373
N	-3.26610215	1.21222440	-1.36495142
N	1.80317154	-3.65250694	-2.02984621
N	-2.06036355	2.98304811	-2.89962800
N	-2.69983075	3.24740529	0.74979052
N	-1.80783235	-3.06481093	-2.30029964
N	1.72885078	-1.56957351	3.48748919
N	3.43902811	0.72241582	-0.36532186
H	2.55172398	1.18852734	-0.04693810
N	-0.29906107	3.51623364	-0.24348052
N	-1.52323842	-0.38237124	3.14384818
H	-1.13278829	-0.59889660	2.19269751
N	-3.47676425	-0.07782644	0.58396035
H	-2.43545279	-0.00783427	0.72735095
N	1.87186736	-0.06120757	-2.99942449
H	1.89688731	-0.41643391	-2.00560339
N	1.41161600	2.80796872	-1.67532079
H	0.78970353	1.97655898	-1.87220834
C	2.06341478	-4.56570843	-1.05440178
C	-3.07155237	2.30815488	-3.51311140
C	1.06359213	-4.62599344	0.01410817
C	-3.80452795	1.38089750	-2.65006195
C	2.00501933	2.73830007	3.84925686
C	-2.29172128	-2.29146310	-3.31588189
C	-1.86487900	4.00109218	1.52310423
C	-1.34347545	-1.31627432	-3.83544372

C	-3.98765436	3.07152680	1.12483887
H	-4.60955241	2.49688899	0.45592921
C	-3.89053411	0.31080149	-0.61713410
C	0.72343151	-0.35016729	-3.60010061
C	3.65294330	-0.43390052	0.25434864
C	-0.51230835	4.13059654	1.00046826
C	-2.58778691	-4.02917854	-1.76110912
H	-2.13872361	-4.63230924	-0.98780800
C	-0.81635739	-3.68573258	0.94559021
C	-0.95568679	0.66851230	3.72591207
C	0.94505251	-5.50517462	1.03688206
H	1.54266395	-6.37008488	1.26203959
C	-4.32944405	-0.83281483	1.53265082
H	-5.36143986	-0.54928280	1.29927553
C	-2.77954998	-1.00127669	3.62273066
H	-2.82337405	-0.80404419	4.69948857
C	0.65410452	2.25278390	4.13583617
C	2.53073086	-2.53482338	2.95010402
C	0.96756573	3.51815673	-0.64297797
C	-2.31143391	4.57110714	2.71524897
H	-1.63463477	5.15962484	3.31753318
C	-2.72720034	-2.53100529	3.43634856
H	-3.52074625	-2.96610915	4.05371636
H	-1.76693482	-2.90639531	3.80137006
C	-4.04987799	-0.39066772	2.98482477
H	-4.90097119	-0.71613631	3.59316384
H	-4.00096373	0.70110908	3.02710731
C	-2.96259808	-3.01078107	1.98614127
H	-3.12854368	-4.09318754	2.01680616
C	-3.59811829	-2.44888624	-3.77812861
H	-3.97191269	-1.80970997	-4.56499662
C	-3.33461480	2.48724021	-4.87167027

H	-4.13914373	1.93960403	-5.34360353
C	-4.22359130	-2.36862261	1.37025753
H	-5.08955772	-2.80223974	1.88160306
H	-4.28526689	-2.63627466	0.31157422
C	3.85567533	-4.21752014	-3.12672258
H	4.54274198	-4.05202999	-3.94468299
C	3.21891592	-5.34736779	-1.09219582
H	3.41331572	-6.06912536	-0.31103864
C	3.97667510	2.76716122	-1.61838993
H	4.79324993	3.21657702	-2.19338391
H	3.96322991	3.25248921	-0.64028945
C	2.68084907	-3.47824058	-3.04091421
H	2.42072385	-2.72857815	-3.77512599
C	3.27446181	-2.11325245	1.77085009
C	-3.62939328	4.36783795	3.11216998
H	-3.98919529	4.79676208	4.03788540
C	2.49236949	3.98065582	4.26000188
H	1.87096685	4.64149872	4.84910181
C	4.01820582	2.26304404	2.74432918
H	4.57238563	1.55091182	2.14902915
C	-3.89403256	-4.23580467	-2.18678598
H	-4.49247152	-5.01344639	-1.73369982
C	-4.48536168	3.61377818	2.30281560
H	-5.51895949	3.45391513	2.57526442
C	-1.28433516	3.82818259	-3.61226710
H	-0.48958924	4.32150613	-3.07036274
C	0.56579330	4.73760306	1.54534016
H	0.64134283	5.30050562	2.45677781
C	2.59398088	-3.81047106	3.51077964
H	3.22635848	-4.56412365	3.06471329
C	-4.93109721	0.67398306	-2.90180435
H	-5.55749414	0.68296388	-3.77556807

C	4.30355872	1.27761691	-1.43287853
H	5.33667591	1.20548893	-1.07274072
C	1.02911879	-3.09367177	5.19800388
H	0.43617747	-3.27897281	6.08250816
C	1.00513280	-1.84124478	4.59767753
H	0.41641665	-1.03110946	4.99714138
C	-1.49408410	-0.41377257	-4.83119482
H	-2.32508879	-0.26589508	-5.49536683
C	2.95271068	0.75890921	-3.59285205
H	3.11916906	0.37663548	-4.60677721
C	-4.40788679	-3.42336745	-3.20270637
H	-5.42682204	-3.55128702	-3.54285748
C	4.12248245	-5.16927910	-2.13763075
H	5.02720534	-5.76148363	-2.17779124
C	4.24132662	0.53016834	-2.78936341
H	5.07955613	0.90274622	-3.38719733
H	4.38761507	-0.54380027	-2.65384369
C	-1.49195749	4.04136496	-4.97062118
H	-0.85140598	4.71926445	-5.51687651
C	3.77867211	4.36052330	3.88309573
H	4.16949335	5.32354459	4.18439430
C	1.83047083	-4.09403263	4.63907089
H	1.86112553	-5.08119334	5.08075188
C	2.64721531	2.27271043	-3.72546716
H	3.42313924	2.68748803	-4.37747916
H	1.68538187	2.43681495	-4.21699298
C	4.55610415	3.49222929	3.11045086
H	5.55533633	3.76242872	2.79887543
C	2.67467717	3.06138059	-2.40578591
H	2.68647365	4.12846491	-2.65609008
C	-2.53627780	3.36174573	-5.60573765
H	-2.72123225	3.50725245	-6.66183019

C	-0.28885244	2.78024938	4.95216918
H	-0.22650663	3.64070330	5.59378329
C	4.11978276	-2.81879005	0.98566861
H	4.46862467	-3.82894831	1.09351515

$[(L^1)_2Cu_3SO_4]^{4+}$

Cu	0.09326086	-3.19048198	-0.10352398
Cu	-2.80271861	1.49048612	-0.17953351
Cu	2.68030881	1.67847127	0.24344302
S	3.54840572	-3.71565009	3.03709684
S	-5.20678332	-1.36346114	2.61762443
S	1.10184247	4.99253477	2.90588434
S	-4.11375086	-2.36851529	-1.90507428
S	0.09448333	4.84558959	-1.60976747
S	4.14621910	-2.27252764	-1.51506273
S	0.04497666	0.00566948	-0.33067268
O	0.89230948	1.14238667	-0.76043172
O	-0.04358777	-0.04667922	1.14939939
O	0.63247685	-1.23561059	-0.86240951
O	-1.30570628	0.17143825	-0.92051101
N	1.14991057	-4.54404400	-1.20503348
N	1.90121506	-3.23884895	1.00693959
N	1.80684330	-1.53169720	2.61538939
H	1.20034331	-0.99495242	1.97628659
N	-0.27279340	-0.08196654	4.25070317
N	-2.41615469	-1.05490313	2.33882702
H	-1.60599373	-0.83830781	1.74444995
N	-3.83375542	-0.10003118	0.72782397
N	-4.43083212	1.38849262	-1.41048728
N	0.19640591	2.36903888	2.50630424
H	0.05918655	1.56017560	1.87469320
N	1.84786939	3.24816102	1.07971828

N	3.51685573	3.14902875	-0.95700379
N	-1.05748082	-4.03550010	1.57988823
N	-1.84379744	-2.96218567	-0.69539406
N	-1.45442347	-2.00704985	-2.78050214
H	-0.51482649	-1.82320156	-2.41064476
N	0.13999546	0.06616444	-4.35957764
N	-0.84547532	2.39710427	-2.66914138
H	-1.18763762	1.47745912	-2.36258774
N	-1.61203692	3.10352529	-0.59725354
N	-3.06658352	2.81100129	1.57334426
N	2.64443250	-0.15583975	-2.55333203
H	1.97436938	0.56602317	-2.25858710
N	3.51766744	0.01711202	-0.38939624
N	3.79568727	1.19505786	1.92333061
C	0.79711582	-5.01396830	-2.42498993
H	-0.09799823	-4.58484278	-2.85726369
C	1.53893623	-5.98790710	-3.08491438
H	1.22945120	-6.33982730	-4.05947675
C	2.67954661	-6.50193899	-2.46020014
H	3.27486964	-7.26387352	-2.94599903
C	3.05031015	-6.01724648	-1.20760728
H	3.93933011	-6.38587754	-0.71430114
C	2.27006054	-5.03133018	-0.60080348
C	2.59886371	-4.41878927	0.68989388
C	3.49195290	-4.84481088	1.61716388
H	4.10026675	-5.73184212	1.61966740
C	2.26292183	-2.70736777	2.14491101
C	2.04366720	-1.03075064	3.97187976
H	2.79220197	-1.65111275	4.46663253
H	2.43800053	-0.01457873	3.92929507
C	0.75853316	-1.08271088	4.80341480
H	0.92979665	-0.80822493	5.84447592

H	0.31740925	-2.07705057	4.74725569
C	-1.70303685	-0.50416053	4.63305564
H	-1.66215881	-0.83860747	5.66992851
H	-2.33804340	0.37712528	4.54780206
C	-2.19133850	-1.59938015	3.67878467
H	-3.12208228	-2.01115256	4.07087868
H	-1.47524175	-2.41928437	3.61840807
C	-3.62872143	-0.76915796	1.83081319
C	-6.06638971	-0.61494975	1.20499504
H	-7.14045744	-0.64734886	1.15961202
C	-5.18804056	-0.03544149	0.35048366
C	-5.46683861	0.65699855	-0.91152082
C	-6.68015762	0.56073756	-1.59549382
H	-7.48715463	-0.03363888	-1.18916070
C	-6.82590416	1.21601733	-2.81644230
H	-7.75828961	1.14758096	-3.36142404
C	-5.75405868	1.94974292	-3.33490987
H	-5.83353450	2.46052545	-4.28484885
C	-4.57380110	2.01553290	-2.60190691
H	-3.71088431	2.56826644	-2.95225149
C	0.02283643	1.34397027	4.74563216
H	-0.42548950	1.42389539	5.73607620
H	1.10374042	1.45645957	4.81579253
C	-0.55454479	2.37492931	3.77302839
H	-0.49337037	3.36044782	4.23546549
H	-1.60385422	2.17563108	3.55925491
C	1.01885535	3.33986029	2.10682812
C	2.29104355	5.47221299	1.62013434
H	2.70818506	6.46324385	1.62541305
C	2.52475802	4.44754730	0.77160256
C	3.37176636	4.39325300	-0.42058983
C	3.97353778	5.50465290	-1.00907746

H	3.84661293	6.48647777	-0.57480959
C	4.72564575	5.32867164	-2.16904957
H	5.19772738	6.17972099	-2.64175576
C	4.86013549	4.05067952	-2.72041035
H	5.43410862	3.88887496	-3.62185475
C	4.24462753	2.97903286	-2.08303386
H	4.31526286	1.96789170	-2.46080604
C	-0.54931398	-4.60747360	2.69622991
H	0.52898259	-4.61615356	2.77707234
C	-1.35704067	-5.15853863	3.68724222
H	-0.91203626	-5.60841661	4.56416939
C	-2.74516805	-5.11955610	3.51825457
H	-3.40200539	-5.53763619	4.26973431
C	-3.27694400	-4.54213406	2.36728901
H	-4.34599365	-4.50531673	2.21172989
C	-2.40949410	-4.00884957	1.40892384
C	-2.86252114	-3.38961239	0.16405969
C	-4.12763339	-3.17953083	-0.28082520
H	-5.06704572	-3.40550549	0.18925571
C	-2.28607946	-2.44447489	-1.81261436
C	-1.92848533	-1.33066778	-3.99146006
H	-2.63088592	-1.98139118	-4.52150141
H	-2.45498955	-0.39509942	-3.75556261
C	-0.73547157	-1.08544838	-4.90813028
H	-1.03585878	-0.79253681	-5.91342310
H	-0.11306473	-1.97996556	-4.94978137
C	-0.38728359	1.43577936	-4.84209905
H	0.03547173	1.59578861	-5.83270624
H	-1.47348525	1.36451778	-4.90322145
C	-0.00531935	2.53740417	-3.85885827
H	-0.20692714	3.49999042	-4.33942981
H	1.06620149	2.51021345	-3.61150158

C	-0.87661008	3.28975950	-1.66284916
C	-1.54165417	4.15117699	0.32449893
C	-0.71174859	5.17192101	-0.01392040
H	-0.50172209	6.08873228	0.50455249
C	-2.38243905	3.98956537	1.51080433
C	-2.49130797	4.95222295	2.51889696
H	-1.94253590	5.88076719	2.44520129
C	-3.32165374	4.70238440	3.60980484
H	-3.42008873	5.43745521	4.39776772
C	-4.03126091	3.49879389	3.66871623
H	-4.69112001	3.27815195	4.49649328
C	-3.87997346	2.58250038	2.63090014
H	-4.41029902	1.63976389	2.62953061
C	1.61798504	-0.12058300	-4.76436105
H	1.61082478	-0.57529085	-5.75395217
H	2.07261477	0.86808672	-4.81905730
C	2.36106935	-0.97446590	-3.74476372
H	3.30668024	-1.29038183	-4.19470561
H	1.79509387	-1.87679860	-3.47480586
C	3.34931765	-0.64028531	-1.52235358
C	4.72640578	-1.89688473	0.15959897
H	5.30989293	-2.62298459	0.69437269
C	4.28837101	-0.68131403	0.55843515
C	4.47835908	0.01707426	1.82134640
C	5.28628651	-0.43974437	2.86384956
H	5.81667391	-1.37619287	2.76691747
C	5.40442069	0.33256703	4.01730360
H	6.02809724	-0.00543618	4.83403522
C	4.72114530	1.54980546	4.10615253
H	4.80386285	2.17783846	4.98172848
C	3.92696638	1.95155192	3.03824777
H	3.38313612	2.88389713	3.05778442

H	-0.20930157	-0.08873308	3.21473060
H	0.07449651	0.04133747	-3.32831400

$[(L^2)_2Cu_3SO_4]^{4+}$

Cu	-1.23954900	2.76714800	-0.14623200
Cu	-1.84478900	-2.37936400	0.01949800
Cu	3.12157100	-0.37377100	-0.09693900
S	0.00894500	-0.03065300	-0.30972900
S	-3.99574300	1.45828800	3.32084100
S	1.83826600	-3.66459300	-3.06508400
S	3.30346200	2.86531800	3.20599600
S	2.18289700	3.30216800	-3.18641600
S	0.85327800	-3.93812800	3.46240100
S	-4.08323500	0.00238000	-3.22698000
O	1.41349600	0.58128300	-0.88298500
O	-1.24425800	0.85128500	-0.89609200
O	-0.15790400	-1.57689800	-0.83654900
O	0.00024300	0.01035500	1.28787400
N	-1.36844800	-4.16239400	-0.94111300
N	-0.67797900	-3.16523900	1.45180400
N	-1.21639000	1.45539500	3.06576000
H	-0.42930300	1.31818900	2.42393500
N	-2.53699300	2.18908200	1.24917600
N	-2.94777200	-1.41251000	-1.31374500
N	0.23726600	3.18141500	-1.41024500
N	3.24228600	1.10550000	1.23773800
N	-2.98160200	3.14467500	-1.20231900
N	4.29855200	0.88560900	-1.19856800
N	3.61374800	-1.88483600	1.14357100
N	-0.21393800	4.05299400	1.02465000
N	-3.46841100	-2.07479600	1.17276000
N	-1.51118900	-1.08411000	-3.16169800

H	-0.74930700	-1.44974500	-2.57514600
N	2.71006700	-1.88297100	-1.32561300
N	-0.61402300	-1.58867800	3.22307900
H	-0.95734300	-0.86294200	2.59073900
N	1.85640600	0.48613900	3.05823700
H	1.31256700	-0.12999900	2.44129900
N	-0.11791400	1.71137500	-3.22257400
H	-0.82235500	1.24011900	-2.64025200
N	1.63642600	-0.87860500	-3.17571900
H	1.57958200	-0.01614300	-2.61875200
C	-4.10723200	2.82558900	-0.50749900
C	4.62113700	2.03698100	-0.54890900
C	-3.88380200	2.33792100	0.85439400
C	4.06358600	2.16751700	0.79773000
C	-0.51024300	-4.93972500	-0.22953000
C	0.93960300	4.52727900	0.47170800
C	3.47227600	-3.12963800	0.60351300
C	1.22618400	4.02171700	-0.86288700
C	4.13297300	-1.74522800	2.38745700
H	4.25674000	-0.73644700	2.74673500
C	2.72091300	1.29895900	2.44763600
C	0.56981900	2.66322500	-2.58883800
C	-2.67996800	-0.95308200	-2.53225900
C	2.92852700	-3.14918700	-0.74658600
C	-0.60375800	4.48891900	2.24634700
H	-1.54813400	4.11759100	2.61000700
C	-2.38831100	1.72142300	2.48517500
C	-0.27290100	-2.74352600	2.64835500
C	-4.79780400	2.03229700	1.80370500
H	-5.86883200	2.11323200	1.76528100
C	1.51039100	0.55254500	4.50374500
H	2.43839000	0.80963500	5.02556900

C	-0.38794200	-1.23896500	4.65058300
H	-0.63088200	-2.13429300	5.23289400
C	-0.13116900	-4.41658500	1.08334500
C	-4.47916600	-1.39542900	0.55708300
C	2.10201600	-1.93597100	-2.50756000
C	3.82874800	-4.26859300	1.32543300
H	3.71138700	-5.24634300	0.88082400
C	-1.37608800	-0.12643100	5.04748700
H	-1.39091600	-0.06808100	6.14043500
H	-2.38178900	-0.40001100	4.71588700
C	1.07126900	-0.84305100	4.99091000
H	1.14608100	-0.84609800	6.08342000
H	1.77120900	-1.59288800	4.61278200
C	-0.99972100	1.28059000	4.52593700
H	-1.64761700	2.00807600	5.02613300
C	1.74725500	5.42939100	1.16297200
H	2.65587000	5.79589700	0.70846400
C	5.41857900	3.00301400	-1.16555100
H	5.67802100	3.91191400	-0.64124900
C	0.46175900	1.63805600	4.86057000
H	0.51930900	1.78411400	5.94381900
H	0.71868100	2.58875300	4.38414300
C	-4.30439500	3.74121100	-3.10706000
H	-4.34989300	4.09980000	-4.12566200
C	-5.36925000	2.95995500	-1.08825400
H	-6.25544500	2.70430300	-0.52516600
C	0.00867800	-1.59142800	-5.02118100
H	0.01852200	-1.67435900	-6.11271900
H	0.00058800	-2.60814800	-4.62108300
C	-3.07632700	3.59429700	-2.47409600
H	-2.14591400	3.83518100	-2.97011900
C	-4.19839100	-0.99941500	-0.81535200

C	4.33996000	-4.12511200	2.61144700
H	4.61893200	-4.99898300	3.18488400
C	-0.03401100	-6.14433200	-0.75230600
H	0.63695600	-6.76214700	-0.17183900
C	-1.75994600	-4.54161500	-2.17791400
H	-2.44818500	-3.88175300	-2.68755200
C	0.16456400	5.38240000	2.98048300
H	-0.17212100	5.70903200	3.95400300
C	4.50128100	-2.84461800	3.15026600
H	4.91113400	-2.69993300	4.13967700
C	4.73892400	0.66852300	-2.45799600
H	4.44583200	-0.26594900	-2.91492600
C	2.57946300	-4.20819300	-1.50979100
H	2.68658200	-5.25740800	-1.30754100
C	-5.67139800	-1.12115700	1.22641300
H	-6.45614900	-0.57426400	0.72411000
C	4.24049700	3.16894800	1.68942800
H	4.85076200	4.04985000	1.60604400
C	-1.29418900	-0.88099800	-4.61949000
H	-2.12371500	-1.37597100	-5.13690900
C	-4.80088400	-2.28067600	3.15173200
H	-4.90136500	-2.64891200	4.16272100
C	-3.63312700	-2.52213500	2.44131100
H	-2.81673200	-3.08463400	2.86484000
C	2.30696000	4.23874900	-1.64441400
H	3.16701700	4.85601100	-1.45926100
C	-0.02164800	1.38520200	-4.67072900
H	-0.02763700	2.33596900	-5.21469300
C	1.36246300	5.85119800	2.43176500
H	1.98193900	6.54624800	2.98247600
C	-5.46680800	3.41921700	-2.39925000
H	-6.43763900	3.52498300	-2.86486400

C	-1.28160200	0.60046700	-5.07116000
H	-1.33454400	0.59256700	-6.16436300
H	-2.16733700	1.12804700	-4.70815900
C	5.52342100	1.59982200	-3.12626200
H	5.85733600	1.40293600	-4.13498100
C	-0.43242000	-6.53281500	-2.02864000
H	-0.06797300	-7.45981900	-2.45096300
C	-5.83132400	-1.56315500	2.53609200
H	-6.74934600	-1.35710500	3.07002500
C	1.25926600	0.62047200	-5.08402300
H	1.28415300	0.62785500	-6.17848700
H	2.15674600	1.13964800	-4.73868800
C	-1.30608100	-5.71955800	-2.75807800
H	-1.63201200	-5.99721900	-3.75035000
C	1.30914600	-0.84761600	-4.62704600
H	2.13732700	-1.33793100	-5.14993700
C	5.86941100	2.78270800	-2.46509100
H	6.48330300	3.52510900	-2.95733100
C	0.67342900	-4.99148800	2.00592300
H	1.16428700	-5.94696900	1.97594400
C	-4.95980000	-0.28140200	-1.66999900
H	-5.95244100	0.10722300	-1.53746800
